# Large-scale mutational analysis identifies UNC93B1 variants that drive TLR-mediated autoimmunity in mice and humans

**DOI:** 10.1084/jem.20232005

**Published:** 2024-05-23

**Authors:** Victoria E. Rael, Julian A. Yano, John P. Huizar, Leianna C. Slayden, Madeleine A. Weiss, Elizabeth A. Turcotte, Jacob M. Terry, Wenqi Zuo, Isabelle Thiffault, Tomi Pastinen, Emily G. Farrow, Janda L. Jenkins, Mara L. Becker, Stephen C. Wong, Anne M. Stevens, Catherine Otten, Eric J. Allenspach, Devon E. Bonner, Jonathan A. Bernstein, Matthew T. Wheeler, Robert A. Saxton, Undiagnosed Diseases Network, Maria T. Acosta, David R. Adams, Raquel L. Alvarez, Justin Alvey, Aimee Allworth, Ashley Andrews, Euan A. Ashley, Ben Afzali, Carlos A. Bacino, Guney Bademci, Ashok Balasubramanyam, Dustin Baldridge, Jim Bale, Michael Bamshad, Deborah Barbouth, Pinar Bayrak-Toydemir, Anita Beck, Alan H. Beggs, Edward Behrens, Gill Bejerano, Hugo J. Bellen, Jimmy Bennett, Jonathan A. Bernstein, Gerard T. Berry, Anna Bican, Stephanie Bivona, Elizabeth Blue, John Bohnsack, Devon Bonner, Lorenzo Botto, Lauren C. Briere, Gabrielle Brown, Elizabeth A. Burke, Lindsay C. Burrage, Manish J. Butte, Peter Byers, William E. Byrd, John Carey, Thomas Cassini, Sirisak Chanprasert, Hsiao-Tuan Chao, Ivan Chinn, Gary D. Clark, Terra R. Coakley, Laurel A. Cobban, Joy D. Cogan, Matthew Coggins, F. Sessions Cole, Heather A. Colley, Rosario Corona, William J. Craigen, Andrew B. Crouse, Michael Cunningham, Precilla D’Souza, Hongzheng Dai, Surendra Dasari, Joie Davis, Jyoti G. Dayal, Margaret Delgado, Esteban C. Dell’Angelica, Katrina Dipple, Daniel Doherty, Naghmeh Dorrani, Argenia L. Doss, Emilie D. Douine, Dawn Earl, David J. Eckstein, Lisa T. Emrick, Christine M. Eng, Marni Falk, Elizabeth L. Fieg, Paul G. Fisher, Brent L. Fogel, Jiayu Fu, William A. Gahl, Ian Glass, Page C. Goddard, Rena A. Godfrey, Andrea Gropman, Meghan C. Halley, Rizwan Hamid, Neal Hanchard, Kelly Hassey, Nichole Hayes, Frances High, Anne Hing, Fuki M. Hisama, Ingrid A. Holm, Jason Hom, Martha Horike-Pyne, Alden Huang, Yan Huang, Sarah Hutchison, Wendy Introne, Kosuke Izumi, Gail P. Jarvik, Jeffrey Jarvik, Suman Jayadev, Orpa Jean-Marie, Vaidehi Jobanputra, Emerald Kaitryn, Shamika Ketkar, Dana Kiley, Gonench Kilich, Shilpa N. Kobren, Isaac S. Kohane, Jennefer N. Kohler, Susan Korrick, Deborah Krakow, Donna M. Krasnewich, Elijah Kravets, Seema R. Lalani, Christina Lam, Brendan C. Lanpher, Ian R. Lanza, Kimberly LeBlanc, Brendan H. Lee, Richard A. Lewis, Pengfei Liu, Nicola Longo, Sandra K. Loo, Joseph Loscalzo, Richard L. Maas, Ellen F. Macnamara, Calum A. MacRae, Valerie V. Maduro, AudreyStephannie Maghiro, Rachel Mahoney, May Christine V. Malicdan, Laura A. Mamounas, Teri A. Manolio, Rong Mao, Ronit Marom, Gabor Marth, Beth A. Martin, Martin G. Martin, Julian A. Martínez-Agosto, Shruti Marwaha, Allyn McConkie-Rosell, Alexa T. McCray, Elisabeth McGee, Matthew Might, Mohamad Mikati, Danny Miller, Ghayda Mirzaa, Eva Morava, Paolo Moretti, Marie Morimoto, John J. Mulvihill, Mariko Nakano-Okuno, Stanley F. Nelson, Shirley Nieves-Rodriguez, Donna Novacic, Devin Oglesbee, James P. Orengo, Laura Pace, Stephen Pak, J. Carl Pallais, Jeanette C. Papp, Neil H. Parker, Leoyklang Petcharet, John A. Phillips, Jennifer E. Posey, Lorraine Potocki, Barbara N. Pusey Swerdzewski, Aaron Quinlan, Deepak A. Rao, Anna Raper, Wendy Raskind, Genecee Renteria, Chloe M. Reuter, Lynette Rives, Amy K. Robertson, Lance H. Rodan, Jill A. Rosenfeld, Elizabeth Rosenthal, Francis Rossignol, Maura Ruzhnikov, Marla Sabaii, Jacinda B. Sampson, Timothy Schedl, Kelly Schoch, Daryl A. Scott, Elaine Seto, Prashant Sharma, Vandana Shashi, Emily Shelkowitz, Sam Sheppeard, Jimann Shin, Edwin K. Silverman, Janet S. Sinsheimer, Kathy Sisco, Kevin S. Smith, Lilianna Solnica-Krezel, Ben Solomon, Rebecca C. Spillmann, Andrew Stergachis, Joan M. Stoler, Kathleen Sullivan, Shirley Sutton, David A. Sweetser, Virginia Sybert, Holly K. Tabor, Queenie K.-G. Tan, Amelia L.M. Tan, Arjun Tarakad, Herman Taylor, Mustafa Tekin, Willa Thorson, Cynthia J. Tifft, Camilo Toro, Alyssa A. Tran, Rachel A. Ungar, Tiina K. Urv, Adeline Vanderver, Matt Velinder, Dave Viskochil, Tiphanie P. Vogel, Colleen E. Wahl, Melissa Walker, Nicole M. Walley, Jennifer Wambach, Jijun Wan, Lee-kai Wang, Michael F. Wangler, Patricia A. Ward, Daniel Wegner, Monika Weisz Hubshman, Mark Wener, Tara Wenger, Monte Westerfield, Matthew T. Wheeler, Jordan Whitlock, Lynne A. Wolfe, Kim Worley, Shinya Yamamoto, Zhe Zhang, Stephan Zuchner, Bo Liu, Olivia Majer, Gregory M. Barton

**Affiliations:** 1 Division of Immunology and Molecular Medicine, Department of Molecular and Cell Biology, University of California, Berkeley, Berkeley, CA, USA; 2 Division of Rheumatology, Department of Medicine, University of California, San Francisco, San Francisco, CA, USA; 3 Howard Hughes Medical Institute, University of California, Berkeley, Berkeley, CA, USA; 4 Department of Pathology and Laboratory Medicine, Children’s Mercy Hospital, Kansas City, MO, USA; 5 Genomic Medicine Center, Children’s Mercy Hospital, Kansas City, MO, USA; 6 University of Missouri Kansas City School of Medicine, Kansas City, MO, USA; 7 Department of Genetics, Children’s Mercy Hospital, Kansas City, MO, USA; 8 Division of Rheumatology, Department of Pediatrics, Duke University School of Medicine, Durham, NC, USA; 9 Division of Rheumatology, Department of Pediatrics, Seattle Children’s Hospital, Seattle, WA, USA; 10 Johnson & Johnson Innovative Medicine, Spring House, PA, USA; 11 Department of Pediatrics, University of Washington School of Medicine, Seattle, WA, USA; 12 Division of Pediatric Neurology, Department of Neurology, Seattle Children’s Hospital, University of Washington, Seattle, WA, USA; 13 Center for Immunity and Immunotherapies, Seattle Children’s Research Institute, Seattle, WA, USA; 14 Stanford Center for Undiagnosed Diseases, Stanford University, Stanford, CA, USA; 15 Division of Medical Genetics, Department of Pediatrics, Stanford University School of Medicine, Stanford, CA, USA; 16 Division of Cardiovascular Medicine, Stanford University School of Medicine, Stanford, CA, USA; 17 Department of Chemistry, University of California, Berkeley, CA, USA; 18 Key Laboratory of Immune Response and Immunotherapy, Shanghai Institute of Immunity and Infection, Chinese Academy of Sciences, Shanghai, China; 19 Max Planck Institute for Infection Biology, Berlin, Germany

## Abstract

Nucleic acid–sensing Toll-like receptors (TLR) 3, 7/8, and 9 are key innate immune sensors whose activities must be tightly regulated to prevent systemic autoimmune or autoinflammatory disease or virus-associated immunopathology. Here, we report a systematic scanning-alanine mutagenesis screen of all cytosolic and luminal residues of the TLR chaperone protein UNC93B1, which identified both negative and positive regulatory regions affecting TLR3, TLR7, and TLR9 responses. We subsequently identified two families harboring heterozygous coding mutations in *UNC93B1*, *UNC93B1*^*+/T93I*^ and *UNC93B1*^*+/R336C*^, both in key negative regulatory regions identified in our screen. These patients presented with cutaneous tumid lupus and juvenile idiopathic arthritis plus neuroinflammatory disease, respectively. Disruption of UNC93B1-mediated regulation by these mutations led to enhanced TLR7/8 responses, and both variants resulted in systemic autoimmune or inflammatory disease when introduced into mice via genome editing. Altogether, our results implicate the UNC93B1-TLR7/8 axis in human monogenic autoimmune diseases and provide a functional resource to assess the impact of yet-to-be-reported *UNC93B1* mutations.

## Teaser

This work reports a mutagenesis screen of the TLR chaperone protein UNC93B1 that reveals regulatory regions affecting TLR3, TLR7, and TLR9 responses. Autoimmune patients are identified with coding variants in UNC93B1 that map to regions identified by the screen, and the introduction of these human variants into mice leads to TLR-driven autoimmune disease.

## Introduction

A subset of the mammalian Toll-like receptors (TLRs) recognizes various forms of nucleic acids: TLR3 binds double-stranded RNA, TLR7 and TLR8 bind single-stranded RNA fragments, TLR9 binds single-stranded DNA, and TLR13 binds single-stranded RNA sequences present in bacterial ribosomal RNA (Fitzgerald and Kagan, 2020). These specificities enable broad recognition of diverse microbes but also expose the host to potential responses against self-nucleic acids that can lead to autoimmune and inflammatory diseases such as lupus and psoriasis (Lind et al., 2022). Roles for TLR7, TLR8, and TLR9 in the pathology of such diseases are now well established in humans and mice, and suppression of TLR activation is being pursued therapeutically as a treatment for several autoimmune diseases (Lind et al., 2022; McWhirter and Jefferies, 2020).

Compartmentalized activation of TLRs within endolysosomes limits responses to extracellular self-nucleic acids released from dead or dying cells. Multiple mechanisms collectively establish this compartmentalization, including nucleases that degrade self-ligands before they activate TLRs (Al-Mayouf et al., 2011; Baum et al., 2015; Gavin et al., 2018; Napirei et al., 2000; Pawaria et al., 2015; Sisirak et al., 2016; Yasutomo et al., 2001), a requirement for ectodomain cleavage by proteases within acidic endolysosomes (Ewald et al., 2008, 2011; Park et al., 2008), and trafficking of the receptors themselves (Barton et al., 2006; Fukui et al., 2009, 2011; Mouchess et al., 2011; Stanbery et al., 2020). The relative importance of each of these mechanisms for the avoidance of self-nucleic acids is different for each TLR. For example, avoidance of self-DNA by TLR9 requires the activity of several DNAses (Baum et al., 2015; Gavin et al., 2018; Sisirak et al., 2016), while avoidance of self-RNA by TLR7 and TLR8 (the latter is functional in humans but not in mice) appears to be more dependent on the levels of the receptors themselves (Deane et al., 2007; Guiducci et al., 2013; Pisitkun et al., 2006; Subramanian et al., 2006). Increases in TLR7 or TLR8 gene copy number can induce autoimmune disease in mice (Deane et al., 2007; Guiducci et al., 2013; Pisitkun et al., 2006; Subramanian et al., 2006), and failures in X chromosome inactivation, where TLR7 and TLR8 are located, have been proposed as a cause of SLE in humans (Souyris et al., 2018). Whether similar mechanisms control the activity of the other nucleic acid–sensing TLRs remains largely unexplored.

While the mechanisms that control trafficking and localization of the nucleic acid–sensing TLRs remain incompletely understood, the multipass transmembrane protein UNC93B1 has emerged as a central player in recent years. UNC93B1, which is highly conserved across species, binds TLRs in the ER (Brinkmann et al., 2007; Kim et al., 2008), facilitates their loading into COPII vesicles (Lee et al., 2013), and traffics with TLRs to endolysosomes (Kim et al., 2008; Majer et al., 2019a). In mice and humans with null mutations in UNC93B1, the UNC93B1-dependent TLRs (TLR3, TLR5, TLR7, TLR8, TLR9, TLR11, TLR12, and TLR13) are destabilized and fail to leave the ER (Brinkmann et al., 2007; Lee et al., 2013; Pelka et al., 2018). Humans lacking UNC93B1 function are susceptible to certain viral infections due to defective TLR function (Casrouge et al., 2006; Zhang et al., 2020).

Recent studies indicate that UNC93B1 can also engage mechanisms that dampen TLR signaling and limit responses to self-nucleic acids. A single amino-acid substitution in the UNC93B1 N-terminal cytosolic tail (D34A) leads to increased TLR7 responses (Fukui et al., 2009, 2011). One hypothesis is that this mutation favors the export of TLR7 from the ER at the expense of TLR9. Mice with this *Unc93b1*^*D34A*^ mutation develop TLR7-dependent autoimmunity, suggesting that UNC93B1 carefully tunes levels of TLR7 to avoid responses to self-RNA (Fukui et al., 2011). We recently described a distinct mechanism of TLR7 regulation that involves the UNC93B1 C-terminal tail (Majer et al., 2019a). Residues in this region are necessary for SYNTENIN-1 binding to UNC93B1-TLR7 complexes. SYNTENIN-1 is involved in the sorting of cargo into multivesicular bodies, and we hypothesized that this mechanism is critical for TLR7 turnover and termination of signaling. This work also identified a ubiquitylated lysine residue (K333) located on one of the UNC93B1 cytosolic loops that when mutated leads to increased TLR7 signaling. Mutation of three amino acids in the UNC93B1 C-terminal tail in mice caused TLR7-dependent autoimmune disease (Majer et al., 2019a), and a coding variant in the same region has been linked to exfoliative cutaneous lupus in dogs (Leeb et al., 2020). Intriguingly, these N- and C-terminal mutations do not increase TLR9 responses (Fukui et al., 2009; Majer et al., 2019a), further illustrating the distinct regulation of these related receptors by UNC93B1. One potential explanation for this differential effect is our recent observation that TLR9 is released from UNC93B1 in endosomes, while TLR7 remains associated (Majer et al., 2019b), which may subject TLR7 to additional regulatory mechanisms.

While UNC93B1 can clearly influence TLR function, our understanding of how this regulation is mediated remains in its infancy. Recent structures of UNC93B1 bound to TLR3 and TLR7 have identified potential key residues involved in UNC93B1-TLR interactions (Ishida et al., 2021), but the significance of these and other residues for UNC93B1 and TLR function, especially in the context of avoiding responses to self nucleic acids, remains largely unexplored. Moreover, whether disruptions in UNC93B1-mediated regulation of TLRs can drive autoimmune disease in humans is an open question. The identification of patients with coding variants in *TLR7* and *TLR8* has established the relevance of dysregulation of these receptors for human autoimmune disease (Brown et al., 2022), but equivalent coding variants in *UNC93B1* that alter TLR responses to self-nucleic acids have not yet been described.

Here, we have addressed these unanswered questions through large-scale, systematic mutagenesis of UNC93B1. Our results identify multiple and, in some cases, distinct regions that positively and negatively control the function of TLR3, TLR7, and TLR9. In parallel, we identify patients with autoimmune diseases carrying germline mutations in *UNC93B1* corresponding to regulatory regions identified in our screen, and we show that these mutations can drive autoimmunity when introduced into mice. Two recent papers, published while our manuscript was in preparation, also describe patients with systemic lupus who harbor inborn mutations in *UNC93B1* (Mishra et al., 2024; Wolf et al., 2024). Altogether, our results provide a comprehensive map of the regulatory landscape of UNC93B1 and establish the relevance of this landscape for human autoimmune disease.

## Results

### Mutagenesis screen identifies multiple regions of UNC93B1 that alter TLR responses

**Figure 1.**
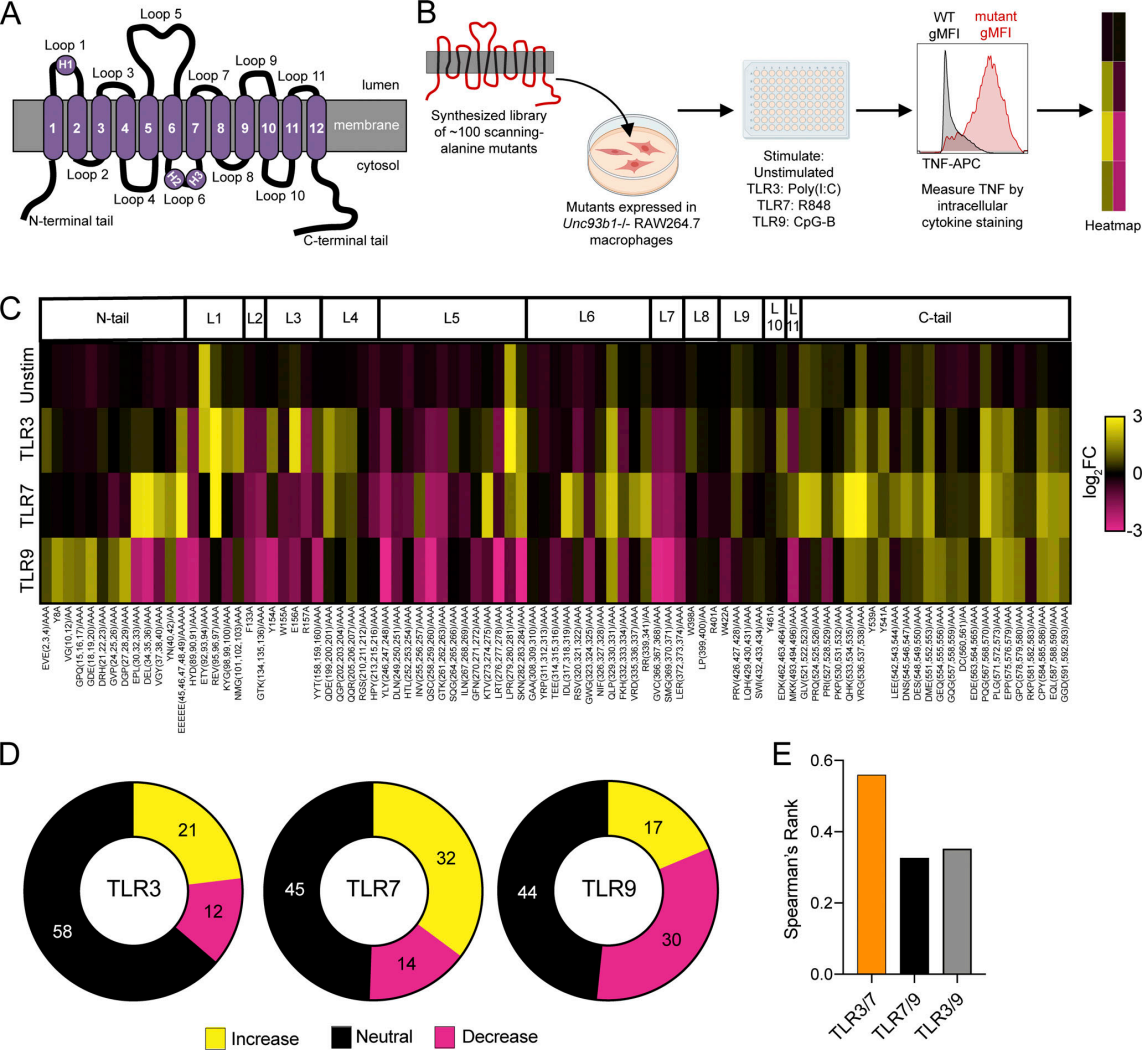
**A scanning-alanine mutagenesis screen reveals distinct domains of UNC93B1 that regulate endosomal TLR signaling. (A)** Schematic of UNC93B1 depicting the membrane topology, transmembrane domains (1–12), connecting loops, and N- and C-terminal cytosolic tails. Helices within loops 1 and 6 are labeled as H1, H2, and H3, in accordance with the recent structures of UNC93B1 (Ishida et al., 2021). **(B)** Overview of UNC93B1 scanning–alanine mutagenesis screen workflow. **(C)** Heatmap summarizing the effect of each UNC93B1 mutant on signaling by TLR3, TLR7, and TLR9. Unstim, unstimulated; TLR3, stimulation with Poly(I:C) (20 µg ml^−1^); TLR7, stimulation with R848 (10 ng ml^−1^); TLR9, stimulation with CpG-B (45 nM). Data are shown as log_2_FC of the average TNF gMFI of triplicate wells for a given mutant/stimulation condition compared to corresponding wild-type controls. Data are representative of at least two independent experiments. **(D)** Quantification of UNC93B1 mutants that signal equivalently to wild-type (neutral) or confer a twofold or greater increase or decrease in signaling by the indicated TLR. **(E)** Spearman’s rank correlation coefficient between changes in signaling capacity of indicated TLR pairs.

**Figure S1.**
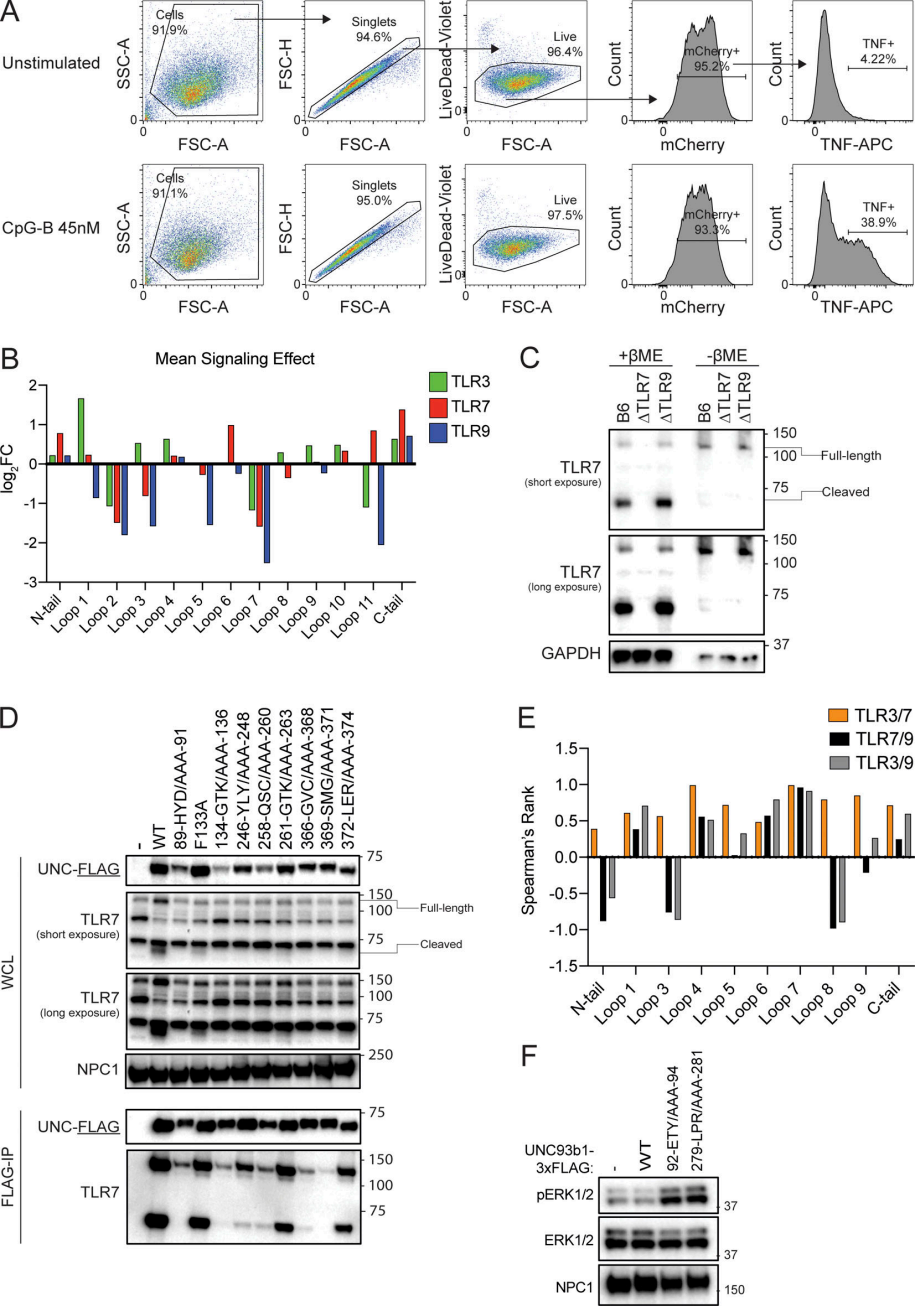
**A scanning-alanine mutagenesis screen reveals distinct domains of UNC93B1 that regulate endosomal TLR signaling. (A)** Representative flow cytometry data and gating strategy for scanning alanine mutagenesis screen. **(B)** Mean signaling effect of each tail and loop domain of UNC93B1 on individual TLRs. **(C)** Validation of TLR7 monoclonal antibody. Immunoblot of lysates of BMMs derived from WT (B6), TLR7-deficient, or TLR9-deficient mice run in the presence or absence of β-mercaptoethanol (βME) to reduce the disulfide bond that holds the two TLR7 receptor fragments together. **(D)** Analysis of UNC93B1-FLAG and associated TLR7 in mutants that reduce signaling by TLR3, TLR7, and TLR9. UNC93B1-FLAG levels were measured by immunoblot of whole cell lysates of *Unc93b1*^*−/−*^ RAW264.7 macrophages reconstituted with the indicated mouse *Unc93b1* alleles, or after FLAG immunoprecipitation. UNC93B1-associated endogenous TLR7 was measured by immunoblot. NPC1 serves as a loading control. **(E)** Spearman’s rank correlation in signaling intensity between indicated TLRs across domains of UNC93B1, based on mutagenesis screen data shown in Fig. 1 C. **(F)** Immunoblot demonstrating phospho-ERK levels in *Unc93b1*^*−/−*^ RAW264.7 macrophages reconstituted with indicated variants of murine UNC93B1. Data are representative of two independent experiments. Source data are available for this figure: SourceData FS1.

To identify residues in UNC93B1 that regulate TLR function, we synthesized a library of scanning-alanine mutants in which one to three adjacent amino acids were mutated to alanine residues across the murine UNC93B1 protein sequence. We focused our analysis on the tail and loop regions based on the predicted topology of UNC93B1 (Fig. 1 A), which we refined based on recent cryoEM structures of UNC93B1 bound to TLR3 and TLR7 (Ishida et al., 2021). Retroviral vectors encoding each mutant were introduced into a RAW macrophage line in which the endogenous *Unc93b1* alleles had been previously disrupted by Cas9 genome editing (Majer et al., 2019a, 2019b). After selection of lines stably expressing each mutant, we stimulated with ligands for TLR3 (polyinosinic:polycytidylic acid (poly(I:C)), TLR7 (R848), and TLR9 (CpG-B ODN) and measured TNF production by intracellular cytokine staining (ICS) and flow cytometry (Fig. 1 B and Fig. S1 A). We tested a range of doses to ensure that we could detect any increases or decreases in responses relative to wildtype UNC93B1. We could not probe TLR8 function because specific ligands for mouse TLR8 are not available, nor is it clear that TLR8 is functional in mice. Stimulation with LPS, the ligand for UNC93B1-independent TLR4, served as a control.

As shown in Fig. 1, C and D, the screen identified many mutations that alter TLR3, TLR7, and TLR9 responses. In many instances, adjacent mutants behaved similarly and could be used to define larger functional regions of UNC93B1 (Fig. S1 B). Several such regions were required for the function of all three TLRs tested (Fig. 1 C and Fig. S1 B), but in most cases these mutations did not dramatically reduce UNC93B1 protein levels, suggesting that they do not simply destabilize the protein (Fig. S1 D). Those mutations that most severely impacted TLR signaling also destabilized the interaction between UNC93B1 and TLR7 (Fig. S1 D). For example, a group of mutants in loop 5 (residues 246–263) mostly reduced responses to ligands for all three TLRs. These residues comprise a long loop on the luminal side of the membrane that contains two N-linked glycosylation sites. Based on the published structures, these residues in loop 5 were not predicted to make direct contacts with TLR3 or TLR7; however, we previously showed that more distal residues in loop 5 (residues 270–284) were involved in interactions with TLR9 (Majer et al., 2019b), and the screen results confirm the importance of these residues for TLR9 function. Mutations in residues 366–371 also resulted in reduced responses for each TLR and disrupted interactions with TLR7 (Fig. 1 C and Fig. S1, B and D). These residues form a small loop between transmembrane domain 7 (TM7) and TM8 that sits below loop 5, suggesting that the loop 5 and loop 7 phenotypes may be mechanistically related.

Many mutations increased TLR responses, and the largest number of these led to increased TLR7 responses (Fig. 1 D). The magnitude of the increase in response was also generally larger for TLR7 than for TLR3 or TLR9 (Fig. 1 C). We identified multiple regions of UNC93B1 that when mutated led to increased TLR7 responses: a portion of the N-terminal tail (residues 30–49), residues 95–97 of helix 1 in loop 1, residues 199–207 in loop 4, residues 273–284 in loop 5, most of loop 6, and most of the C-terminal tail. Some of these regions have already been implicated in TLR7 regulation; in particular, mutations in the N- and C-terminal tails have been previously shown to increase TLR7 signaling through distinct mechanisms (Fukui et al., 2009; Majer et al., 2019a). However, the other regions represent new regions not yet implicated in TLR7 regulation. The sheer number of mutations that result in increased TLR7 responses underscores the complex regulation of this receptor and is consistent with the finding that TLR7 remains associated with UNC93B1 upon arrival in endosomes, which may render it subject to additional UNC93B1-mediated regulation (Majer et al., 2019b).

Some of the mutated regions that increased TLR7 responses also led to increased TLR3 responses, and overall the effects of mutations correlated better between TLR3 and TLR7 than between other pairs (Fig. 1 E and Fig. S1 E). Mutations in loop 1 increased signaling by both receptors, although more mutations in this loop affected TLR3 than TLR7. Mutations in loop 4 (residues 199–207) and loop 5 (residues 279–284) also increased responses by both TLR3 and TLR7. Mutations of other regions resulted in much more selective effects on either TLR3 or TLR7 responses. For example, mutations in the N-terminal tail (residues 30–42) and loop 6 (residues 317–341) increased TLR7 responses but did not increase TLR3 or TLR9 responses (Fig. 1 C and Fig. S1 B). This selective effect on TLR7 has been previously described for mutations in the N-terminal region (Fukui et al., 2009) but not for loop 6. Mutations in the short loop 3 that connects TM3 and TM4 selectively increased TLR3 responses while reducing TLR7 and TLR9 responses (Fig. 1 C and Fig. S1 B).

Relatively fewer mutations led to increased TLR9 responses, and the overall magnitude of increase was lower for TLR9 compared with TLR7 and TLR3 (Fig. 1, C and D; and Fig. S1 B). Accordingly, the effect of mutations showed little correlation between TLR7 and TLR9 or TLR3 and TLR9 (Fig. 1 E and Fig. S1 E). In fact, TLR9 responses were often reduced by mutations that led to increased TLR7 and/or TLR3 responses. For example, we identified mutations in regions of the N-terminus, loop 1, loop 3, loop 5, and loop 6 that increased TLR7 and/or TLR3 signaling yet reduced TLR9 responses (Fig. 1 C). This counter-regulation has been previously reported for the N-terminal tail (Fukui et al., 2009), but these results demonstrate that the opposing regulation of TLR9 by UNC93B1 extends beyond that particular regulatory region.

Despite the relative paucity of mutants leading to increased TLR9 responses, several regions stood out as mediators of negative regulation: a portion of the N-terminal tail (residues 8–29) and clusters of residues throughout the C-terminal tail (Fig. 1 C). The effect of mutating the N-terminal residues was selective for TLR9, while the C-terminal region clearly plays a broader regulatory role that impacts all TLRs tested.

Perhaps the most striking result from the screen is that almost every mutation in the C-terminal tail led to increased responses by one or more of the TLRs (Fig. 1 C and Fig. S1 B). We previously described how mutations in C-terminal residues 521–553 resulted in increased TLR7 signaling due at least in part to failure to recruit SYNTENIN-1 to the UNC93B1-TLR7 complex (Majer et al., 2019a). The effect of these mutations was greater on TLR7 than on TLR3 or TLR9 and in some cases resulted in TNF production in the absence of ligand. The screen also revealed that the more distal residues of the C-terminal tail may also contribute to TLR regulation via overlapping or distinct mechanisms. Mutation of residues 554–561 led to a selective increase in TLR9 signaling, while mutation of residues 567–593 generally increased signaling by all three TLRs.

Finally, several mutations resulted in the production of TNF in the absence of stimulation, most notably residues 92–94 in helix 1 of loop 1 and residues 279–281 in loop 5 (Fig. 1 C). Cells expressing these mutants displayed elevated levels of phospho-ERK1/2 without exogenous stimulation (Fig. S1 F), consistent with chronic TLR activation. Interestingly, in both cases, these residues are adjacent to residues that when mutated lead to increased TLR7 and TLR3 responses (Fig. 1 C).

In summary, we used scanning alanine mutagenesis to define multiple regions of UNC93B1 that, when mutated, decrease or increase the responses of TLR3, TLR7, and TLR9 to their ligands. TLR7 was most affected, but distinct regions were identified that specifically regulate each TLR.

### Identification of humans with inborn UNC93B1 variants that increase TLR responses to nucleic acid ligands

**Figure 2.**
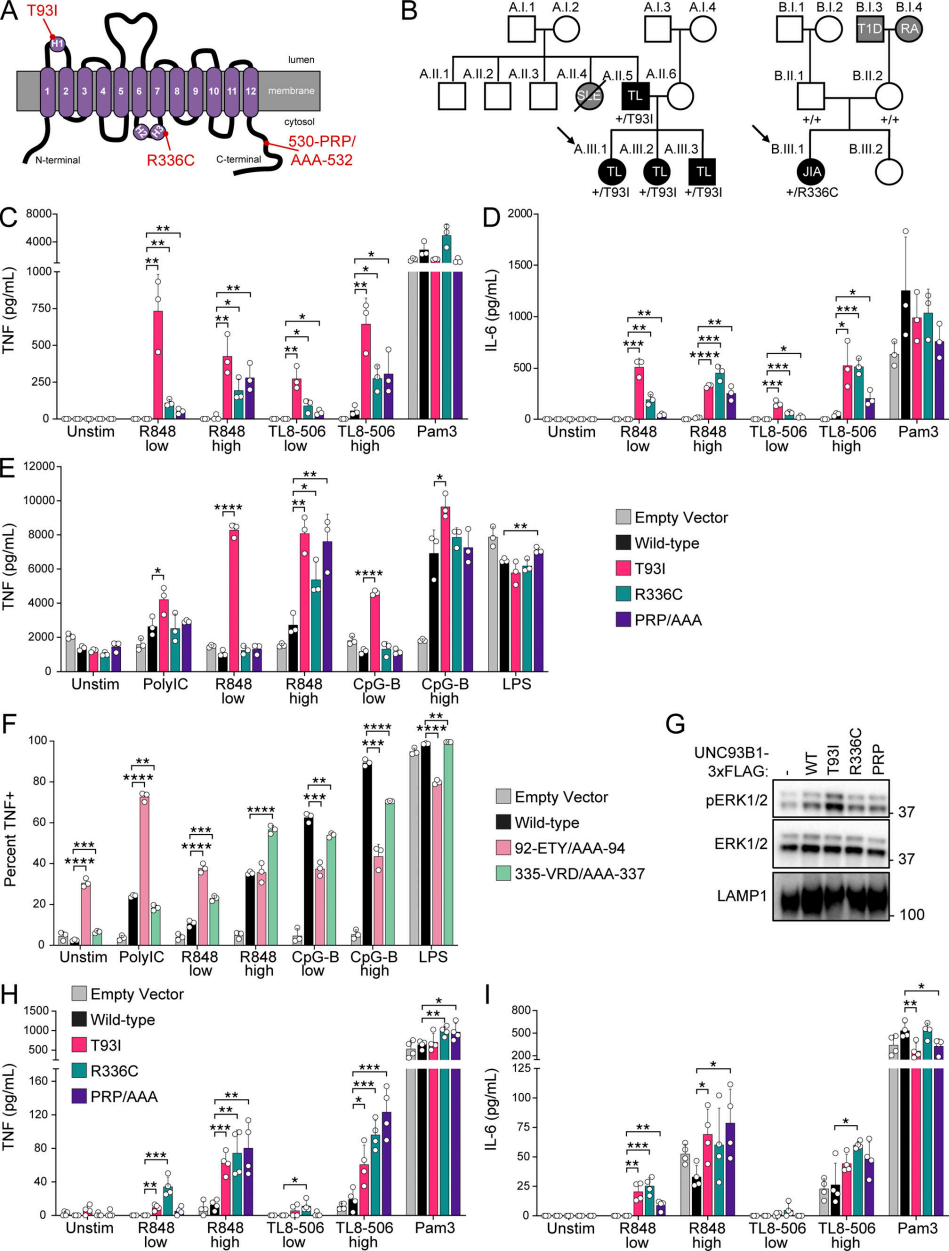
**Human UNC93B1 variants enhance endosomal TLR signaling. (A)** Diagram of UNC93B1 depicting positions of the two human variants. H1, H2, and H3 indicate helices, as described in Fig. 1. **(B)** Pedigrees of families A and B with *UNC93B1* variants. Squares, males; circles, females; horizontal lines connecting individuals and vertical nodes emanating therefrom represent parents and progeny, respectively; diagonal line crossing symbol represents the deceased individual. Black shading denotes autoimmune or inflammatory disease segregating with *UNC93B1* genotype; gray shading denotes autoimmune or inflammatory disease in individuals who were not genotyped. Diagonal arrows denote sequenced probands, and *UNC93B1* genotypes are listed below each symbol for all sequenced individuals. SLE, systemic lupus erythematosus; TL, tumid lupus; JIA, juvenile idiopathic arthritis; RA, rheumatoid arthritis; T1D, type 1 diabetes. **(C and D)** TNF (C) or IL-6 (D) production from PMA-differentiated human THP-1 cells (*UNC93B1*^*−/−*^ clone D6) reconstituted with the indicated human *UNC93B1* alleles and stimulated overnight with low R848 (1.3 µg ml^−1^), high R848 (4 µg ml^−1^), low TL8-506 (67 ng ml^−1^), high TL8-506 (200 ng ml^−1^), or Pam3CSK4 (10 ng ml^−1^). Cytokine production was measured in supernatants by LEGENDPlex assay. **(E)** TNF production from mouse RAW264.7 macrophages (*Unc93b1*^*−/−*^) reconstituted with the indicated human *UNC93B1* alleles and stimulated for 8 h with Poly(I:C) (20 µg ml^−1^), low R848 (20 ng ml^−1^), high R848 (200 ng ml^−1^), low CpG-B (5 nM), high CpG-B (67 nM), or LPS (0.5 ng ml^−1^). Cytokine production was measured in supernatants by LEGENDPlex assay. **(F)** Triple-alanine UNC93B1 mutants from UNC93B1 mutagenesis screen corresponding to human *UNC93B1* variants. Data show the percentage of TNF-positive cells determined by ICS of *Unc93b1*^*−/−*^ RAW264.7 macrophages reconstituted with the indicated mouse *Unc93b1* alleles and stimulated for 6 h with Poly(I:C) (20 µg ml^−1^), low R848 (4 ng ml^−1^), high R848 (10 ng ml^−1^), low CpG-B (45 nM), high CpG-B (100 nM), or LPS (5 ng ml^−1^). **(G)** Immunoblot demonstrating basal phospho-ERK1/2 levels in *Unc93b1*^*−/−*^ RAW264.7 macrophages expressing the indicated human UNC93B1 variants. Data are representative of at least two independent experiments. **(H and I)** TNF (H) or IL-6 (I) production from PMA-differentiated wild-type human THP-1 cells (i.e., with intact endogenous *UNC93B1* genes) ectopically expressing the indicated human *UNC93B1* alleles, stimulated overnight with low R848 (1.3 µg ml^−1^), high R848 (4 µg ml^−1^), low TL8-506 (67 ng ml^−1^), high TL8-506 (200 ng ml^−1^), or Pam3CSK4 (10 ng ml^−1^). Cytokine production was measured in supernatants by LEGENDPlex assay. Data are mean ± SD of (C–F) triplicate or (H and I) quadruplicate technical replicates, representative of at least two independent experiments. P value determined by unpaired two-tailed Student’s *t* test: *P ≤ 0.05, **P ≤ 0.01, ***P ≤ 0.001, ****P ≤ 0.0001. Source data are available for this figure: SourceData F2.

Uncontrolled TLR7, TLR8, or TLR9 activation can lead to responses to self-nucleic acids and autoimmunity. Thus, we next considered whether any of the regulatory regions identified by our scanning alanine mutagenesis screen may be relevant for human autoimmune diseases. As part of ongoing efforts at several institutions to sequence pediatric patients with early onset or severe autoimmune or autoinflammatory disease, we identified two probands with coding variants in *UNC93B1* (Fig. 2, A and B). Proband A.III.1 is a now-21-year-old female, who along with her two siblings and father developed early-childhood onset cutaneous tumid lupus, with no symptoms of systemic lupus disease as of last visit. Proband A.III.1 (along with her family members) was found by whole exome sequencing to be heterozygous for a c.278, C>T substitution in *UNC93B1* (NM_030930.4) resulting in a p.Thr93Ile missense mutation in loop 1 (*UNC93B1*^*+/T93I*^). Proband B.III.1 is a now-13-year-old female who presented at age 3 with an unusual confluence of inflammatory arthritis (extended oligoarticular juvenile idiopathic arthritis), a neuroinflammatory movement disorder, and cutaneous tumid and chillblain lupus rashes and was later found to have elevated blood monocyte CD169 expression suggestive of high serum type I IFN activity (Biesen et al., 2008; Sakumura et al., 2023; York et al., 2007). Proband B.III.1 was found by whole genome sequencing to be heterozygous for a de novo c.1006, C>T substitution in *UNC93B1* resulting in a p.Arg336Cys missense mutation in loop 6 (*UNC93B1*^*+/R336C*^). More detailed clinical descriptions of each proband can be found in Data S2. Importantly, neither the *UNC93B1*^*T93I*^ nor the *UNC93B1*^*R336C*^ alleles have been reported in the Genome Aggregation Database.

**Figure S2.**
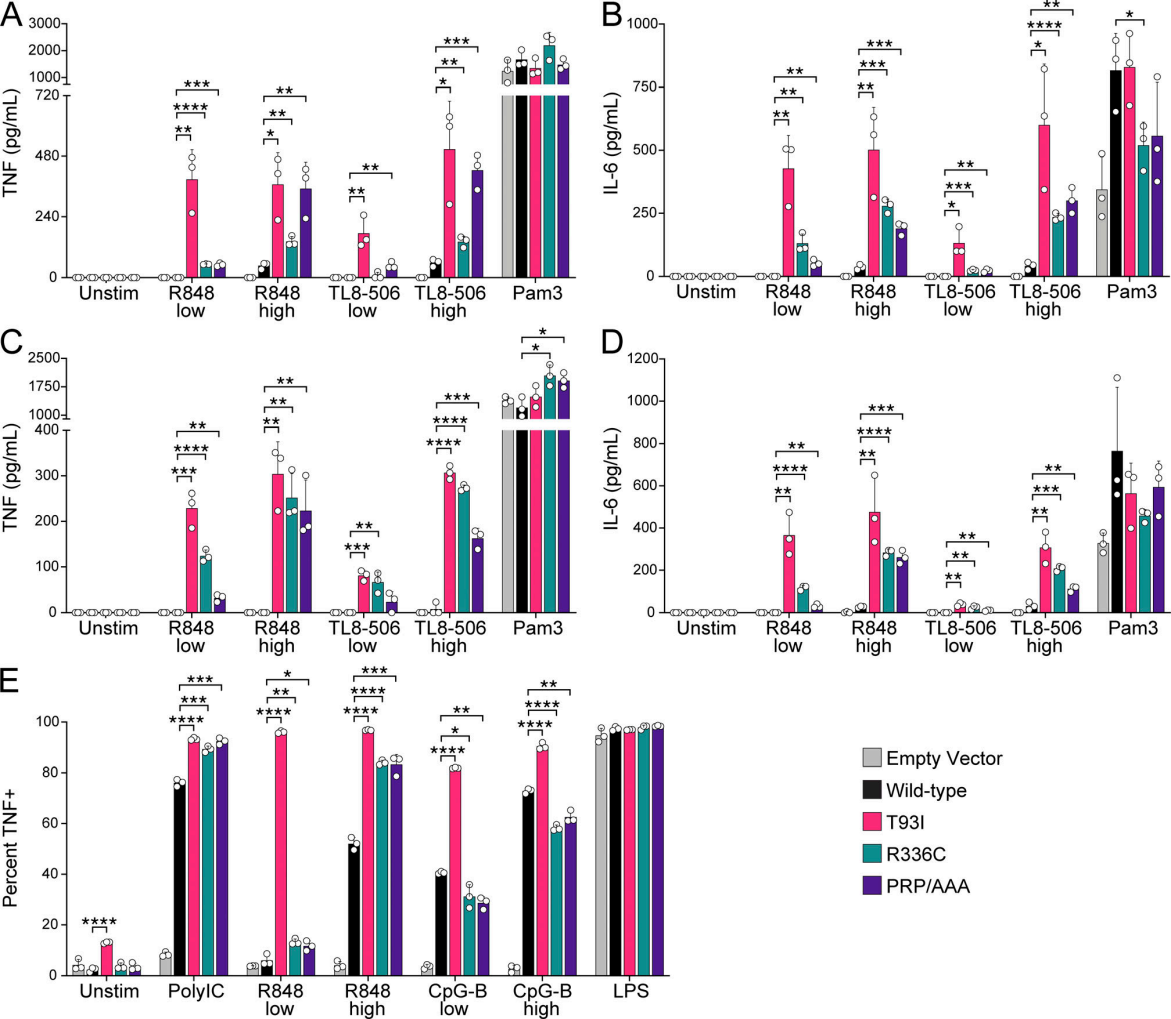
**Human UNC93B1 variants enhance endosomal TLR signaling. (A–D)** TNF (A and C) or IL-6 (B and D) production from PMA-differentiated human THP-1 cells (*UNC93B1*^*−/−*^ clone [A and B] D9 or clone [C and D] E5) reconstituted with the indicated human *UNC93B1* alleles and stimulated overnight with low R848 (1.3 µg ml^−1^), high R848 (4 µg ml^−1^), low TL8-506 (67 ng ml^−1^), high TL8-506 (200 ng ml^−1^), or Pam3CSK4 (10 ng ml^−1^). Cytokine production was measured in supernatants by LEGENDPlex assay. **(E)** ICS of TNF from mouse RAW264.7 macrophages (*Unc93b1*^*−/−*^) reconstituted with the indicated human *UNC93B1* alleles and stimulated for 6 h with Poly(I:C) (20 µg ml^−1^), low R848 (20 ng ml^−1^), high R848 (200 ng ml^−1^), low CpG-B (67 nM), high CpG-B (200 nM), or LPS (2 ng ml^−1^). Data are mean ± SD of triplicate technical replicates, representative of at least two independent experiments. P value determined by unpaired two-tailed Student’s *t* test: *P ≤ 0.05, **P ≤ 0.01, ***P ≤ 0.001, ****P ≤ 0.0001.

Both variants change amino acids that are conserved between mice and humans and map to regions identified in the mutagenesis screen as increasing TLR responses when mutated to alanine. Therefore, we tested whether cells expressing a given variant had altered responses to TLR ligands that could underlie the autoimmunity observed in these patients. We stably expressed hUNC93B1^WT^, hUNC93B1^T93I^, and hUNC93B1^R336C^ in three independent THP-1 monocyte clones in which the endogenous alleles of *UNC93B1* had been disrupted by Cas9 editing. As a control, we also generated THP-1 cells expressing hUNC93B1 with amino acids 530–532 (Pro, Arg, Pro) mutated to alanine (hUNC93B1^PRP/AAA^), which corresponds to the mutation in the C-terminal tail of mice that we previously showed leads to TLR7-driven autoimmunity (Majer et al., 2019a). Production of TNF and IL-6 in response to the TLR7/8 ligand R848 and the TLR8 ligand TL8-506 was higher in cells expressing each of the human variants relative to cells expressing UNC93B1^WT^ (Fig. 2, C and D; and Fig. S2, A–D). Strikingly, the TLR7/8 response of UNC93B1^T93I^ expressing cells was even greater than that of UNC93B1^PRP/AAA^ expressing cells. In contrast, responses to the TLR2 ligand Pam3CSK4 were equivalent between cells expressing UNC93B1^WT^ and each of the variants, as expected for this UNC93B1-independent TLR.

Because THP-1 cells do not express TLR3, TLR7, or TLR9, we expressed each human variant in *Unc93b1*^*−/−*^ murine RAW cells to examine signaling by these TLRs. Again, we observed heightened production of TNF in response to the TLR7 ligand R848 by cells expressing each of the human variants, and responses were again particularly elevated in UNC93B1^T93I^ expressing cells (Fig. 2 E and Fig. S2 E). TLR3 responses were also slightly enhanced by the UNC93B1^T93I^ variant. TLR9 responses were unchanged or slightly decreased in UNC93B1^R336C^ expressing cells, while they were increased in UNC93B1^T93I^ expressing cells relative to UNC93B1^WT^. Expression of the UNC93B1^PRP/AAA^ variant led to strongly enhanced TLR7 responses, with minimal effects on other TLR responses, as we previously reported (Majer et al., 2019a).

The responses measured for each human variant in THP-1 and RAW cells correlated with the mutagenesis data of corresponding residues from the scanning-alanine mutagenesis screen of murine UNC93B1 in RAW cells. Mutation of residues 335–337 (Val, Arg, Asp) to alanines led to increased TLR7 responses but did not increase TLR3 or TLR9 (Fig. 2 F), in accordance with the phenotype of the UNC93B1^R336C^ human variant. Interestingly, as noted earlier, cells expressing UNC93B1 with amino acids 92–94 (Glu, Thr, and Tyr) each mutated to alanine produced high levels of TNF even in the absence of stimulation (Fig. 2 F, Fig. 1 C, and Fig. S1 F). TNF production by unstimulated UNC93B1^T93I^ expressing cells was not as robust (Fig. S2 E), but we did consistently observe elevated levels of phospho-ERK1/2 in the cells, suggestive of chronic basal TLR activation (Fig. 2 G). It is likely that the UNC93B1^92-ETY/AAA-94^ triple mutation leads to a more severe disruption of the mechanism by the UNC93B1^T93I^ variant.

Finally, because the patients carrying the UNC93B1^T93I^ and UNC93B1^R336C^ variants are heterozygous for these alleles, we considered whether these variants led to increased TLR responses in the presence of UNC93B1^WT^. To address this issue, we expressed each variant in wild-type THP-1 cells with endogenous UNC93B1 intact. Responses to TLR7/8 ligands were increased in THP-1 cells expressing UNC93B1^T93I^ and UNC93B1^R336C^, relative to cells ectopically expressing UNC93B1^WT^ (Fig. 2, H and I). Heterozygosity of the UNC93B1^T93I^ and UNC93B1^R336C^ variants thus appears sufficient to increase TLR7/8 responses in myeloid cells, consistent with the notion that these heterozygous mutations may contribute to disease in the patients.

### Human UNC93B1 variants increase TLR signaling through distinct mechanisms

**Figure 3.**
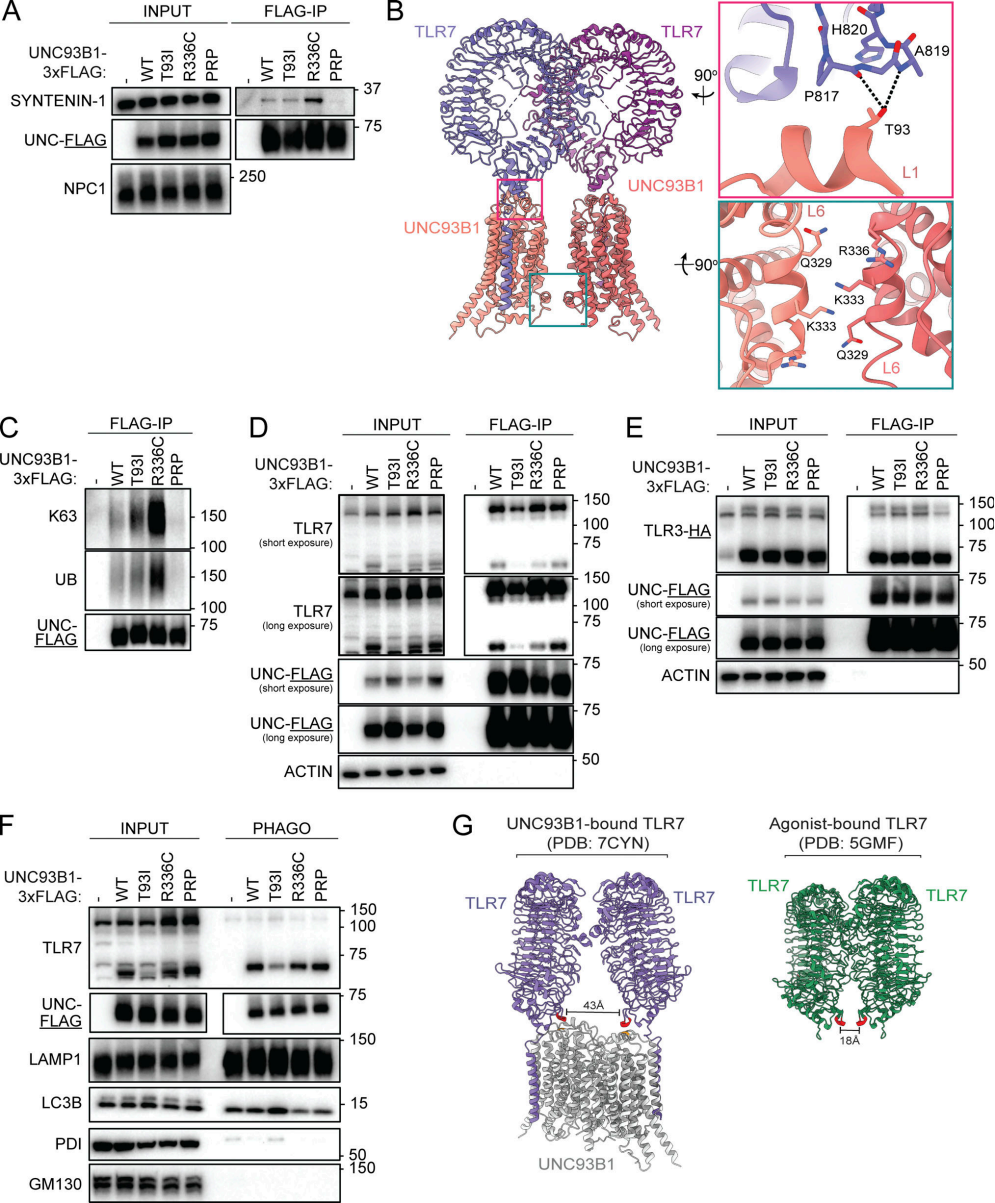
**Human UNC93B1 variants disrupt known negative regulatory mechanisms. (A)** Immunoprecipitation of UNC93B1-3xFLAG from lysates of unstimulated *Unc93b1*^*−/−*^ RAW264.7 macrophage cells expressing the indicated variants, followed by immunoblot to measure levels of associated SYNTENIN-1. **(B)** Modeled structure of UNC93B1-TLR7 dimers (PDB ID: 7CYN) with insets depicting loop 1 (top right) and loop 6 (bottom right). Dashed lines indicate predicted hydrogen bonds. **(C)** Immunoprecipitation of UNC93B1-3xFLAG from lysates of *Unc93b1*^*−/−*^ RAW264.7 macrophage cells expressing the indicated variants, followed by immunoblot for total or K63-linked ubiquitylation (UB). **(D)** Immunoprecipitation of UNC93B1-3xFLAG from lysates of *Unc93b1*^*−/−*^ RAW264.7 macrophage cells expressing the indicated variants, followed by immunoblot for endogenous TLR7. **(E)** Immunoprecipitation of UNC93B1-3xFLAG from lysates of *Unc93b1*^*−/−*^ RAW264.7 macrophage cells expressing the indicated variants, followed by immunoblot for TLR3-HA. **(F)** Purification of intact phagosomes from *Unc93b1*^*−/−*^ RAW264.7 macrophage cells expressing the indicated variants of UNC93B1-3xFLAG. **(G)** Modeled structure of TLR7 bound to UNC93B1 versus TLR7 bound to agonist. Residue A819 of TLR7 is highlighted in red and residue T93 of UNC93B1 is highlighted in orange. Data are representative of at least three independent experiments in A and C–F. Source data are available for this figure: SourceData F3.

**Figure S3.**
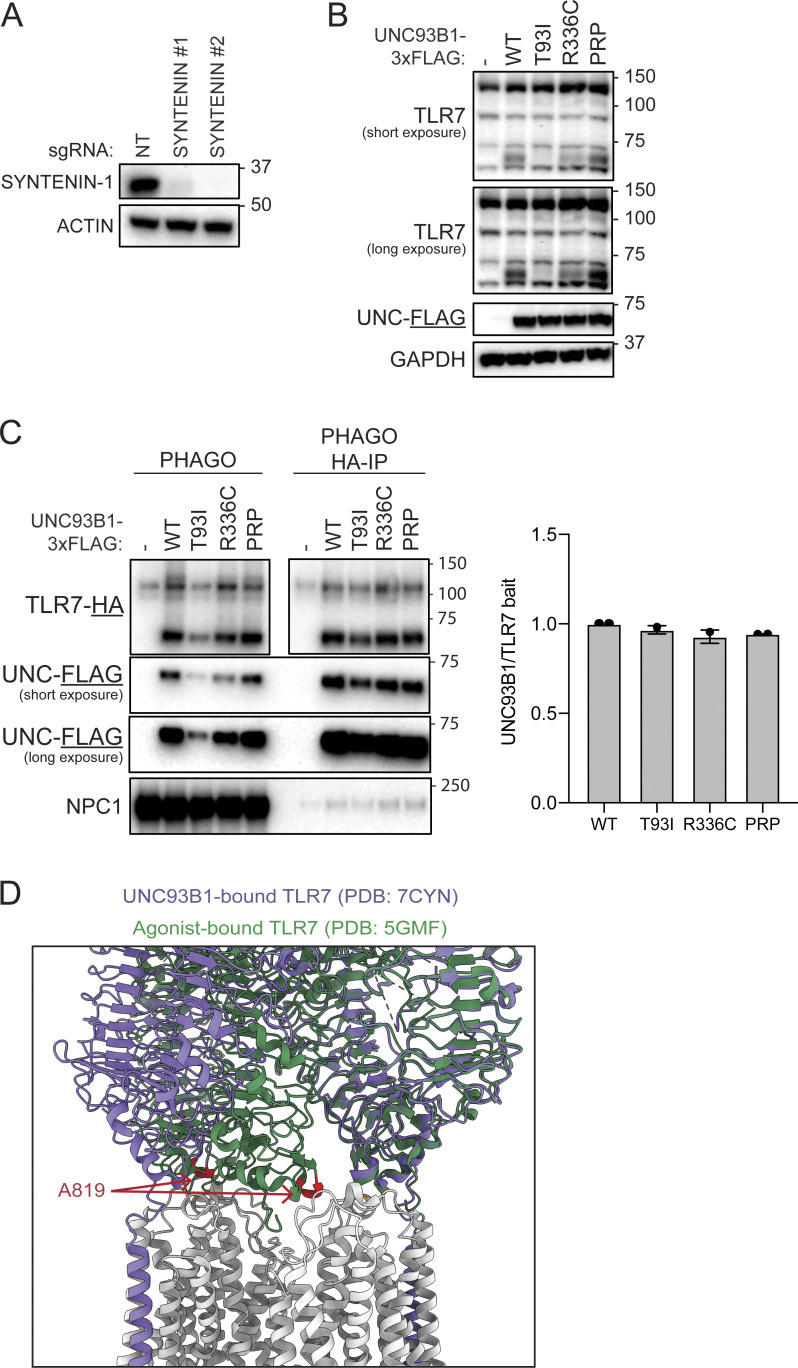
**Human UNC93B1 variants disrupt known negative regulatory mechanisms. (A)** Validation of SYNTENIN-1 antibody. Immunoblot of lysates of Hoxb8-Macrophages endogenously expressing Cas9 and transduced with the indicated gRNAs. **(B)** Immunoblot for endogenous TLR7, UNC93B1-3xFLAG, or ACTIN in lysates of *Unc93b1*^*−/−*^ RAW264.7 macrophage cells expressing the indicated UNC93B1-FLAG variants after sorting for even expression. **(C)** Immunoprecipitation of TLR7-HA from purified phagosomes of *Unc93b1*^*−/−*^ RAW264.7 macrophage cells expressing the indicated UNC93B1-3xFLAG variants. Quantification of UNC93B1 that co-precipitates with TLR7 in two independent experiments plotted on the right. **(D)** Overlay of the modeled structures of TLR7 when bound to UNC93B1 and ligand. Data are representative of at least three independent experiments unless indicated otherwise. Source data are available for this figure: SourceData FS3.

Having identified human *UNC93B1* coding variants that increase TLR responses to nucleic acid ligands, we next examined how these variants mediate these effects. We initially focused on the mechanism we recently described wherein residues in the UNC93B1 C-terminal tail are required for recruitment of SYNTENIN-1 to UNC93B1 (Majer et al., 2019a). Immunoprecipitation of UNC93B1 variants from RAW cells revealed that the UNC93B1^T93I^ interaction with SYNTENIN-1 was similar to that of wild-type UNC93B1, while UNC93B1^PRP/AAA^ exhibited significantly reduced association (Fig. 3 A and Fig. S3 A), consistent with our previous findings (Majer et al., 2019a). In contrast, the interaction between UNC93B1^R336C^ and SYNTENIN-1 was enhanced (Fig. 3 A). These results were unexpected, as effective recruitment of SYNTENIN-1 to UNC93B1 was previously shown to mediate the silencing of TLR7. Thus, UNC93B1^R336C^ and UNC93B1^T93I^ appear to dysregulate TLR7 signaling through mechanisms distinct from UNC93B1^PRP/AAA^ and from each other.

The UNC93B1^R336C^ variant maps to an α-helix in the cytosolic loop 6 of UNC93B1 (Fig. 2 A and Fig. 3 B). This α-helix contains a key lysine residue (Lys333) which is subject to K63-linked ubiquitylation and when mutated to arginine results in increased TLR7 responses (Majer et al., 2019a). In the TLR7-UNC93B1 2:2 dimer structure, the two-loop 6 α-helices lie parallel to each other at the interface of the UNC93B1-UNC93B1 dimer with each Lys333 residue facing downward, consistent with them serving as substrates for an E3 ligase and playing a key regulatory role (Fig. 3 B, lower inset). We considered whether the R336C mutation altered the function of this regulatory helix. Intriguingly, analysis of UNC93B1^R336C^ from RAW cells revealed that ubiquitylation of UNC93B1^R336C^ was increased relative to UNC93B1^WT^ (Fig. 3 C). These results are consistent with the increased association of UNC93B1^R336C^ with SYNTENIN-1, as SYNTENIN-1 has been shown to bind ubiquitin (Rajesh et al., 2011). While increased ubiquitylation and SYNTENIN-1 association resulting in increased TLR7 signaling is initially counter-intuitive, these results support a model in which the UNC93B1^R336C^ variant disrupts regulatory steps downstream of UNC93B1 ubiquitylation and SYNTENIN-1 binding, such that ubiquitylated UNC93B1^R336C^-TLR7 complexes accumulate. We note that in the TLR7-UNC93B1 structure, Arg336 is in close proximity to Gln329, suggesting the possibility of hydrogen bond formation between the side chain guanidinium group of Arg336 and the carbonyl oxygen of Gln329 (Fig. 3 B, lower inset). Disruption of this interaction by the R336C variant may interfere with downstream sorting events.

The UNC93B1^T93I^ variant did not show differences relative to UNC93B1^WT^ in terms of ubiquitylation or SYNTENIN-1 interaction, which is not surprising as this residue is located on a luminal loop that connects TM1 and TM2 (Fig. 2 A). Analysis of the TLR7-UNC93B1 cryoEM structure revealed that Thr93 lies on an α-helix that runs parallel to the membrane and sits in close proximity to the base of the TLR7 ectodomain (Fig. 3 A, upper inset). Thr93 forms apparent hydrogen bond contacts with the backbone carbonyl and amide groups of Pro817 and Ala819 in TLR7, respectively. Mutation of Thr93 to Ile would abolish these contacts, potentially reducing the stability of the UNC93B1-TLR7 interaction. To test this possibility directly, we immunoprecipitated UNC93B1^T93I^ from RAW cells and measured associated TLR7 by immunoblot. As our structural analyses suggested, less TLR7 was bound by UNC93B1^T93I^ than UNC93B1^WT^ (Fig. 3 D and Fig. S3 B). Notably, the reduction was evident for both full-length, ER-resident TLR7 and the cleaved form of the receptor that is present in endosomes. Levels of the cleaved receptor were similarly reduced in whole-cell lysates, consistent with reduced export of TLR7 from the ER (Fig. 3 D). The reduced interaction was specific to TLR7, as the interaction between UNC93B1^T93I^ and TLR3 was unaffected (Fig. 3 E).

How does weakened interaction between TLR7 and UNC93B1 lead to increased TLR7 signaling? One hypothesis is that the release of TLR7 from UNC93B1 in endosomes frees the receptor from UNC93B1-mediated negative regulation and receptor turnover. However, accumulation of cleaved TLR7 was not apparent in UNC93B1^T93I^ cells, either in whole cell lysates or purified phagosomes (Fig. 3, D and F). We also considered that a minor pool of cleaved TLR7 dissociated from UNC93B1 could confer elevated signaling capacity, so we directly tested whether TLR7 immunoprecipitated from phagosomes was associated with less UNC93B1 in UNC93B1^T93I^ cells versus UNC93B1^WT^ cells. However, all variants of UNC93B1 equally co-precipitated with TLR7 (Fig. S3 C), arguing against the hypothesis that more TLR7 is released from UNC93B1^T93I^ in endosomes.

An alternative hypothesis for how UNC93B1^T93I^ confers elevated signaling is that the weakened association between the ectodomain of TLR7 and UNC93B1 grants the TLR7 ectodomain greater conformational flexibility and thus an increased capacity for ligand binding and dimerization. In favor of this hypothesis, comparative analysis of the structures of TLR7 ectodomains bound to UNC93B1 (in the absence of ligand) versus TLR7 ectodomains bound to agonist revealed a substantial conformational change upon ligand-induced dimerization (Fig. 3 G and Fig. S3 D). Notably, the Ala819 residues of TLR7, which are predicted to form hydrogen bonds with Thr93 of UNC93B1, are 43 Å apart when bound to UNC93B1 but move to within 18 Å of each other when bound to the ligand. This 25 Å translocation suggests that the association of TLR7 with UNC93B1 may prevent TLR7 from binding to the ligand and dimerizing, a feature noted by Ishida et al. (2021) when first reporting the TLR7-UNC93B1 structure. By disrupting this hydrogen bonding, the UNC93B1^T93I^ variant may relax this structural inhibition and enable TLR7 to more easily bind ligand and dimerize while still associated with UNC93B1.

### Human UNC93B1 variant knock-in mice develop systemic autoimmune disease

**Figure 4.**
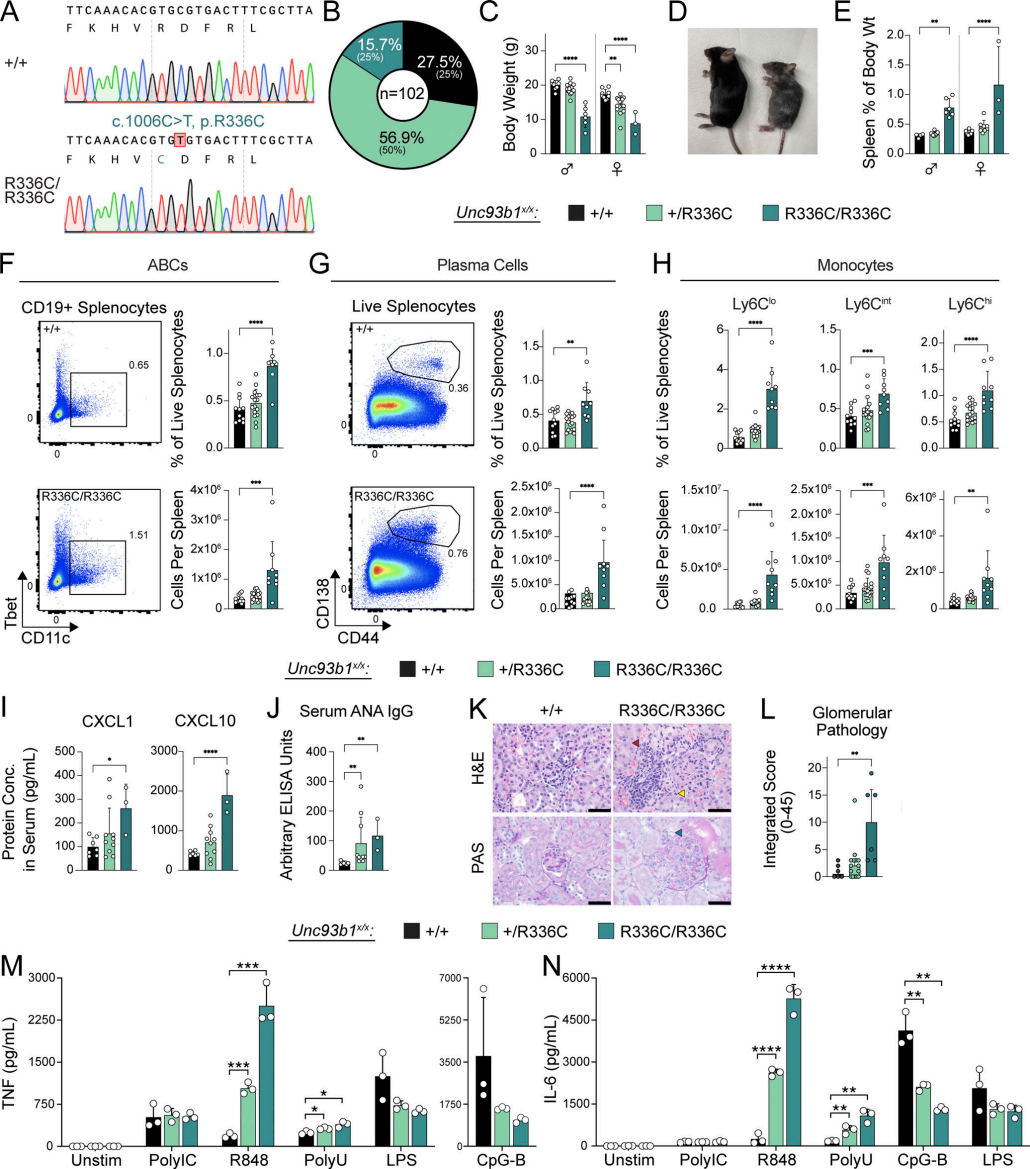
***Unc93b1***^***R336C***^ **knock-in mice develop systemic autoimmune pathology. (A)** Sanger chromatograms depicting C1006T nucleotide substitution in the endogenous murine *Unc93b1* locus, resulting in amino acid substitution R336C. **(B)** Observed percentages of pups of the indicated genotypes surviving to weaning age; expected Mendelian percentages are indicated in parentheses. **(C–J)** Phenotyping of *Unc93b1*^*+/+*^, *Unc93b1*^*+/R336C*^, and *Unc93b1*^*R336C/R336C*^ mice: **(C)** Body weight of 5-wk-old mice. **(D)** Representative image of 13–15-wk-old-mice upon sacrifice. **(E)** Normalized spleen weight of 13–15-wk-old mice. **(F and G)** Representative flow cytometry plots, frequency among live splenocytes, and absolute number of splenic ABCs (F) and plasma cells (G). **(H)** Frequency and absolute number of splenic monocytes. **(I)** Serum CXCL1 and CXCL10 concentration. **(J)** Serum ANA IgG level by ELISA. **(K)** Representative hematoxylin/eosin (H&E) staining (top panels) and periodic acid-Schiff (PAS) staining (bottom panels) from 13–15-wk-old *Unc93b1*^*R336C*^ mice. Interstitial leukocyte accumulation, mesangial matrix expansion, and endocapillary hypercellularity are highlighted by red, yellow, and blue arrowheads, respectively. **(L)** Blinded pathologic scoring of kidney sections from 13–15-wk-old *Unc93b1*^*R336C*^ mice. Symbols overlying bars represent values from individual mice (C, E–J, and L). P values were obtained using a one-way ANOVA with Sidak’s (C) or Dunnett’s (E–I) correction for multiple comparisons, or a Kruskal–Wallis test with Dunn’s correction for multiple comparisons (J and L). Data are pooled from at least nine litters (B and C); pooled from four independent experiments using mice from two independently generated knockin lines (E–H); are plotted from one and representative of two independent experiments (I and J); or are pooled from three independent experiments (L). **(M and N)** BMMs were prepared from mice of the indicated genotypes and stimulated overnight with PolyIC (20 µg ml^−1^), R848 (4 ng ml^−1^), ssPolyU complexed with DOTAP (12.5 µg ml^−1^), CpG-B (50 nM), or LPS (2 ng ml^−1^). Cytokine production was measured in supernatants by LEGENDPlex assay. Data are mean ± SD of triplicate technical replicates, representative of at least two independent experiments, and P values were determined using an unpaired two-tailed Student’s *t* test. *P ≤ 0.05, **P ≤ 0.01, ***P ≤ 0.001, ****P ≤ 0.0001.

**Figure S4.**
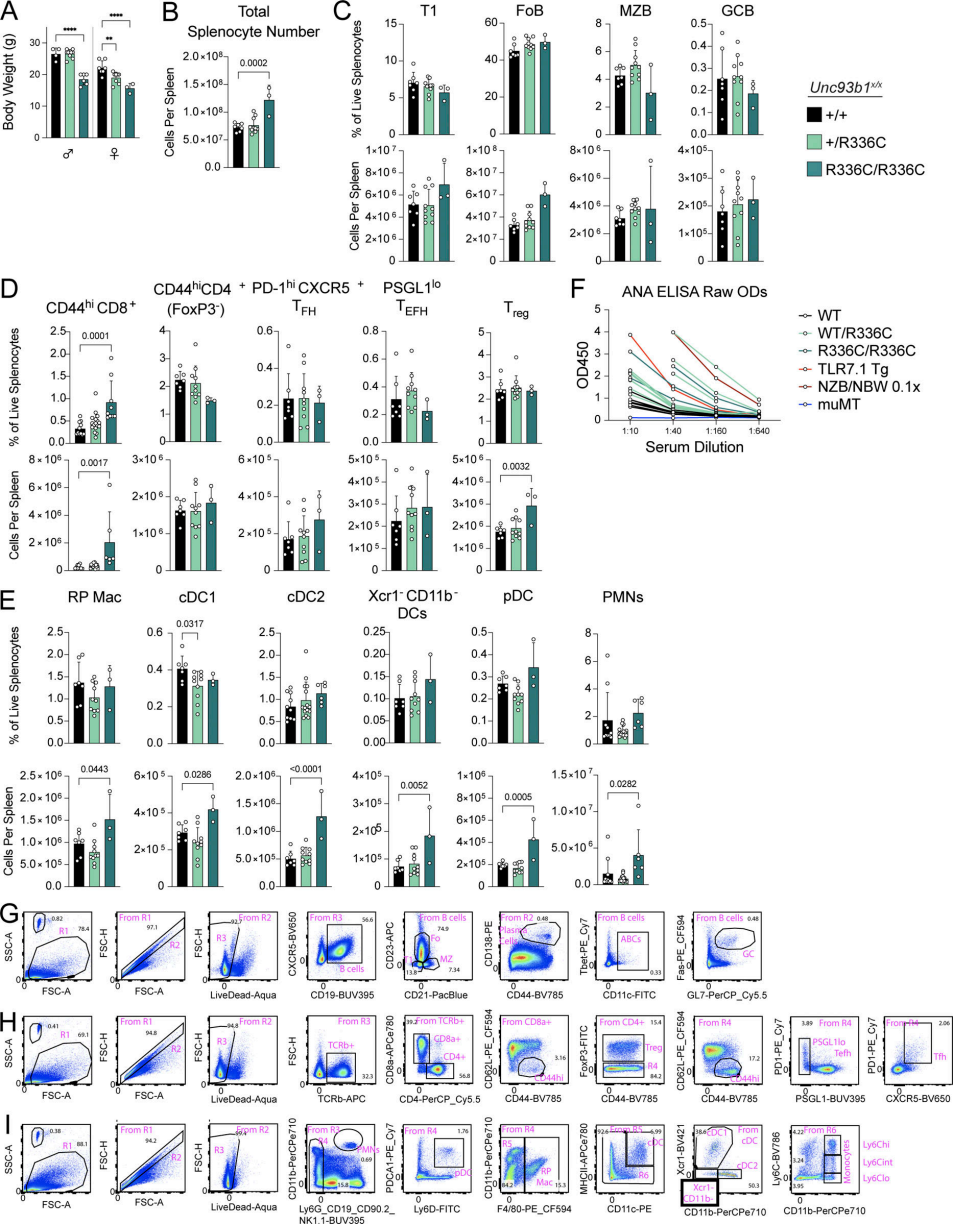
**Additional phenotyping of *Unc93b1***^***R336C***^ **mice and gating strategies used to subset splenic leukocytes. (A)** Body weight of 13–15-wk-old mice. **(B)** Absolute splenocyte counts in *Unc93b1*^*R336C*^ mice. **(C–E)** Frequency and number of splenocyte subsets from *Unc93b1*^*R336C*^ mice: (C) B lineage cells, (D) T lineage cells, (E) myeloid cells. **(F)** Raw ANA ELISA OD curves. Each line represents serum from an individual mouse. NZB/NBW serum was derived from 9-mo-old mice and was used at a starting dilution of 1:100 (with subsequent 1:400, 1:1,600, and 1:6,400 dilutions). Curve points with an OD value above the detection limit, or with an OD value incrementally inconsistent with prior values in the dilution series, were omitted. In A–E, mean with SD are plotted, and symbols represent values from individual mice. P values in A–E were obtained using a one-way ANOVA. **P ≤ 0.01, ****P ≤ 0.0001. **(G–I)** Gating strategies for (G) B lineage, (H) T lineage, and (I) myeloid lineage cells, as shown for a representative *Unc93b1*^*+/+*^ mouse. T1, Transitional 1 B cells; FoB, follicular naive B cells; MZB, marginal zone B cells; GCB, germinal center B cells; T_FH_, T-follicular helper cells; T_EFH_, T-extrafollicular B helper cells; T_reg_, regulatory T cells; RP Mac, red pulp macrophages; cDC, conventional dendritic cells; pDC, plasmacytoid dendritic cells; PMN, polymorphonuclear leukocytes, also known as neutrophils.

Next, we generated knock-in mice using Cas9 genome editing to test directly whether the increased TLR signaling observed in cells expressing UNC93B1 coding variants is capable of driving autoimmunity. We introduced a c.1006, C>T substitution in *Unc93b1* (NP_062322.3) to generate *Unc93b1*^*R336C*^ mutant founder mice (Fig. 4 A), backcrossed these mice to C57BL/6 mice, and then intercrossed the progeny to generate *Unc93b1*^*+/+*^, *Unc93b1*^*+/R336C*^, and *Unc93b1*^*R336C/R336C*^ mice. *Unc93b1*^*R336C/R336C*^ mice were born below the expected Mendelian frequency (Fig. 4 B) and exhibited markedly lower body weights at 5 wk relative to *Unc93b1*^*+/+*^ littermates (Fig. 4 C), and this discrepancy persisted into adulthood (Fig. 4 D and Fig. S4 A). Analysis of 13–15-wk-old mice revealed splenomegaly in *Unc93b1*^*R336C/R336C*^ mice (Fig. 4 E), as well as cellular phenotypes consistent with systemic autoimmunity, including expansion of CD11c^+^ age-associated B cells (ABCs), plasma cells, and monocytes (Fig. 4, F–H). Serum chemokine analysis also revealed elevated CXCL1 and CXCL10 levels in *Unc93b1*^*R336C/R336C*^ homozygous animals, the latter suggestive of elevated type I and/or type II interferon activity in vivo (Fig. 4 I) (Cui et al., 2024). Antinuclear antibodies (ANA) were also detected in sera of these animals (Fig. 4 J), and this, together with the ABC and plasma cell expansion, suggests that the UNC93B1^R336C^ mutation activates B cells in addition to myeloid cells in vivo. Analysis of kidneys from the same animals revealed evidence of glomerulonephritis, including expansion of cells and matrix in the mesangium; disorganized glomerular capillary tufts with fragmented cell nuclei (tuft karyorrhexis); and glomerular/interstitial leukocyte accumulation (Fig. 4, K and L; and Table S3). We did not find evidence of overt paw swelling in *Unc93b1*^*R336C*^ mice, suggesting that the allele does not drive frank inflammatory arthritis on the C57BL/6 genetic background. Bone marrow–derived macrophages (BMMs) from *Unc93b1*^*R336C/R336C*^ mice recapitulated the increased responses to TLR7 ligands but not to TLR3, TLR9, or TLR4 ligands (Fig. 4, M and N), suggesting that the phenotypes observed in these animals may be TLR7-mediated. Altogether, these results provide direct evidence that the UNC93B1^R336C^ variant is sufficient to drive systemic autoimmune disease.

Because the patient we identified is heterozygous for the UNC93B1^R336C^ variant, we were interested in whether *Unc93b1*^*+/R336C*^ mice show evidence of disease. Indeed, some of the phenotypes observed in *Unc93b1*^*R336C/R336C*^ mice were also present in *Unc93b1*^*+/R336C*^ mice, albeit to a lesser degree (Fig. 4). BMMs from *Unc93b1*^*+/R336C*^ mice also produced more cytokines than *Unc93b1*^*+/+*^ mice when stimulated with TLR7 but not TLR3, TLR4, or TLR9 ligands (Fig. 4, M and N). Interestingly, when stratified by sex, female *Unc93b1*^*+/R336C*^ mice did show a statistically significant reduction in weight compared with female *Unc93b1*^*+/+*^ littermates (Fig. 4 C). Additionally, both male and female *Unc93b1*^*+/R336C*^ mice exhibited significant increases in serum ANA IgG (Fig. 4 J). Cellular phenotypes and glomerular pathology trended higher in *Unc93b1*^*+/R336C*^ mice but did not reach statistical significance (Fig. 4, F–I and L). Finally, the observed kidney pathology also affected a fraction of heterozygotes (Fig. 4 L and Table S3). Thus, heterozygosity for the UNC93B1^R336C^ variant is sufficient to induce disease in mice, apparently to a greater extent in female mice given the aforementioned body weight differences. Notably, proband B.III.1 heterozygous for the UNC93B1^R336C^ variant is female.

**Figure 5.**
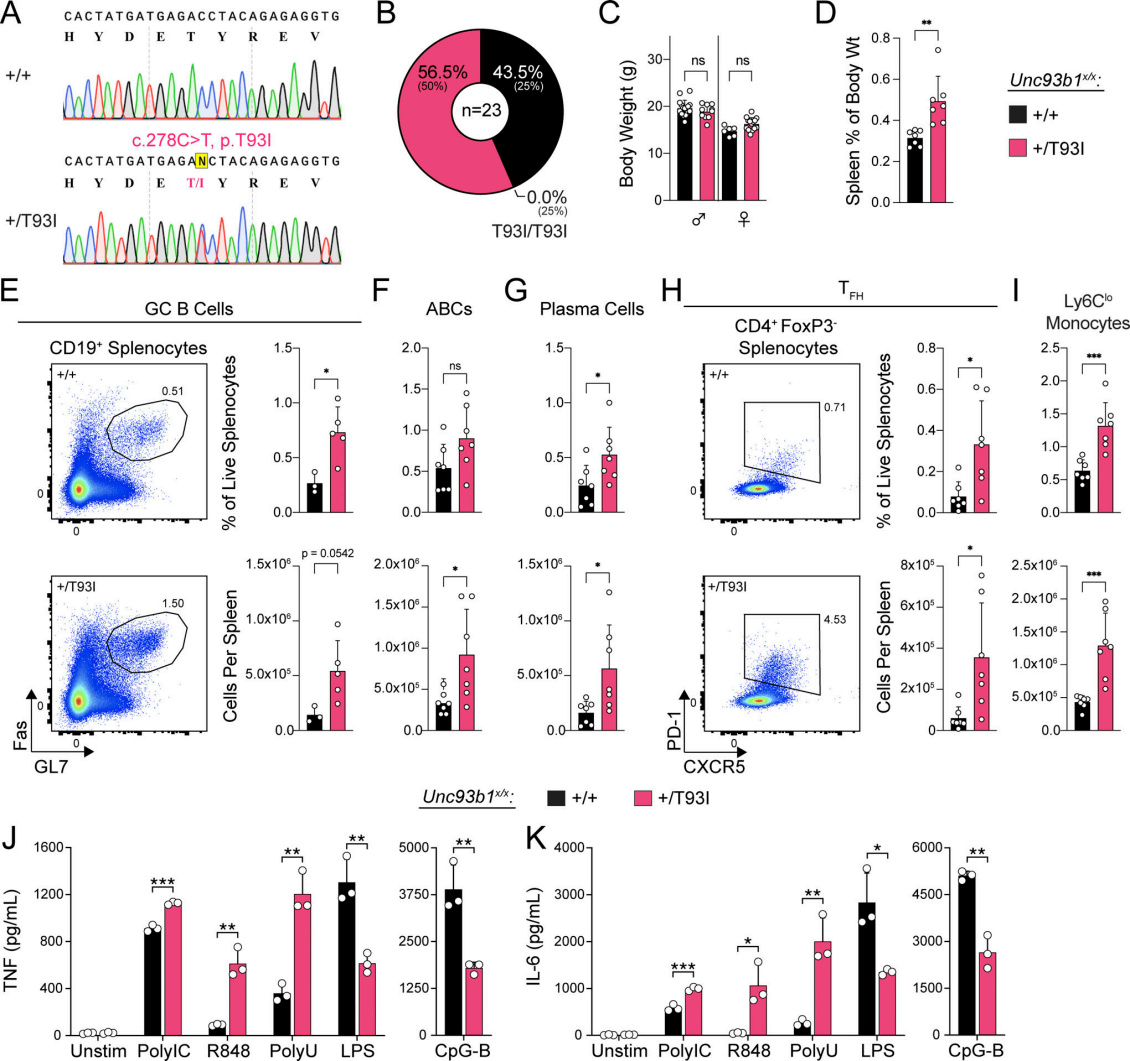
***Unc93b1***^***T93I ***^**knock-in mice develop severe systemic inflammation. (A)** Sanger chromatograms depicting C278T nucleotide substitution in the endogenous murine *Unc93b1* locus, resulting in amino acid substitution T93I. **(B)** Observed percentages of pups of the indicated genotypes surviving to weaning age; expected Mendelian percentages are indicated in parentheses. **(C)** Body weight of 5-wk-old mice. **(D)** Normalized spleen weight of 10-wk-old mice. **(E)** Representative flow cytometry plots, frequency among live splenocytes, and absolute number of splenic germinal center (GC) B cells. **(F and G)** Frequency and absolute number of splenic ABCs and plasma cells. **(H)** Representative flow cytometry plots, frequency among live splenocytes, and absolute number of splenic T-follicular helper (T_FH_) cells. **(I)** Frequency and absolute number of splenic Ly6C^lo^ monocytes. Data are pooled from five litters (B); eight litters (C); or two independent experiments (D and F–I) or are plotted from one and representative of two independent experiments (E). Symbols overlying bars represent values from individual mice (C–I). P values were determined using a one-way ANOVA with Sidak’s (C) or Dunnett’s (D–I) correction for multiple comparisons. **(J and K)** BMMs were prepared from mice of the indicated genotypes and stimulated overnight with PolyIC (20 µg ml^−1^), R848 (4 ng ml^−1^), ssPolyU complexed with DOTAP (6 µg ml^−1^), CpG-B (50 nM), or LPS (2 ng ml^−1^). Cytokine production was measured in supernatants by LEGENDPlex assay. Data are mean ± SD of triplicate technical replicates, representative of at least two independent experiments, and P values were determined using an unpaired two-tailed Student’s *t* test. *P ≤ 0.05, **P ≤ 0.01, ***P ≤ 0.001.

**Figure S5.**
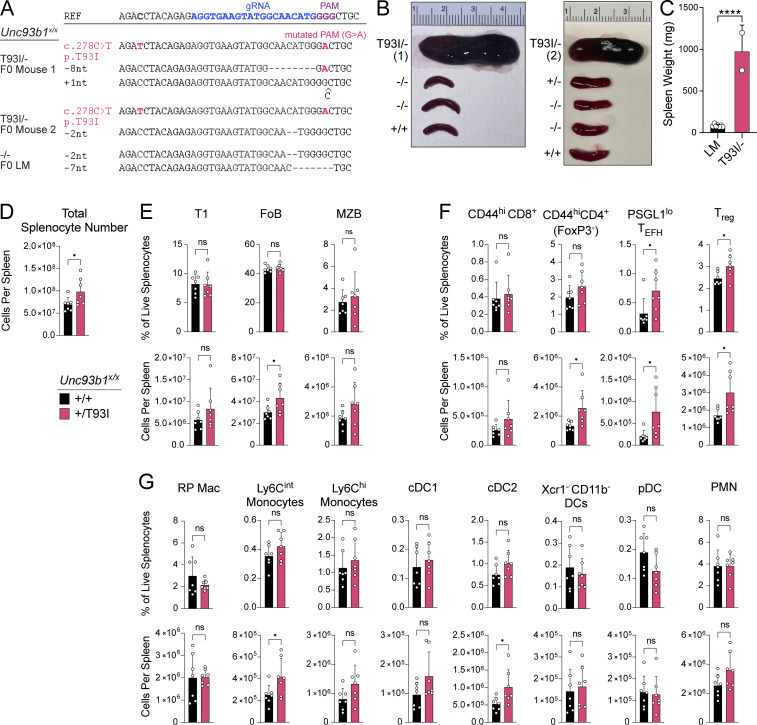
***Unc93b1***^***T93I***^ **knock-in mice develop severe systemic inflammation. (A)** Representative sequencing results for mice with confirmed hemizygous *Unc93b1*^*T93I/^−^*^ knock-in versus *Unc93b1*^*−/−*^ littermate. Founder (F0) mice were sequenced for the presence of indels or the desired c.278C>T, p.T93I genomic edit. Blue text indicates the gRNA sequence used during mouse generation; purple text indicates the PAM site. **(B and C)** Spleens from *Unc93b1*^*T93I/−*^ mice (*n* = 2) or their littermates (*n* = 7, LM) upon their death or sacrifice, respectively, at 8–10 wk. Quantification in C. “LM” indicates *Unc93b1*^*+/+*^, *Unc93b1*^*+/−*^, or *Unc93b1*^*−/−*^ littermate genotypes (i.e., lacking the correctly edited *Unc93b1*^*T93I*^ knockin allele). Data are mean ± SD, pooled from two independent experiments. P value determined by unpaired two-tailed Student’s *t* test. **(D)** Absolute splenocyte counts in *Unc93b1*^*T93I*^ mice. **(E–G)** Frequency and number of splenocyte subsets from *Unc93b1*^*T93I*^ mice: (E) B lineage cells, (F) T lineage cells, (G) myeloid cells. T1, transitional 1 B cells; FoB, follicular naive B cells; MZB, marginal zone B cells; T_EFH_, T-extrafollicular B helper cells; T_reg_, regulatory T cells; RP Mac, red pulp macrophages; cDC, conventional dendritic cells; pDC, plasmacytoid dendritic cells. In D–G, mean with SD are plotted, and symbols represent values from individual mice; P values were obtained using a one-way ANOVA. *P ≤ 0.05, ****P ≤ 0.0001.

We also generated *Unc93b1*^*T93I*^ knock-in mice by introducing a c.278, C>T substitution using Cas9 genome editing (Fig. 5 A). We initially obtained two founder mice (one male and one female), each with one correctly edited *Unc93b1*^*T93I*^ allele. Deep sequencing analysis revealed that the second *Unc93b1* allele in both mice contained deletions leading to frameshifts and premature termination, so these initial founder mice were effectively *Unc93b1*^*T93I/−*^ hemizygotes (Fig. S5 A). Both hemizygous founder mice died before breeding (the female died at 8 wk, the male at 10 wk), but we were able to perform gross necropsy, which revealed extreme splenomegaly in both *Unc93b1*^*T93I/-*^ mice relative to *Unc93b1*^*+/+*^, *Unc93b1*^*+/−*^, and *Unc93b1*^*−/−*^ littermates (Fig. S5, B and C). We were subsequently able to generate an *Unc93b1*^*+/T93I*^ female founder animal that we bred to C57BL/6J males to establish a line of *Unc93b1*^*+/T93I*^ mice. Analysis of genotypes from *Unc93b1*^*+/T93I*^ intercrosses revealed an absence of homozygous *Unc93b1*^*T93I/T93I*^ progeny (Fig. 5 B), indicating that homozygosity for the *Unc93b1*^*T93I*^ allele results in embryonic or perinatal lethality. Interestingly, unlike the *Unc93b1*^*+/R336C*^ female mice, *Unc93b1*^*+/T93I*^ female mice did not display a reduction in weight at 5 wk old, suggesting different pathologies between the two mutations (Fig. 5 C). Analysis of 10-wk-old *Unc93b1*^*+/T93I*^ animals revealed splenomegaly (Fig. 5 D), and within the B cell compartment, we observed increased numbers of germinal center B cells, ABCs, and plasma cells (Fig. 5, E–G). In addition, we observed an expansion of T-follicular helper cells and of non-classical Ly6C^lo^ splenic monocytes, which is consistent with systemic inflammation in these animals (Fig. 5, H and I). BMMs from *Unc93b1*^*+/T93I*^ mice showed heightened responses to TLR7 ligands and weakly increased responses to TLR3 ligands, while responses to TLR9 or TLR4 ligands were not affected (Fig. 5, J and K). These results indicate that the *Unc93b1*^*T93I*^ allele is sufficient to drive severe inflammatory disease in mice, even when heterozygous, and suggest that the disease is driven by increased TLR7 responses.

## Discussion

The work described here establishes that coding variants in *UNC93B1* that increase TLR responses to nucleic acids can drive cutaneous and systemic autoimmune disease in humans. Our mutagenesis screen across all cytosolic and luminally exposed residues has defined multiple regions of the UNC93B1 protein that when mutated alter responses by TLR3, TLR7, and TLR9, expanding our understanding of the key residues in UNC93B1 required to limit TLR responses to self-nucleic acids. The human variants we identified map to two distinct regions of the UNC93B1 protein: Thr93 on loop 1 on the luminal side of the membrane and Arg336 on loop 6 in the cytosol. Our previous work had implicated residues in loop 6 and the C-terminal tail as critical for limiting TLR7 responses (Majer et al., 2019a); however, the specific residues identified in our current work were not examined. Indeed, our mutagenesis screen identified a number of additional residues within each of these regions as well as other regions in UNC93B1 (Fig. 1 C) that regulate TLR3, TLR7, and/or TLR9. As whole-exome and whole-genome sequencing of patients becomes more standard, coding variants mapping to these regions will likely be identified. To that end, other groups have recently described two additional coding variants in *UNC93B1* that map to regions identified as regulatory for TLR7 by our screen: UNC93B1^E49dup^ in the N-terminus, UNC93B1^E92G^ in loop 1, adjacent to the UNC93B1^T93I^ variant that we describe here, and a different missense variant at Arg336, UNC93B1^R336L^ (Mishra et al., 2024; Wolf et al., 2024).

While a full characterization of the mechanisms underlying the phenotypes caused by each variant identified here is beyond the scope of this manuscript, our initial analyses support the emerging view that UNC93B1 regulation of TLR function is complex and multifaceted. The UNC93B1^T93I^ variant weakens the interaction between UNC93B1 and TLR7. Precisely how this reduced interaction increases TLR7 responses remains to be determined. Notably, our work disfavors the model that UNC93B1-released molecules of TLR7 in endosomes evade UNC93B1-mediated negative regulation. Instead, we hypothesized that UNC93B1^T93I^ abrogates a critical interaction between the ectodomain of TLR7 and loop 1 of UNC93B1, conferring greater sensitivity for ligand detection. The possibility that such steric inhibition could limit ligand binding by TLR7 when associated with UNC93B1 was originally proposed by Ishida and colleagues when they first reported the TLR7-UNC93B1 structure (Ishida et al., 2021), and our characterization of UNC93B1^T93I^ variant is consistent with this model. One counterintuitive aspect of this model, though, is that UNC93B1 is required for the export of TLRs from the ER. We observed less endosomal TLR7 in UNC93B1^T93I^-expressing cells (Fig. 3 F), suggesting that the weakened interaction does affect ER export. The reduced amount of TLR7 is presumably sufficiently hypermorphic to overcome the reduction in receptor levels in endosomes. Alternatively, although we think this possibility unlikely, ER-localized TLR7 may retain some ability to signal, as has been suggested by others (Pelka et al., 2018), and this activity may be enhanced by a weakened interaction with UNC93B1. Intriguingly, characterization of the UNC93B1^R336C^ mutant revealed an increase in total and K63-linked ubiquitylation and SYNTENIN-1 association, both of which have previously been implicated in limiting TLR7 signaling (Majer et al., 2019a). While this result may suggest that the UNC93B1^R336C^ mutant alters interaction with yet-to-be-identified downstream machinery, it is also possible that the mutation impacts other aspects of UNC93B1-mediated regulation. For example, the UNC93B1^R336C^ variant may destabilize the UNC93B1:UNC93B1 dimer in a way that enhances TLR7 signaling, as the two loop 6 helices interact at the dimer interface (Fig. 3 B). The functional relevance of the distinct stoichiometry of the TLR7-UNC93B1 complex remains unclear and is an important area for future investigation.

An important implication of this work is that heterozygosity for certain coding variants of *UNC93B1* is sufficient to drive autoimmune disease in mice and humans. Both of the variants that we identified, *UNC93B1*^*T93I*^ and *UNC93B1*^*R336C*^, are heterozygous in the affected probands, and our analysis of *Unc93b1*^*+/R336C*^ and *Unc93b1*^*+/T93I*^ mice supports the conclusion that one allele of these variants is sufficient to cause autoimmune pathology. The absence of ANA in proband B.III.1 notwithstanding, the confluence of inflammatory arthritis, tumid/chillblain lupus rashes, neuroinflammatory lesions reminiscent of Aicardi-Goutieres Syndrome, peripheral blood mononuclear cell surface phenotype suggesting elevated serum IFN-I activity, and improvement with baricitinib (JAK1/JAK2 inhibitor) therapy all point toward an interferonopathy with autoimmune features. Indeed, the characterization of her *UNC93B1* mutation may prompt additional autoantibody testing and clinical monitoring for lupus complications such as nephritis. The presence of classic anti-dsDNA-antibody^+^ SLE with nephritis in the probands of recent studies by Mishra et al. and Wolf et al. harboring *UNC93B1*^*+/E49dup*^, *UNC93B1*^*E92G/E92G*^, and *UNC93B1*^*+/R336L*^ mutations further support the idea that SLE is a common phenotypic outcome of *UNC93B1* mutations that disrupt negative regulation of TLR7 and is also consistent with the observation of systemic lupus in patients with gain-of-function mutations in TLR7 (Brown et al., 2022; Mishra et al., 2024; Wolf et al., 2024). The distinct clinical phenotype of our *UNC93B1*^*+/R336C*^ proband may hint at variable expressivity of TLR-activating *UNC93B1* mutations, possibly underpinned by additional genetic or environmental factors. Future work will address whether *Unc93b1*^*R336C*^ mice recapitulate the neuroinflammation present in proband B.III.1.

It is also noteworthy that proband A.III.1 in our study with a heterozygous *UNC93B1*^*+/T93I*^ mutation developed early-onset isolated cutaneous tumid lupus (as did her two siblings with ANA seropositivity, and her father), whereas both *UNC93B1*^*E92G/E92G*^ siblings described by Wolf et al. (2024) developed systemic lupus with nephritis and several other organ-threatening manifestations. We suspect that this discrepancy in disease expression may be underpinned by allelic homozygosity for *UNC93B1*^*E92G/E92G*^ in the study from Wolf et al. (2024) versus heterozygosity for *UNC93B1*^*+/T93I*^ in the kindred described here. Interestingly, residue E92 was captured in our UNC93B1-scanning mutagenesis screen in RAW264.7 macrophages and in a mutant bearing the triple-alanine substitution UNC93B1^92-ETY/AAA-94^. This mutant was shown to have strong hypermorphic cytokine responses even in the absence of exogenous ligand addition (Fig. 1 C and Fig. 2 F), whereas *Unc93b1*^*−/−*^ RAW cells transduced to express the human UNC93B1^T93I^ variant showed highly elevated TLR3/7/9-ligand-inducible activity but comparatively modest elevated basal cytokine production (Fig. S2 E). The E92G variant characterized by Wolf et al. (2024) also enhanced TLR7-ligand-inducible but not basal cytokine production in RAW macrophages. Altogether, these observations suggest that both E92 and T93 contribute to the negative regulation of TLR7 signaling by UNC93B1, and further genetic analysis of humans and knock-in mice may clarify the impact of homozygous versus heterozygous allelic states on disease expression.

Finally, we note that the number and effect size of UNC93B1 residues that negatively regulate endosomal TLR signaling are greater for TLR7 than for TLR3 or TLR9 (Fig. 1, C and D), and all characterized proband mutations in our study enhance TLR7/8 responses more drastically than TLR3 or TLR9 responses (Fig. 2, C–E and Fig. S2 E). This may partially explain the convergence of clinical phenotypes in *UNC93B1* mutant patients on systemic and/or cutaneous lupus disease, given the strong evidence for *Tlr7* gene dosage in the severity of murine lupus, and more recent reports of gain-of-function human *TLR7* allelic variants associated with SLE (Brown et al., 2022). Nevertheless, given mild enhancement of TLR3 and/or TLR9 responses in UNC93B1^T93I^ and UNC93B1^R336C^ RAW cells (Fig. 2 E and Fig. S2 E), it will be important to ascertain whether the disease in *Unc93b1*^*T93I*^ mice and *Unc93b1*^*R336C*^ mice is fully TLR7-dependent, or whether TLR3 or TLR9 also plays a role.

In summary, our study maps the impact of specific UNC93B1 residues on TLR3, TLR7, and TLR9 responses and describes novel human coding variants that enhance TLR7 signaling in leukocytes and that cause autoimmune pathology in mice and humans. We hope that the regulatory landscape elucidated herein will provide a resource for clinicians and scientists faced with emerging genome and exome data that may reveal additional, yet-to-be-reported *UNC93B1* coding variants. Moreover, our findings suggest that even heterozygous gene variants that enhance TLR7 signaling may be sufficient to cause autoimmune disease, underscoring the importance of studies to define the prevalence of *UNC93B1* mutations in patients with systemic autoimmunity and of systematically identifying additional negative regulators of this pathway.

### Limitations of the study

While our mutagenesis screen covered all tail and loop residues, we did not probe the function of residues within transmembrane regions of UNC93B1. Some of these residues contribute to UNC93B1-TLR interactions and may influence TLR function. Our screen also did not address how mutations impact TLR8 function because TLR8 is not functional in RAW macrophages. Moreover, we have not formally demonstrated that the disease observed in *Unc93b1*^*T93I*^ mice and *Unc93b1*^*R336C*^ mice requires TLR7 function. While we believe TLR7 dependence is likely, based on our signaling analyses, it remains possible that other TLRs contribute to the disease observed in these mice.

## Materials and methods

### UNC93B1 mutagenesis screen

A library of 92 single-, double-, or triple-alanine mutations along the tail and loop regions of UNC93B1 was synthesized and provided as individual plasmids by Invitrogen. The mouse *Unc93b1* gene was tagged with a C-terminal 3× Flag (DYKDHDGDYKDHDIDYKDDDDK) and was codon-optimized for *Mus musculus*, with regions of high (>80%) and low (<30%) GC content avoided. *Unc93b1* mutants were cloned into a murine stem cell virus (MSCV) retroviral vector carrying an IRES-driven PuromycinR-T2A-mCherry double-selection cassette (MSCV-Puro-mCherry). Retrovirus encoding each *Unc93b1* mutant was generated and overlaid onto an *Unc93b1*^*−/−*^ RAW264.7 macrophage cell line (Majer et al., 2019a) to ultimately generate stable mutant cell lines (described in detail below).

For the screen, we stimulated each RAW264.7 mutant line in triplicate with ligands for TLR3 (Poly(I:C) HMW 20 µg/ml), TLR7 (R848 10 ng/ml), TLR9 (CpG-B 45 nM), and TLR4 (LPS 5 ng/ml). Ligand doses were carefully determined by titration experiments to identify a dose that yielded a robust but sub-maximal response, thus providing a dynamic range to reveal increased and decreased responses. TNF production was measured by ICS (described below). The 92 mutants were tested in multiple batches, with each batch containing UNC93B1^WT^ and non-functional UNC93B1^H412R^, as well as empty MSCV-Puro-mCherry vector controls, for normalization between batches. The average geometric mean fluorescence intensity (gMFI) of the triplicates of each mutant under each stimulation condition was calculated and divided by the average gMFI of the matched UNC93B1^WT^ control per batch to determine fold-change (FC) values. For Fig. 1, all FC values were log_2_-transformed and plotted as a heatmap to illustrate the relative changes in TLR signaling intensity. See Data S1 for raw data.

### Cell lines and cell culture

THP-1 cells were a gift from Russell Vance (University of California, Berkeley [UC Berkeley], Berkeley, CA, USA) and were cultured in RPMI 1640 with 10% (vol/vol) fetal calf serum (FCS), *L*-glutamine, and penicillin–streptomycin (Gibco), at a concentration between 10^5^ and 10^6^ cells/ml. Prior to stimulation with TLR ligands, THP-1 cells were differentiated from monocytes to macrophages as follows. On day 0, 50,000 THP-1 cells were seeded per well of a tissue culture–treated flat-bottom 96-well plate, with 100 ng/ml phorbol myristate acetate (PMA; Sigma-Aldrich) added to the culture media. On day 3, PMA-containing media was removed, and cells were rested in normal culture media for an additional 3 days. On day 6, culture media was refreshed, and cells were stimulated with TLR ligands on day 7. THP-1s were stimulated with R848, TL8-506, or Pam3CSK4 (all from Invivogen). For measuring production of cytokines after stimulation, cell-free supernatants were harvested after overnight (22 h) incubation at 37°C, 5% CO_2_. Cytokine concentrations in supernatants were quantified using the LEGENDplex Human Inflammation Panel 1 (740808; BioLegend) according to the manufacturer’s instructions (except that recommended reagent volumes were halved) using a BD LSR Fortessa Analyzer.

UNC93B1-deficient THP-1 cells were generated as follows. In brief, guide RNAs (gRNAs) specific for *UNC93B1* were designed using ChopChop (Labun et al., 2019); gRNA target site: 5′-TAC​GAC​GAG​ACC​TAC​CGC​GAG​G-3′ (PAM highlighted in bold). THP-1 cells were electroporated (Biorad GenePulser) with a U6-sgRNACMV-mCherry-T2A-Cas9 plasmid generated by ligation-independent cloning to contain the *UNC93B1*-specific gRNA (Schmidt et al., 2015). Cells were sorted on a BD FACSAria Fusion cell sorter for mCherry-positive cells that were then cloned by limiting dilution. Monoclonal cell lines were cultivated and genotyped using deep sequencing as previously described (Schmidt et al., 2016). Selected knockout cell clones contained all-allelic frameshift mutations without any wild-type reads. Additionally, functional knockout of UNC93B1 was confirmed by stimulation of TLR8 with R848 and TL8-506 compared with control stimulation of TLRs TLR2/TLR1 with Pam3CSK4, TLR2/TLR6 with Pam2CSK4, and TLR4 with LPS-EB Ultrapure (data not shown). In our studies, three independent *UNC93B1*^*−/−*^ single-cell clones were analyzed; one representative knockout clone (D6) is shown in the main text, with additional knockout clones D9 and E5 shown in Fig. S2.

GP2-293 packaging cell lines (Clontech) or RAW264.7 macrophage cell lines (ATCC) were cultured in DMEM or RPMI 1640, respectively, with 10% (vol/vol) FCS, *L*-glutamine, penicillin-streptomycin, sodium pyruvate, and HEPES (pH 7.2–7.5) (Gibco). *Unc93b1*^*−/−*^ RAW264.7 macrophages were generated as described previously (Majer et al., 2019a). *Unc93b1*^*−/−*^ RAW264.7 macrophages were stably transduced to express codon-optimized murine TLR3-HA and TLR7-HA from an MSCV-Thy1.1 plasmid. Murine BMMs were differentiated for 6–7 days in DMEM complete media (same supplements as above plus 50 µM β-mercaptoethanol) and supplemented with 10% (vol/vol) M-CSF containing supernatant from 3T3-CSF cells. For testing of TLR responses in RAW264.7 macrophage or BMM lines, 25,000 or 50,000 cells were seeded into tissue culture–treated or non-tissue culture–treated flat-bottom 96-well plates, respectively. The next day, cells were stimulated with the indicated TLR ligands: TLR3 (Poly(I:C) HMW), TLR4 (LPS-EB Ultrapure), TLR7 (R848 or ssPoly(U) Naked), TLR9 (CpG-B ODN 1668), all from Invivogen. ssPoly(U) Naked was complexed with DOTAP Liposomal Transfection Reagent (11202375001; Roche), following instructions given by Miltenyi Biotec. To measure secreted cytokines, cell-free supernatants were harvested after 8 h or overnight (16 h) incubation of RAW264.7 or BMM lines, respectively, at 37°C, 5% CO_2_. Cell supernatants were analyzed using the LEGENDplex Mouse Inflammation Panel (740150; BioLegend), according to the manufacturer’s instructions (except that recommended reagent volumes were halved), using a BD LSR Fortessa Analyzer.

For ICS of RAW264.7 lines or BMM lines, 45,000 or 50,000 cells were seeded into tissue culture-treated or non-tissue culture-treated flat-bottom 96-well plates, respectively. The next day, cells were stimulated and incubated with the indicated TLR ligands for 30 min at 37°C, 5% CO_2_, whereupon BD GolgiPlug Protein Transport Inhibitor (cat. 555029) was added at a 1:1,000 final dilution, followed by an additional 5.5 h of incubation at 37°C, 5% CO_2_ (6 h total stimulation time). Staining of dead cells and blocking of Fcγ receptors was performed by incubation with LIVE/DEAD Fixable Violet Dead Cell Stain (L34955; Invitrogen) and anti-CD16/32 (clone 2.4G2) in plain PBS for 20 min at 4°C. For BMMs only, surface staining was performed in PBS with 2% calf serum, 2 mM EDTA, and 0.05% sodium azide for 20 min at 4°C. Intracellular staining of TNF (and additionally IL-12p40 for BMMs) was performed using the BD Cytofix/CytopermPlus Fixation/Permeabilization Solution Kit (cat. 555028) according to the manufacturer’s instructions with 30 min incubation at 4°C. Antibodies used for flow cytometry are listed in Table S1. Flow cytometry was performed using a BD LSR Fortessa Analyzer.

Validation of the SYNTENIN-1 antibody used for immunoblotting was performed in Hoxb8-Macrophages endogenously expressing Cas9 and transduced with gRNAs targeting SYNTENIN-1 in the lentiGuide-Puro vector (Plasmid #52963; Addgene), following methods previously described (Roberts et al., 2019).

### Generation of cell lines with mouse and human UNC93B1 variants

Human *UNC93B1* (NM_030930.4) with a C-terminal 3× Flag (DYKDHDGDYKDHDIDYKDDDDK) was synthesized by Invitrogen’s GeneArt Gene Synthesis service (no codon-optimization). Wild-type and R336C UNC93B1 variants were individually synthesized, while the T93I and PRP/AAA variants were cloned using the NEBuilder HiFi DNA Assembly Cloning Kit (E5520S; NEB) from the wild-type sequence. *UNC93B1* mutants were then cloned into the MSCV-Puro-mCherry retroviral vector.

Retroviral transduction and generation of stable RAW264.7 macrophage or THP-1 cell lines were performed as follows. Lipofectamine2000 Transfection Reagent (Invitrogen) and Opti-MEMI (Gibco) were used for transfection of plasmid DNA into the GP2-293 packaging line, with 0.8 µg pVSV-G (8454; Addgene), 1.7 µg MSCV-Puro-mCherry plasmid of interest, and 5 µl Lipofectamine2000 used per well of a 6-well tissue culture–treated plate. For generation of the mouse UNC93B1 scanning–alanine mutagenesis library, Lipofectamine-LTX Transfection Reagent (6.25 µl; Invitrogen) was used instead of Lipofectamine2000. 16 h after transfection, the medium was replaced with complete DMEM. After 24–36 h, viral supernatants were collected, filtered through a 0.45-µm filter, and overlaid onto target cells with the addition of polybrene (20 µg/ml for RAW264.7 lines and 8 µg/ml for THP-1 lines) to enhance transduction efficiency. THP-1 cells were additionally spinfected at 1,000 *g* for 2 h at 32°C. Cells were cultured for 48 h after transduction, at which point puromycin dihydrochloride selection (A1113803; Thermo Fisher Scientific) at 4.5 µg/ml for RAW264.7 lines or 4 µg/ml for THP-1 lines was initiated. The efficiency of drug selection was verified by mCherry expression of target cells on a BD LSR Fortessa Analyzer. For biochemistry experiments, target cells were sorted on a Becton Dickinson Aria Fusion Sorter to match UNC93B1 expression levels using the bicistronic fluorescent mCherry reporter.

### Immunoprecipitation and western blot

For whole-cell lysate analysis, cells were lysed in PS6K Lysis Buffer (1% Triton X-100, 4 mM EDTA, 40 mM HEPES, 10 mM sodium pyrophosphate, 10 mM sodium β-glycerophosphate, 1 mM sodium orthovanadate, 10 mM sodium fluoride, pH 7.4). For co-immunoprecipitations, cells were lysed in a CHAPS lysis buffer (0.5% CHAPS, 4 mM EDTA, 10% glycerol, 40 mM HEPES, 150 mM NaCl, 10 mM sodium pyrophosphate, 10 mM sodium β-glycerophosphate, 1 mM sodium orthovanadate, 10 mM sodium fluoride, pH 7.6) freshly supplemented with 20 mM N-ethylmaleimide. For ubiquitylation analyses, cells were lysed in radioimmunoprecipitation assay (RIPA) buffer (1% NP-40, 0.1% SDS, 0.5% sodium deoxycholate, 50 mM Tris, 150 mM NaCl, pH 7.4) freshly supplemented with 40 mM N-ethylmaleimide. All lysis buffers were freshly supplemented with 1 mM PMSF and protease inhibitor (Pierce) immediately prior to use. Cells were incubated at 4°C for 1 h with end-over-end inversion to facilitate lysis and clarified by centrifugation at 13,300 *g* for 15 min. All cell lysates were normalized by bicinchoninic acid assay** (**BCA) to ensure an equal amount of protein was used for downstream applications such as western blot or immunoprecipitation. For immunoprecipitations, lysates were incubated with anti-Flag (M8823; Sigma-Aldrich) magnetic resin (pre-blocked with 1% BSA-PBS) for >2 h and washed three times in lysis buffer. For ubiquitylation analyses, the captured fractions were washed in a high salt wash buffer (RIPA with an additional 350 mM NaCl) five times, with each wash carried out for 5 min at 4°C with agitation. Precipitated proteins were eluted in lysis buffer containing 150 µg/ml of 3xFlag peptide (F4799; Sigma-Aldrich) or denatured in Laemmli Sample buffer (Bio-Rad) at RT for 1 h. Proteins were separated by SDS-PAGE (Genscript SurePAGE) and transferred to Immobilon PVDF membranes (Millipore) in an OWL VEP-2 electroblotting system (Thermo Fisher Scientific). Membranes were blocked in 5% BSA made up of Tris-buffered saline with 0.2% Tween 20 (TBS-T) before an overnight incubation with primary antibody at 4°C in 5% BSA. The membranes were washed in TBS-T and developed after an incubation of 1 h in species-specific horseradish peroxidase–conjugated secondary antibody (Vector Laboratories), visualized using SuperSignal west pico chemiluminescent substrate (Thermo Fisher Scientific), and imaged using the ChemiDoc XRS+ system (Bio-Rad). The antibodies used for immunoblotting are listed in Table S2.

### Phagosome enrichment

Cells in confluent 15-cm dishes were incubated with ∼10^8^ 1 µm magnetic beads (Polysciences) for 4 h. Cells were washed 3× with ice-cold PBS, scraped in 10 ml sucrose homogenization buffer (SHB), and pelleted by centrifugation. Cells were resuspended in 2 ml SHB with freshly supplemented protease inhibitor cocktail (Pierce) and 1 mM PMSF and mechanically lysed. Lysed cells were gently rocked on ice to free endosomes for 10 min. Beads were collected with a magnetic rack and washed five times with SHB plus protease inhibitor. After the final wash, phagosome preparations were lysed in either PS6K lysis buffer or CHAPS lysis buffer for 1 h on ice. Eluted proteins were then quantified by BCA and a minimum of 2 µg of lysate was loaded per lane for western blot analysis.

### Cellular analysis of human variant mice

Single-cell splenocyte suspensions were prepared by mechanical dissociation of spleen tissue over 100-µm cell strainers (Corning) and subsequent ammonium-chloride potassium lysis (Gibco) to remove red blood cells. For the analysis of splenic myeloid cell populations, spleen tissue was minced and digested in 0.07 mg/ml LiberaseTM (729904; Roche) and 0.05 mg/ml DNAseI (D4513; Sigma-Aldrich) prior to 100-µm filtration and ACK lysis. Staining of dead cells and blocking of Fcγ receptors was performed by incubation with LIVE/DEAD Fixable Aqua Dead Cell Stain (L34957; Invitrogen) and anti-CD16/32 (clone 2.4G2) in plain PBS for 45 min on ice. Surface staining was performed in PBS with 2% calf serum, 2 mM EDTA, and 0.05% sodium azide for 30 min at 21°C. Intracellular staining of Tbet, FoxP3, and Bcl6 was performed using the eBioscience fixation and permeabilization kit (cat. 00-5523-00) according to the manufacturer’s instructions with a 20-h incubation on ice. Antibodies used for flow cytometry are listed in Table S1. Flow cytometry was performed using a BD Symphony A3 Analyzer, a BD LSR Fortessa Analyzer, or a BD LSR Fortessa X-20 Analyzer.

### ANA ELISA

ANA ELISA plates were purchased from Werfen/Inova Diagnostics (cat. 708750, detects IgG to native, recombinant extracts from HEp-2 nuclei and nucleoli), and ELISAs for ANA were performed according to the manufacturer’s instructions with the following modifications: (1) goat-anti-mouse IgG (H+L)-biotin and streptavidin-HRP (1031-08, 7105-05; Southern Biotech) were used in place of goat-anti-human IgG-HRP provided in the test kit and (2) sera pooled from TLR7.1 transgenic mice (Deane et al., 2007) and muMt^−/−^ mice were used as positive and negative controls, respectively. The OD450 of TLR7.1 serum at a dilution of 1:40 was assigned an arbitrary value of 100, and the TLR7.1-normalized ODs of each 1:40-diluted experimental sample were multiplied by 100 to yield the plotted values. Sera from TLR7.1 transgenic mice was a kind gift from the lab of Dr. Jessica Hamerman (Benaroya Research Institute, Seattle, WA, USA).

### Mice

All mouse experiments were performed in accordance with the guidelines of the Animal Care and Use Committee at UC Berkeley. Mice were housed under specific pathogen–free conditions at UC Berkeley. C57BL/6J (strain 000664) and µMt^−/−^ (strain 002288) mice were purchased from the Jackson Laboratory.

*Unc93b1*^*R336C*^ and *Unc93b1*^*T93I*^ mice were generated using Cas9 genome editing by the UC Berkeley Cancer Research Laboratory Gene Targeting Facility as previously described (Majer et al., 2019a). For *Unc93b1*^*R336C*^ mice, the following gRNA was used: 5′-AGA​TAA​AGA​AGG​GCA​CCA​GA**TGG**-3′ (PAM highlighted in bold). The single-stranded oligonucleotide repair template, containing a 99-bp left homology arm and a 66-bp right homology arm, was synthesized as a 4-nmole UltramerDNA Oligo by Integrated DNA Technologies. The following edits were introduced: 5′-**T**GTG​ACT​TTC​GCT​TAC​G**G**-3′ compared with the wild-type 5′-CGT​GAC​TTT​CGC​TTA​CGC-3′ sequence; the first mutation introduces the desired C>T, p.R336C mutation, while the second mutation is a silent mutation of the PAM site to prevent recutting after template-mediated repair. Offsprings were genotyped by Sanger Sequencing for the presence of the correctly edited *Unc93b1* allele and further backcrossed to C57BL/6J mice for at least two generations to ensure germline transmission. Two independent *Unc93b1*^*R336C*^ mouse lines were generated from two independent founder (F0) mice; both mouse lines are represented as pooled data in the figures.

For *Unc93b1*^*T93I*^ mice, the following gRNA was used: 5′-AGG​TGA​AGT​ATG​GCA​ACA​TG**GGG**-3′ (PAM highlighted in bold). The single-stranded oligonucleotide repair template, containing an 85-bp left homology arm and a 68-bp right homology arm, was synthesized as a 4-nmole UltramerDNA Oligo by Integrated DNA Technologies. The following edits were introduced: 5′-**T**CTA​CAG​AGA​GGT​GAA​GTA​TGG​CAA​CAT​GGG**A**-3′, compared with the wild-type 5′-CCT​ACA​GAG​AGG​TGA​AGT​ATG​GCA​ACA​TGG​GG-3′ sequence; the first mutation introduces the desired C>T, p.T93I mutation, while the second mutation is a silent mutation of the PAM site to prevent recutting after template-mediated repair. Founder mice were initially genotyped by Sanger sequencing for the presence of the correct knock-in allele, although both *Unc93b1*^*T93I*^ founders died before breeding and establishment of the line could be achieved. Upon their death, founder mice and their littermates were further genotyped by adapting deep sequencing protocols described for the generation of monoclonal cell lines (Schmidt et al., 2016). In brief, the knock-in-containing genomic region was PCR-amplified from genomic DNA using the primers: Fwd: 5′-ACA​CTC​TTT​CCC​TAC​ACG​ACG​CTC​TTC​CGA​TCT​GGT​CGC​TTA​GGT​GAC​TCG​G-3′, Rvs: 5′-TGA​CTG​GAG​TTC​AGA​CGT​GTG​CTC​TTC​CGA​TCT​GCG​GAA​ACA​GAA​GTT​TGG​CA-3′. Illumina handles and barcodes were added, and the products were sequenced on an Illumina Miseq 250SR V2, with data analysis performed using Outknocker33 (Schmid-Burgk et al., 2014).

### Structural modeling

Structure figures were generated using ChimeraX (Pettersen et al., 2004). Thr93 and Arg336 in UNC93B1 are depicted in their highest probability rotamers, which are consistent with the formation of hydrogen bond contacts with TLR7 and UNC93B1, respectively.

### Whole-exome sequencing and variant identification

Exome sequencing from Proband A.III.1 (*UNC93B1*^*+/T93I*^) was performed at Children’s Mercy Hospital Kansas City after written informed consent and a publication agreement statement were obtained from all participants and parents. All studies were approved by the Children’s Mercy Hospital Institutional Review Board (study #11120514). Genomic DNA from the submitted sample was prepared using standard extraction methods and then subjected to Kapa (Roche) or TruSeq (Illumina) library prep kits. Samples were enriched using IDT xGenv2 exome research panel supplemented with custom mitochondrial probes and sequenced to a minimum of 7 Gb for a mean of 80× average coverage or greater on an Illumina NovaSeq 6000 (2 × 150 paired-end reads) or Illumina NextSeq 2000 (2 × 150 paired-end reads). Bidirectional sequences were assembled, aligned to reference gene sequences based on human genome build GRCh38/UCSC hg38, and analyzed using Emedgene software. The variant interpretation was performed using the 2015 ACMG Standards and Guidelines for the Interpretation of Sequence Variants (Richards et al., 2015) with a systematic medical literature review.

Genome sequencing for Proband B.III.1 (*UNC93B1*^*+/R336C*^) was performed by Baylor Genetics, through the Undiagnosed Diseases Network (UDN), using previously described methods (Splinter et al., 2018) and with approval from the National Human Genome Research Institute’s central institutional review board (registration number 00000014). Informed consent for testing and publication of clinical information was obtained from the proband and her parents under protocol 15-HG-0130 approved by the National Human Genome Research Institute. Libraries were prepared using a PCR-free 550-bp insert size protocol by the KAPA Hyper Prep kit (Roche). Sequencing was performed using the Illumina NovaSeq 6000 sequencing platform for 150 bp paired-end reads. FastQ data were aligned to the human reference genome build GRCh38 using the Illumina Dragen BioIT Platform. Variant calling was performed using the Illumina Dragen haplotype-based variant calling system. Analysis of the variant call sets at the UDN Stanford clinical site led to the identification of the *UNC93B1* variant in the proband. Sanger sequencing at Baylor Genetics was performed to confirm the *UNC93B1* variant in the proband and to assess segregation in the parents.

### Mouse renal pathology

Analysis of formalin-fixed, paraffin-embedded murine kidneys stained with hematoxylin/eosin, periodic acid-Schiff, or Jones methenamine silver was performed by the UC Davis Comparative Pathology Lab, with blinded scoring by a veterinary pathologist using a previously reported approach (Xia et al., 2015).

### Statistical analysis

Spearman’s rank correlation coefficients were calculated using Excel. All other statistical analyses were performed using GraphPad Prism 8 or GraphPad Prism 10 (GraphPad).

### Online supplemental material

Fig. S1 shows a scanning-alanine mutagenesis screen that reveals distinct domains of UNC93B1 that regulate endosomal TLR signaling. Fig. S2 shows human UNC93B1 variants enhance endosomal TLR signaling. Fig. S3 shows human UNC93B1 variants disrupt known negative regulatory mechanisms. Fig. S4 shows additional phenotyping of *Unc93b1*^*R336C*^ mice and gating strategies used to subset splenic leukocytes. Fig. S5 shows *Unc93b1*^*T93I*^ knock-in mice develop severe systemic inflammation. Table S1 shows antibodies used for flow cytometry. Table S2 shows antibodies used for immunoblotting. Table S3 shows histologic scoring of glomerulonephritis. Data S1 shows Log_2_FC of all stimulation conditions per mutant, normalized to WT (for Fig. 1 C). Data S2 shows proband clinical histories.

## Supplementary Material

Table S1 shows antibodies used for flow cytometry.

Table S2 shows antibodies used for immunoblotting.

Table S3 shows histologic scoring of glomerulonephritis.

Data S1 shows Log_2_FC of all stimulation conditions per mutant, normalized to WT (for Fig. 1 C).

Data S2 describes proband clinical histories.

SourceData F2 contains original blots for Fig. 2.

SourceData F3 contains original blots for Fig. 3.

SourceData FS1 contains original blots for Fig. S1.

SourceData FS3 contains original blots for Fig. S3.

## Data Availability

Patient sequencing results are available at dbGAP, accession phs001232.v5.p2. All other data are available in the main text or the supplementary materials. All materials (cell lines, mice, and plasmids) are available upon request and after completion of a material transfer request to the corresponding author (G.M. Barton).
