# Supplementary material for: Large-scale mutational analysis identifies UNC93B1 variants that drive TLR-mediated autoimmunity in mice and humans
Source: J Exp Med. 2024 May 23;221(8):e20232005. doi: 10.1084/jem.20232005 (PMC11116816; doi:10.1084/jem.20232005)
Supplement: Table S1 — shows antibodies used for flow cytometry. [file JEM_20232005_TableS1.docx]

**Table S1. Antibodies used for flow cytometry**

| **Antigenic Target** | **Fluorophore Conjugate** | **Clone** | **Vendor** | **Catalog Number** |
| --- | --- | --- | --- | --- |
| B220 | APC eFluor780 | RA3-6B2 | Invitrogen | 47-0452-82 |
| B220 | BV650 | RA3-6B2 | BioLegend | 103241 |
| Bcl6 | BV421 | K112-91 | BD | 563363 |
| CD11b | PerCP-eFluor710 | M1/70 | Invitrogen | 46-0112-82 |
| CD11c | FITC | N418 | Invitrogen | 11-0114-85 |
| CD11c | PE | N418 | Invitrogen | 12-0114-82 |
| CD138 | PE | 281-2 | BioLegend | 142504 |
| CD19 | BUV395 | 1D3 | BD | 563557 |
| CD21 | PacBlue | 7.00E+09 | BioLegend | 123414 |
| CD23 | APC | B3B4 | BioLegend | 101620 |
| CD4 | PerCP-Cy5.5 | GK1.5 | Tonbo | 65-0041-U100 |
| CD44 | BV785 | IM7 | BioLegend | 103059 |
| CD62L | PEDazzle | MEL-14 | BioLegend | 104447 |
| CD8a | APC eFluor780 | 53-6.7 | Invitrogen | 47-0081-82 |
| CD90.2 | BUV395 | 53-2.1 | BD | 565257 |
| CD95/Fas | PE-CF594 | Jo2 | BD | 741968 |
| CXCR3 | PE | CXCR3-173 | Invitrogen | 12-1831-82 |
| CXCR5 | BV650 | L138D7 | BioLegend | 145517 |
| F4/80 | PE-Dazzle594 | BM8 | BioLegend | 123146 |
| FoxP3 | AF488 | FJK-16s | Invitrogen | 53-5773-82 |
| GL7 | PerCP-Cy5.5 | GL7 | BioLegend | 144609 |
| Ly6C | BV785 | HK1.4 | BioLegend | 128041 |
| Ly6D | FITC | 49-H4 | BioLegend | 138606 |
| Ly6G | BUV395 | 1A8 | BD | 565964 |
| MHCII (I-A/I-E) | APC eFluor780 | M5/114.15.2 | Invitrogen | 47-5321-82 |
| NK1.1 | BUV395 | PK136 | Invitrogen | 363-5941-82 |
| PD-1 | PE-Cy7 | J43 | Invitrogen | 25-9985-82 |
| PDCA-1 | PE-Cy7 | eBio927 | Invitrogen | 25-3172-82 |
| PSGL-1 | BUV395 | 2PH1 | BD | 740273 |
| SiglecH | APC | eBio440c | Invitrogen | 17-0333-82 |
| Tbet | PE-Cy7 | 4B10 | BioLegend | 644824 |
| TCRβ | APC | H57-597 | BioLegend | 109212 |
| TLR7 | PE | A94B10 | BD | 565557 |
| Xcr1 | BV421 | ZET | BioLegend | 148216 |
| TNF | APC | MP6-XT22 | Invitrogen | 17-7321-82 |
| IL-12p40 | PE | C17.8 | Invitrogen | 12-7123-82 |
| CD11b | BV785 | M1/70 | BioLegend | 101243 |
| F4/80 | PE-Cy7 | BM8 | BioLegend | 123114 |
| Ly6G | APC-Cy7 | 1A8 | BD Biosciences | 560600 |
| Ly6C | PerCP/Cyanine5.5 | HK1.4 | BioLegend | 128012 |
