# Supplementary material for: Large-scale mutational analysis identifies UNC93B1 variants that drive TLR-mediated autoimmunity in mice and humans
Source: J Exp Med. 2024 May 23;221(8):e20232005. doi: 10.1084/jem.20232005 (PMC11116816; doi:10.1084/jem.20232005)
Supplement: Table S2 — shows antibodies used for immunoblotting. [file JEM_20232005_TableS2.docx]

**Table S2. Antibodies used for immunoblotting**

| **Antigenic target** | **Target species** | **Clone** | **Company** | **Catalog number** |
| --- | --- | --- | --- | --- |
| Actin | Human, Mouse | 13E5 | Cell Signaling Technology | 4970 |
| Calnexin | Human, Mouse | N/A | Abcam | ab22595 |
| ERK1/2 | Human, Mouse | 137F5 | Cell Signaling Technology | 4695S |
| p-ERK1/2 (Thr202/ Tyr204) | Human, Mouse | 197G2 | Cell Signaling Technology | 4377S |
| FLAG | N/A | D6W5B | Cell Signaling Technology | 14793 |
| GAPDH | Human, Mouse | D16H11 | Cell Signaling Technology | 5174S |
| HA | N/A | C29F4 | Cell Signaling Technology | 3724S |
| NPC1 | Human, Mouse | EPR5209 | Abcam | ab134113 |
| LAMP1 | Human | E-5 | Santacruz | sc-17768 |
| LAMP1 | Mouse | 1D4B | Santacruz | sc-19992 |
| LC3B | Human, Mouse | D11 | Cell Signaling Technology | 3868S |
| MYD88 | Mouse | D80F5 |  | 4283S |
| p62/SQSTM1 | Mouse | D6M5X | Cell Signaling Technology | 23214S |
| PDI | Human, Mouse | C81H6 | Cell Signaling Technology | 3501S |
| Syntenin-1 | Mouse | N/A | Abcam | ab19903 |
| TLR7 | Mouse | D7 | Cell Signaling Technology | 5632S |
| Ubiquitin | Human, Mouse | P4D1 | Cell Signaling Technology | 3936S |
| K63-linked Ubiquitin | Human, Mouse | D7A11 | Cell Signaling Technology | 5621S |
| UNC93B1 | Human, Mouse | 2C13 | Millipore-Sigma | ZRB1882 |
