# Supplementary material for: Large-scale mutational analysis identifies UNC93B1 variants that drive TLR-mediated autoimmunity in mice and humans
Source: J Exp Med. 2024 May 23;221(8):e20232005. doi: 10.1084/jem.20232005 (PMC11116816; doi:10.1084/jem.20232005)
Supplement: Table S3 — shows histologic scoring of glomerulonephritis. [file JEM_20232005_TableS3.docx]

**Table S3. Histologic scoring of glomerulonephritis.**

|  | **Histologic Finding** | **+/+**  **(n=6)** | **+/R336C**  **(n=15)** | **R336C/R336C**  **(n=6)** |
| --- | --- | --- | --- | --- |
|  | *Each finding scored 0-4; mean and range are listed, cells colored by score* | | | |
| **Glomerulus** | Mesangial proliferation | 0.33 (0-1) | 0.8 (0-2) | 2.33 (1-4) |
|  | Endocapillary hypercellularity | 0 | 0 | 0.5 (0-1) |
|  | Tuft Karyorrhexis | 0 | 0.07 (0-1) | 1.33 (1-2) |
|  | Capillary wall thickening | 0 | 0 | 0.5 (0-1) |
|  | Hyaline thrombi | 0 | 0.07 (0-1) | 0.33 (0-1) |
|  | Sclerosis | 0 | 0.13 (0-1) | 0.66 (0-2) |
| **Inter-stitium** | Inflammatory cell infiltration (cortical interstitium) | 0.17 (0-1) | 0.2 (0-2) | 1.33 (0-3) |
|  | Inflammatory cell infiltration (perivascular interstitium) | 0.33 (0-1) | 0.66 (0-2) | 1.33 (0-3) |
|  | Tubular damage | 0.17 (0-1) | 0.2 (0-3) | 0.83 (0-2) |
|  | Fibrosis | 0 | 0.2 (0-3) | 0.83 (0-2) |
