# Supplementary material for: Large-scale mutational analysis identifies UNC93B1 variants that drive TLR-mediated autoimmunity in mice and humans
Source: J Exp Med. 2024 May 23;221(8):e20232005. doi: 10.1084/jem.20232005 (PMC11116816; doi:10.1084/jem.20232005)
Supplement: Data S2 — describes proband clinical histories. [file JEM_20232005_DataS2.pdf]

Proband Clinical Histories

Proband A.III.1 is an otherwise healthy female of Northern European ancestry, presently 21 years of age, who at age 3 months (following cessation of breastfeeding) developed a photosensitive rash comprising edematous, erythematous papules and plaques, distributed over the face and arms. A biopsy showed interface dermatitis with focal vacuolar change along the dermal-epidermal junction, dermal mucin deposition and clusters of CD123+ plasmacytoid dendritic cells, altogether consistent with tumid lupus. Antinuclear antibody (ANA) testing was negative; mild hypocomplementemia was noted (C4 16.9mg/dL, lower reference level 20mg/dL, C3 within normal limits). On serial clinical and laboratory assessment into adolescence no further symptoms (articular, renal,or others) emerged to suggest systemic involvement of lupus disease. She was last seen at age 16, at which time near-complete resolution of cutaneous symptoms had been achieved with hydroxychloroquine treatment.

A female sibling and a male sibling, 3 years and 7 years younger than proband 1 respectively, developed similar cutaneous symptoms at age 12 months (also following cessation of breastfeeding), but contrastingly were both found to have ANA seropositivity at a titer of 1:80 in a homogenous staining pattern, with negative testing for dsDNA, Smith-RNP, U1-RNP, SSA and SSB autoantibodies. Their cutaneous symptoms similarly improved with hydroxychloroquine treatment, and they had no clinical or laboratory evidence of systemic lupus disease at the time of last assessment at CMH mr at ages 13 and 9, respectively.

Proband B.III.1 is a 13-year-old female of Indian ancestry with juvenile idiopathic arthritis (JIA), a movement disorder ultimately attributed to a neuroinflammatory process involving the basal ganglia, and chronic cutaneous lupus erythematosus (chillblains/perniosis and tumid lupus).

She was diagnosed with extended oligoarticular JIA at age 3, at which time an MRI of the lower extremities to evaluate delayed walking revealed effusion and synovial enhancement in the bilateral ankle, midfoot and metatarsal-phalangeal joints. She subsequently developed involvement of her temporomandibular joints at age 4 (compromising food intake and growth) and her wrists at age 6. She was treated serially with glucocorticoids, hydroxychloroquine (therapy ongoing), methotrexate, azathioprine, mycophenolate mofetil, etanercept, rituximab, Intravenous Immunoglobulin (IVIG), tocilizumab, tofacitinib then baricitinib (therapy ongoing), the latter two ultimately allowing glucocorticoid-free weight bearing and limited ambulation.

Her motor function and development were further compromised by a dystonic/ataxic movement disorder, ultimately delaying the acquisition of walking ability until age 5 years. MRI of her brain at ages 3 and 7 showed stable non-specific punctate T2 hyperintensities in the subcortical frontal lobes, and electroencephalogram as well as cerebrospinal fluid and blood testing for known autoimmune encephalitides were normal. At multiple points in the course of her arthritis treatment, her ataxia and dystonia were noted to improve upon high- and moderate-dose glucocorticoid treatment, and also improved on transition to tofacitinib at age 12. Following this, she enrolled in a study exploring the test characteristics of blood monocyte CD169 (Biesen et al., 2008; Sakumura et al., 2023; York et al., 2007) and CD274 expression for predicting serum type I versus type II interferon activity level, respectively. This test revealed a monocyte CD169 mean fluorescence intensity (MFI) 35-fold higher than the upper reference level and a CD274 MFI within normal limits, suggestive of high serum type I interferon and normal type II interferon activity. At age 13, ongoing and progressive ataxia prompted a repeat brain MRI

which revealed calcifications in the bilateral globus pallidi and the right caudate nucleus, reminiscent of those seen in the Aicardi-Goutieres family of monogenic interferonopathies. In light of these two clinical data points suggesting a type I interferonopathy driving her neurologic symptoms, her clinicians transitioned her from tofacitinib to baricitinib (which has a lower IC50 than tofacitinib for the type I IFN receptor IFNAR1 across T cells, NK cells and monocytes) (McInnes et al., 2019). Within 1 month, she was noted to have an improvement in walking ability from partial wheelchair dependence to a 2.5-mile ambulatory capacity with assistive devices.

Her cutaneous symptoms have included photosensitive, erythematous, edematous plaques involving the face, chest and arms, first noted at 11 months of age with biopsies consistent with tumid lupus, as well as chronic cold-temperature sensitive violaceous and painful plaques of the fingertips consistent with perniosis/chillblain lupus. Her tumid lupus has not recurred since starting hydroxychloroquine at age 3, and her chillblain rashes are presently well-controlled on hydroxychloroquine and baricitinib. ANA testing by indirect immunofluorescence was negative at age 7 and age 11, and dsDNA autoantibody testing was negative at age 11. At the time of this writing, testing for Smith-RNP, U1-RNP, SSA, SSB, and proteinuria by urine protein-to-creatinine spot ratio have not been performed.
