# Supplementary figures and images for: Large-scale mutational analysis identifies UNC93B1 variants that drive TLR-mediated autoimmunity in mice and humans
Source: J Exp Med. 2024 May 23;221(8):e20232005. doi: 10.1084/jem.20232005 (PMC11116816; doi:10.1084/jem.20232005)

Figure 2G

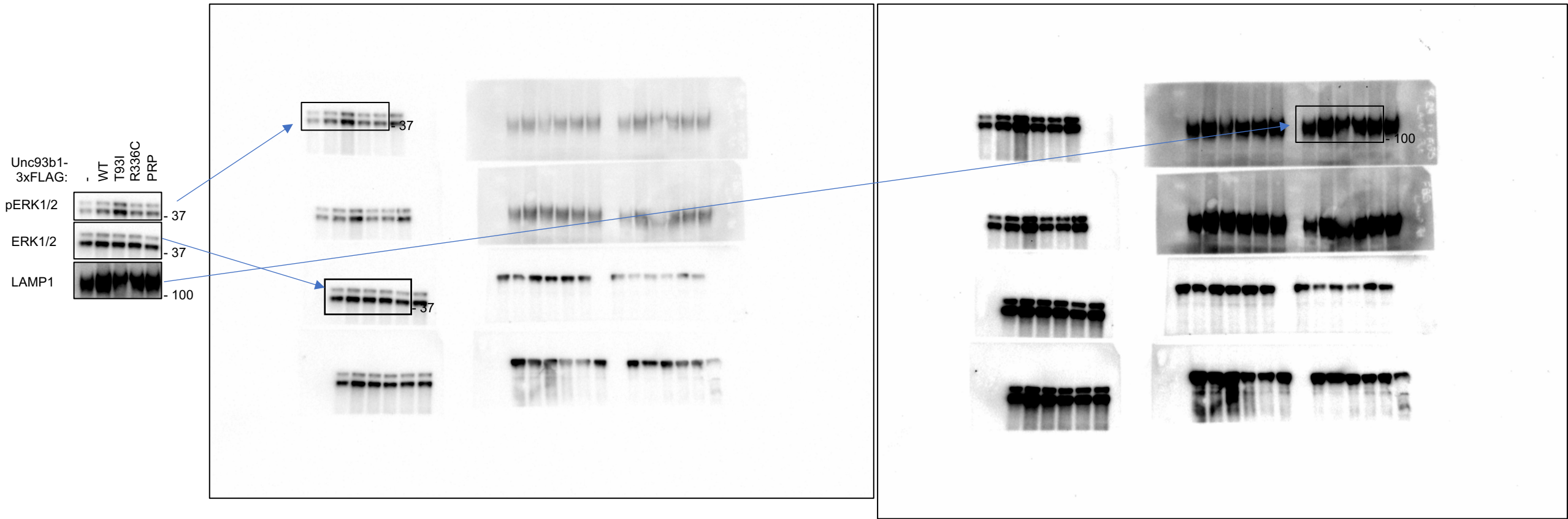

Supplement: SourceData F2 — contains original blots for Fig. 2. [file JEM_20232005_SourceDataF2.pdf]

Figure S1C

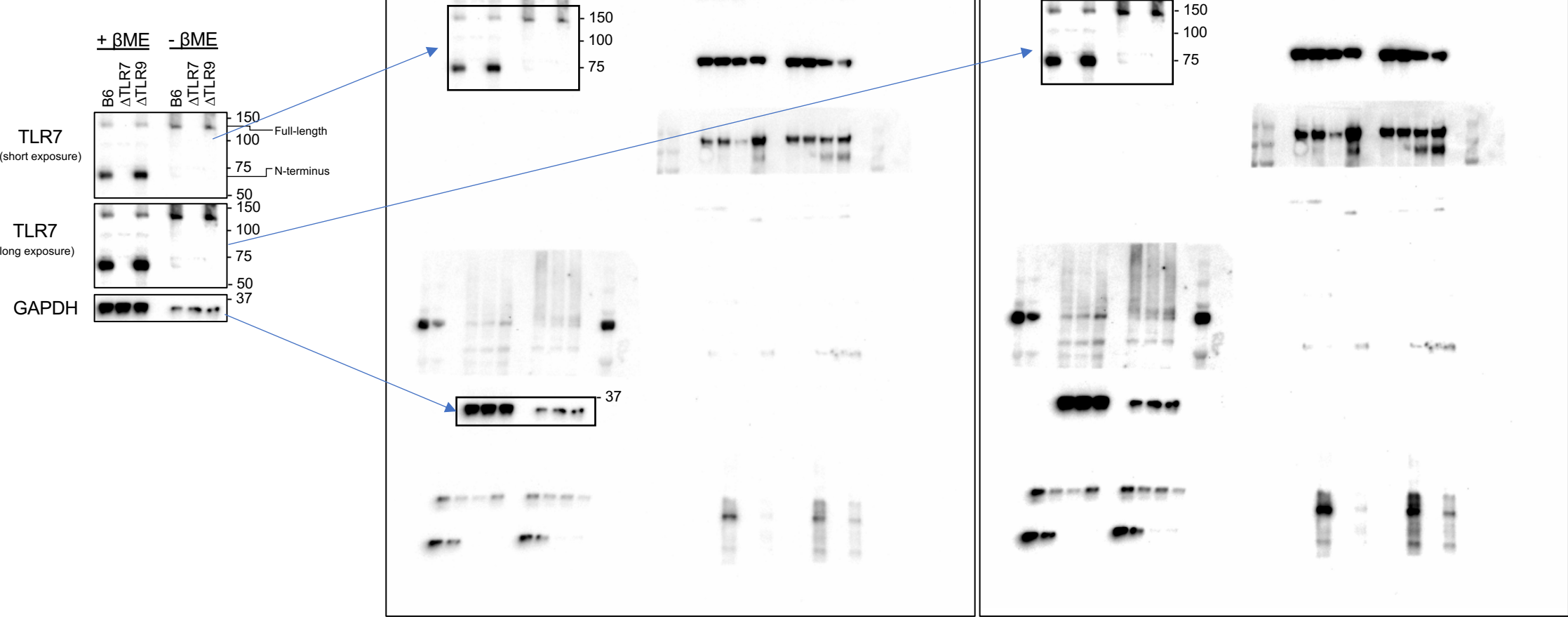

Figure S1D  
Part 1

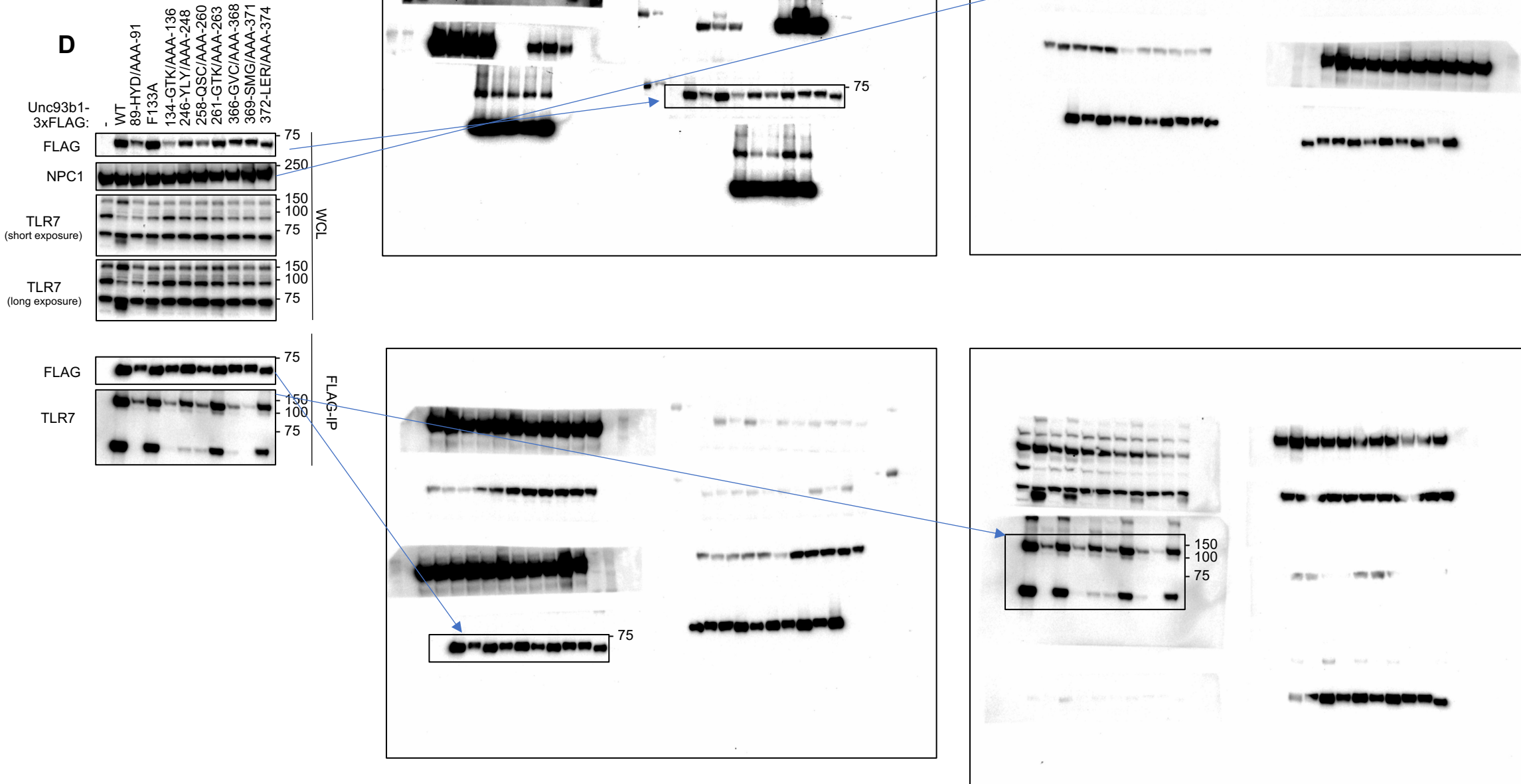

Figure S1D  
Part 2

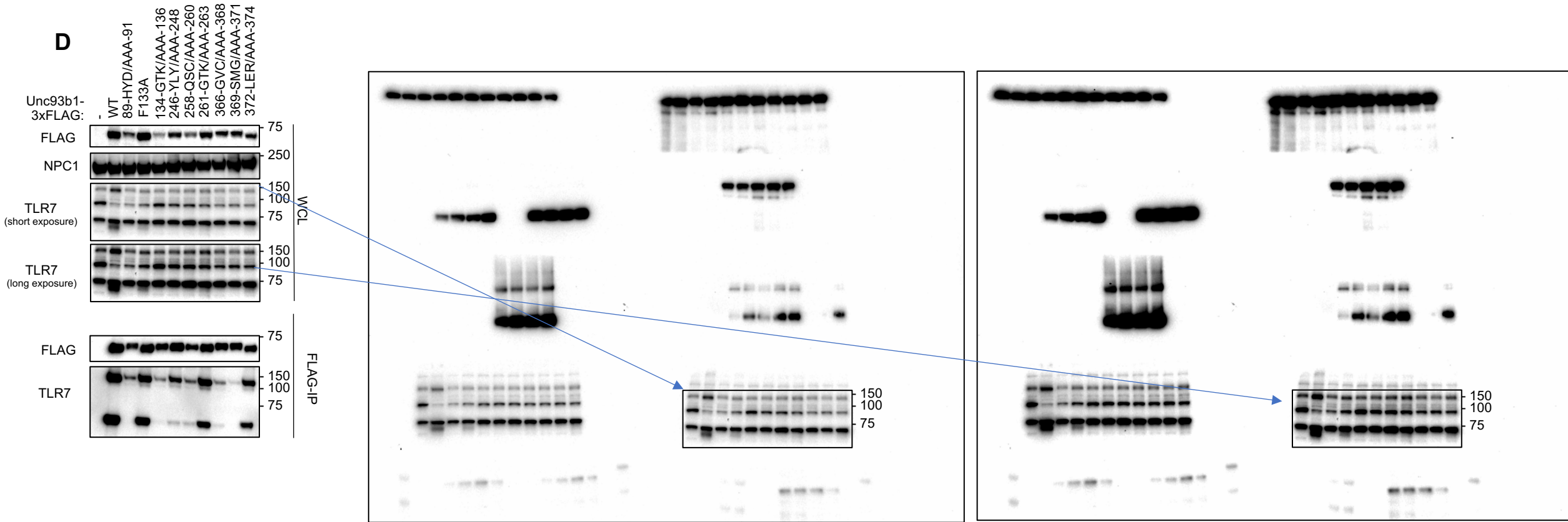

Figure S1F  
Part 1

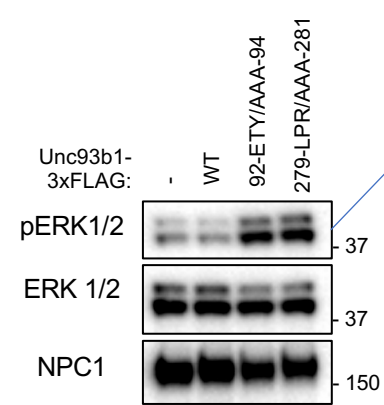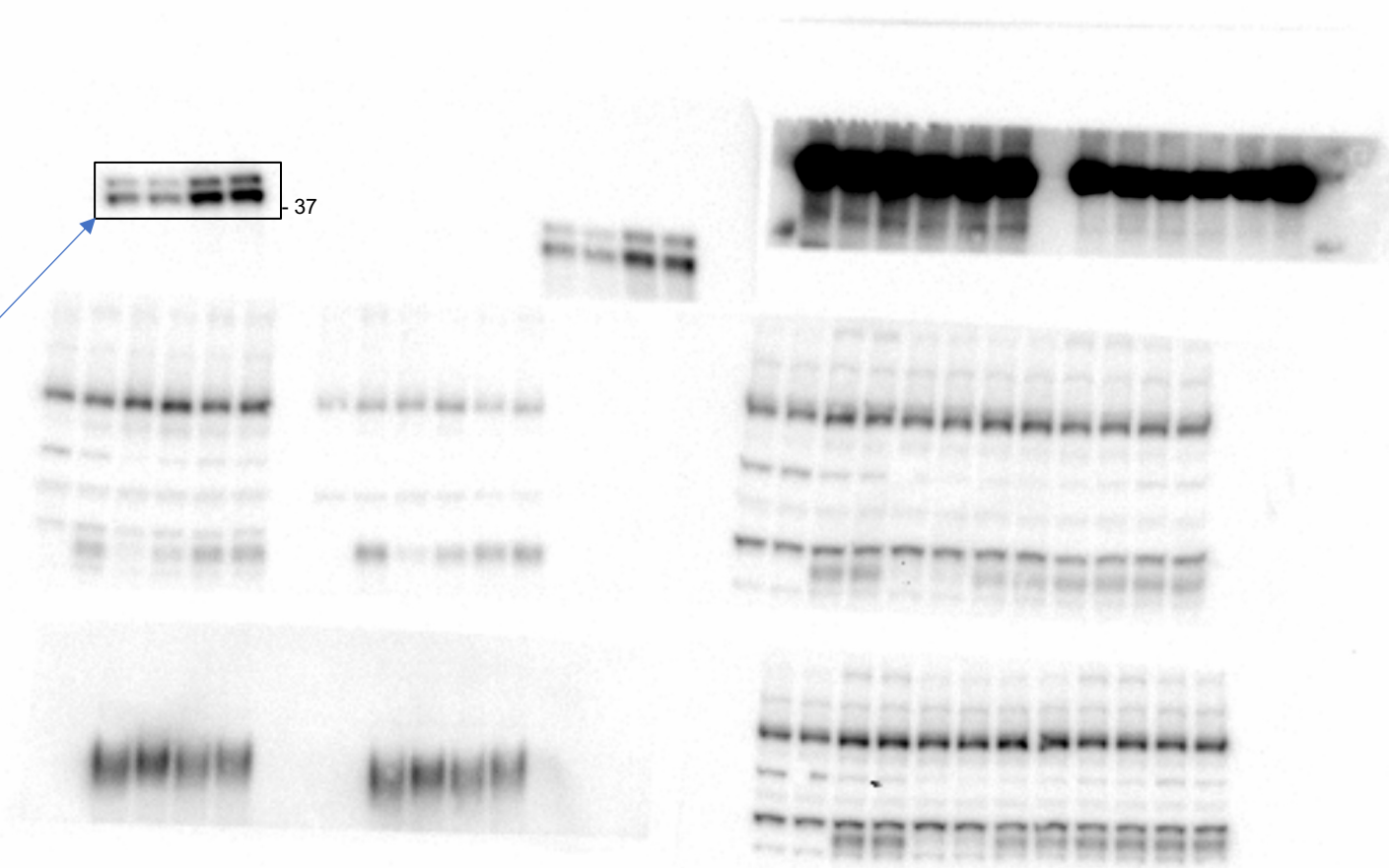

Figure S1F  
Part 2

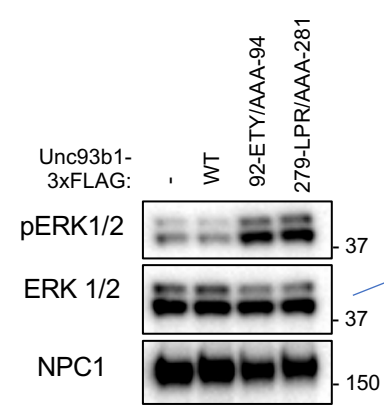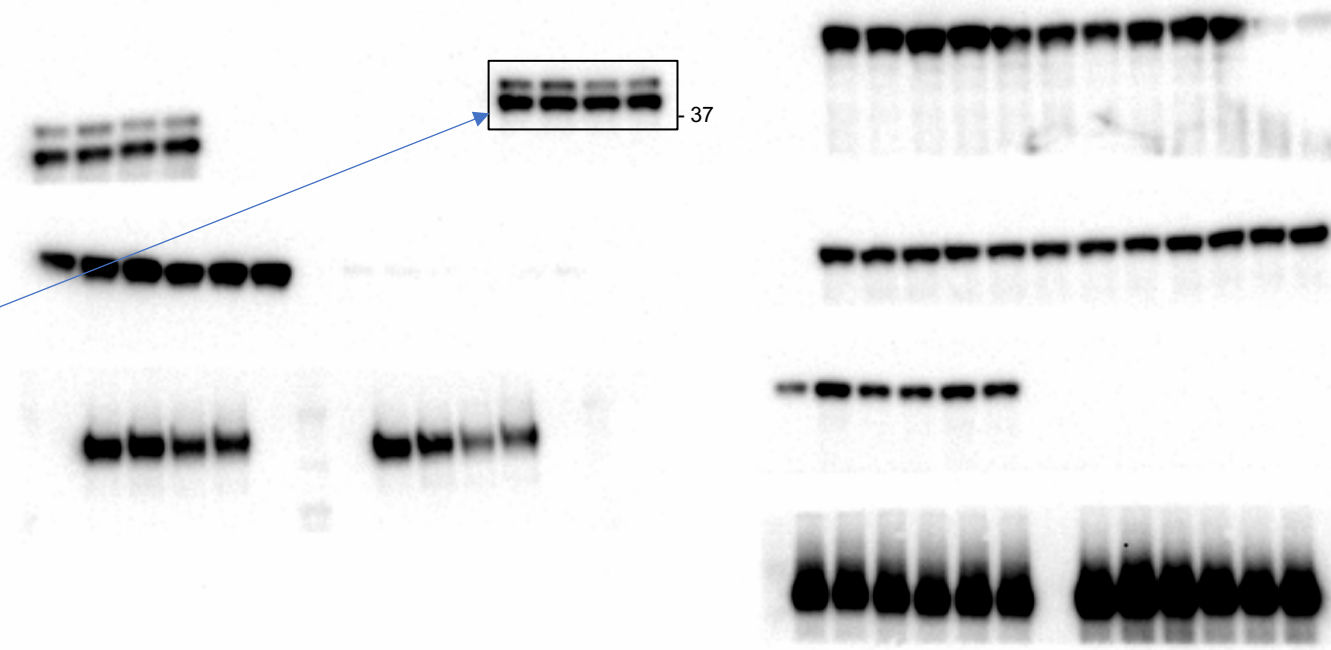

Figure S1F  
Part 3

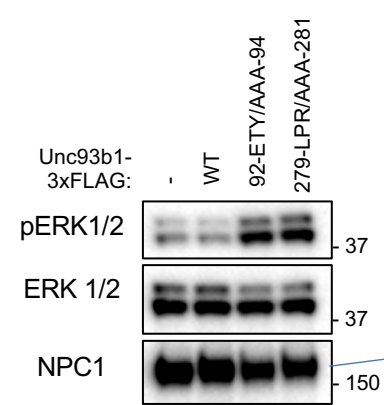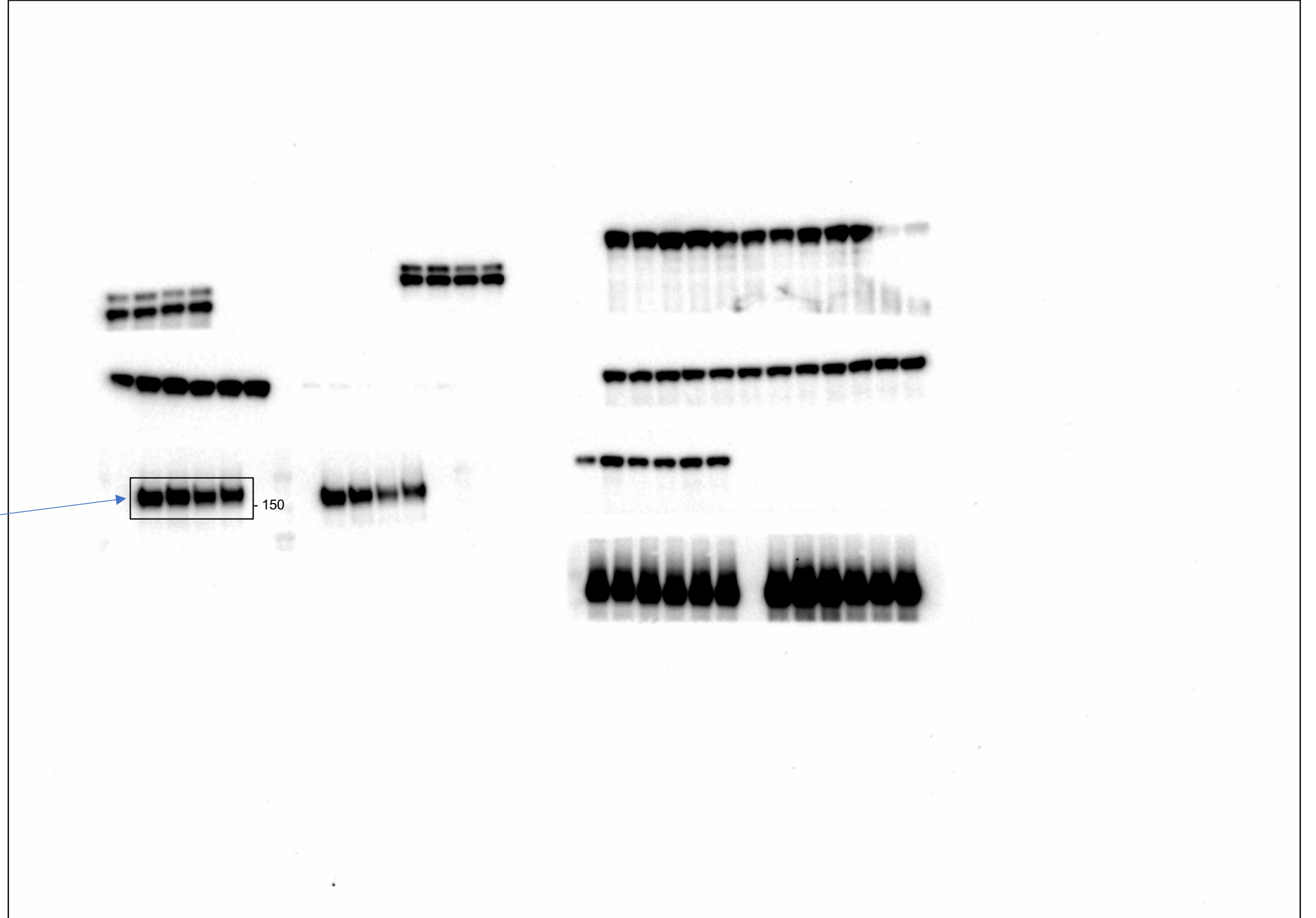

Supplement: SourceData FS1 — contains original blots for Fig. S1. [file JEM_20232005_SourceDataFS1.pdf]

Figure S3A

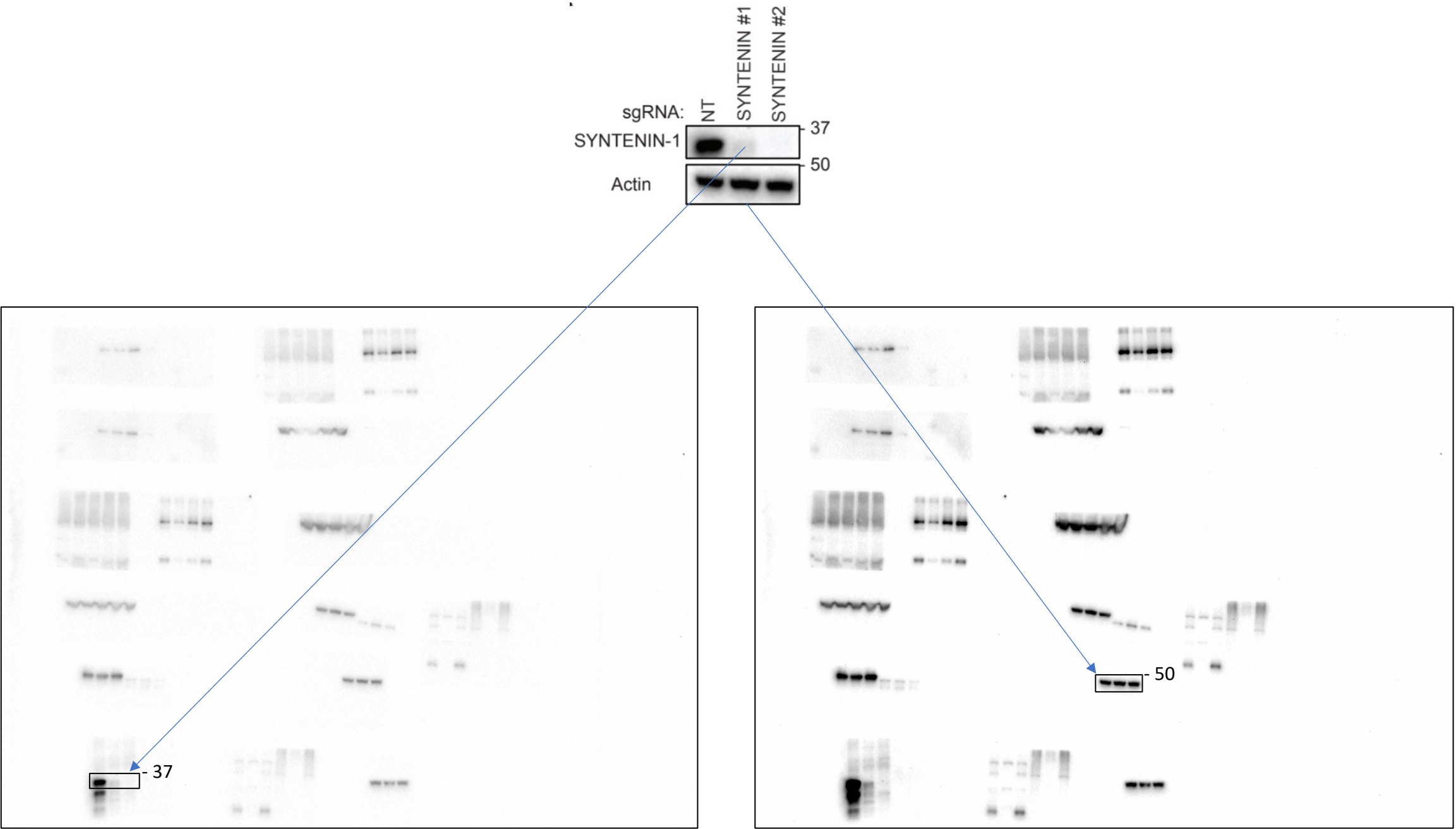

Figure S3B  
Part 1

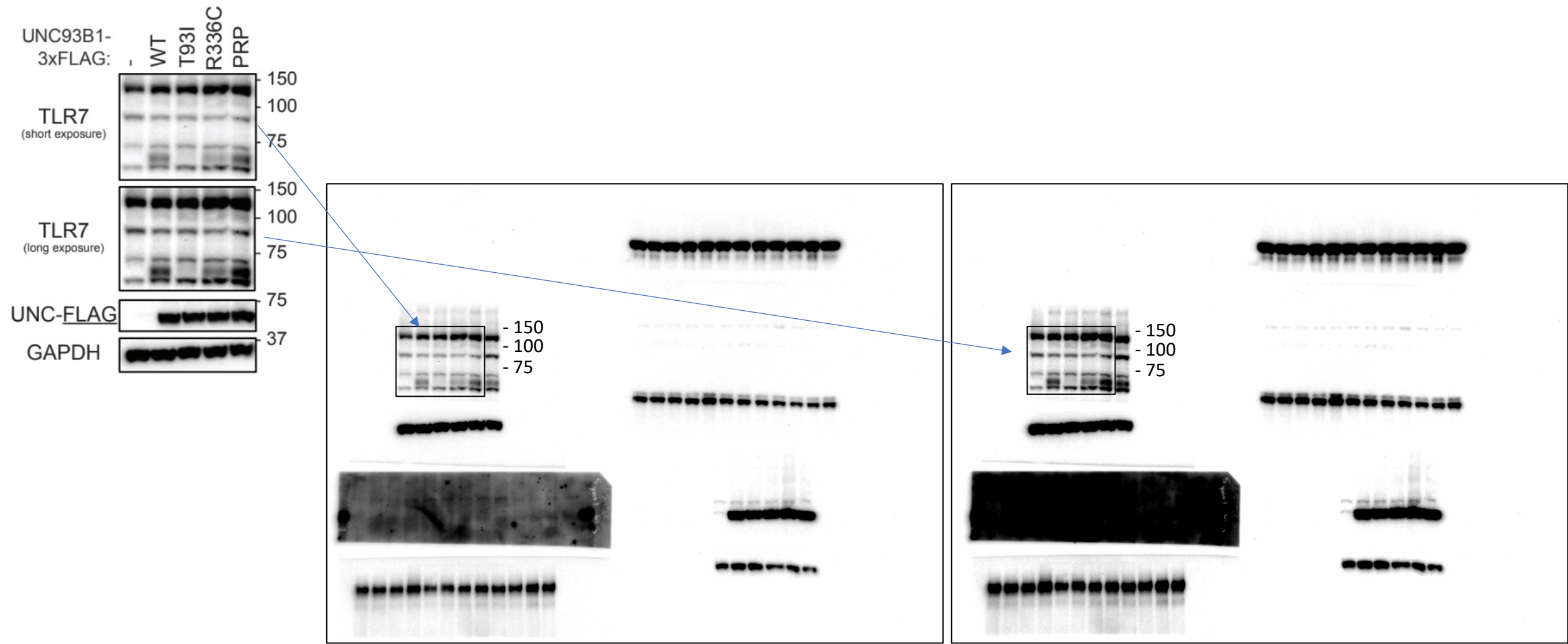

Figure S3B  
Part 2

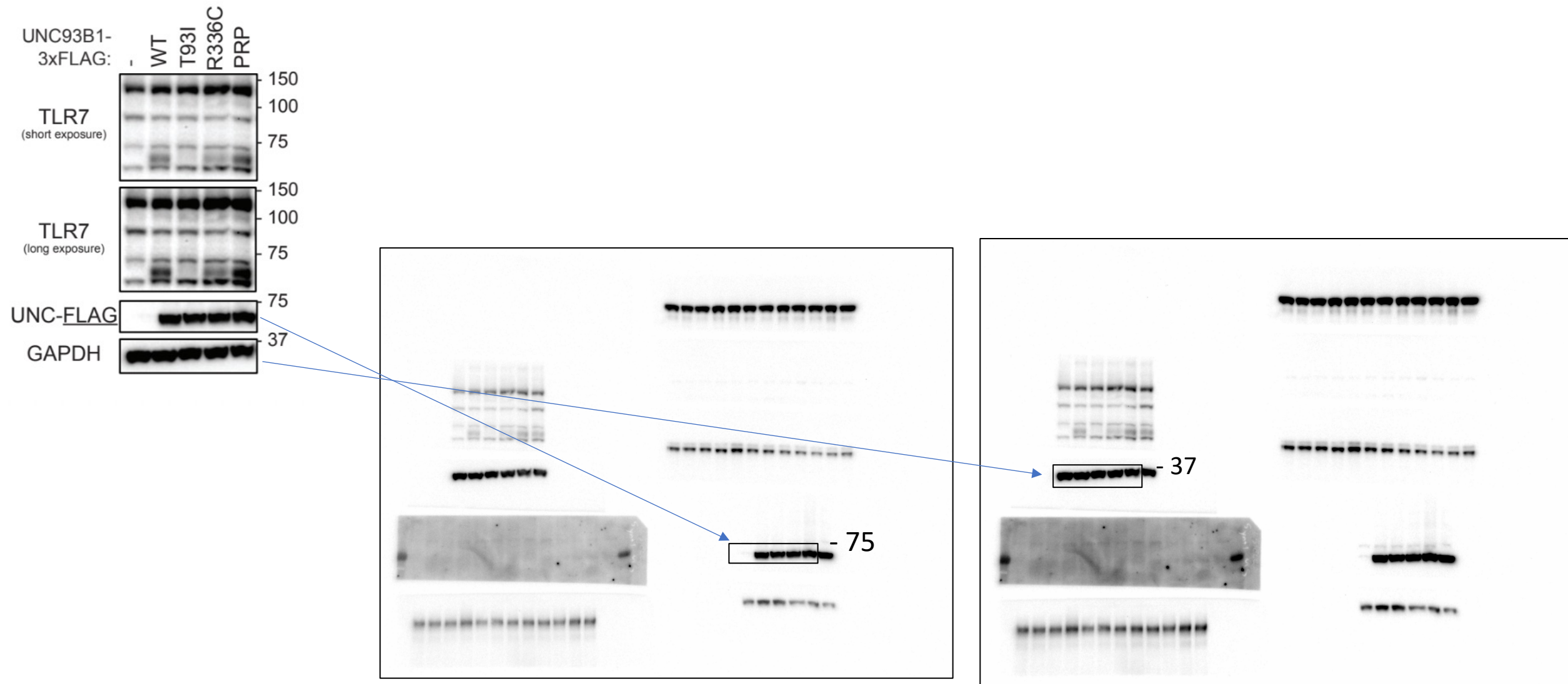

Figure S3C  
Part 1

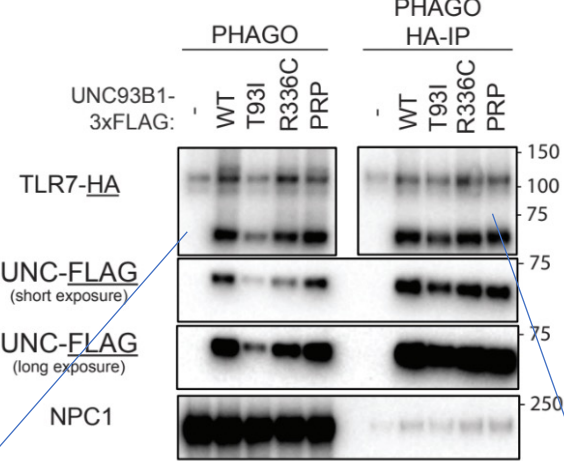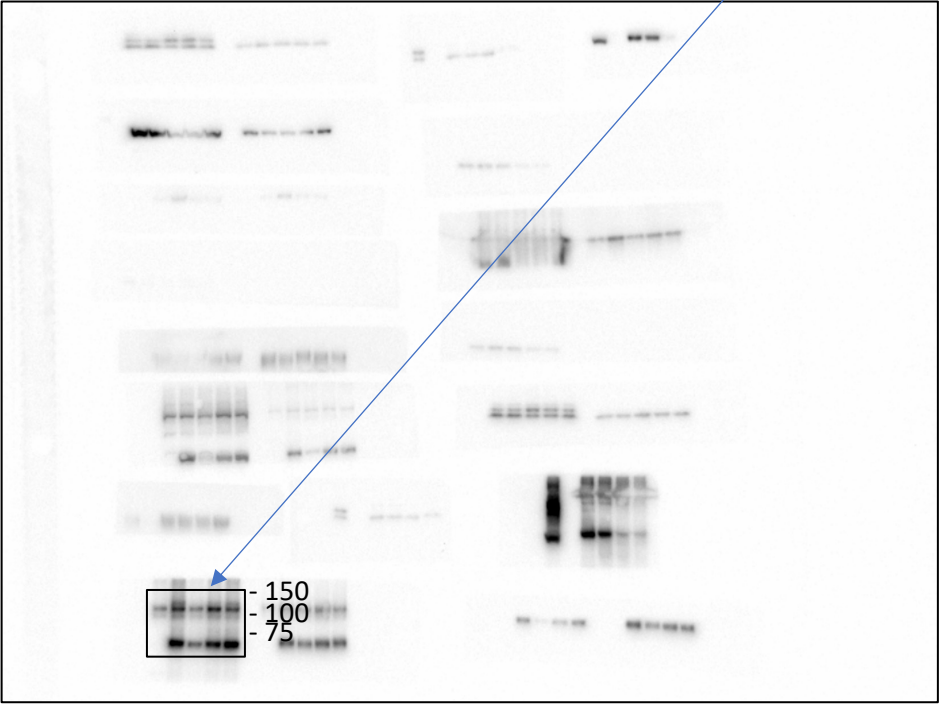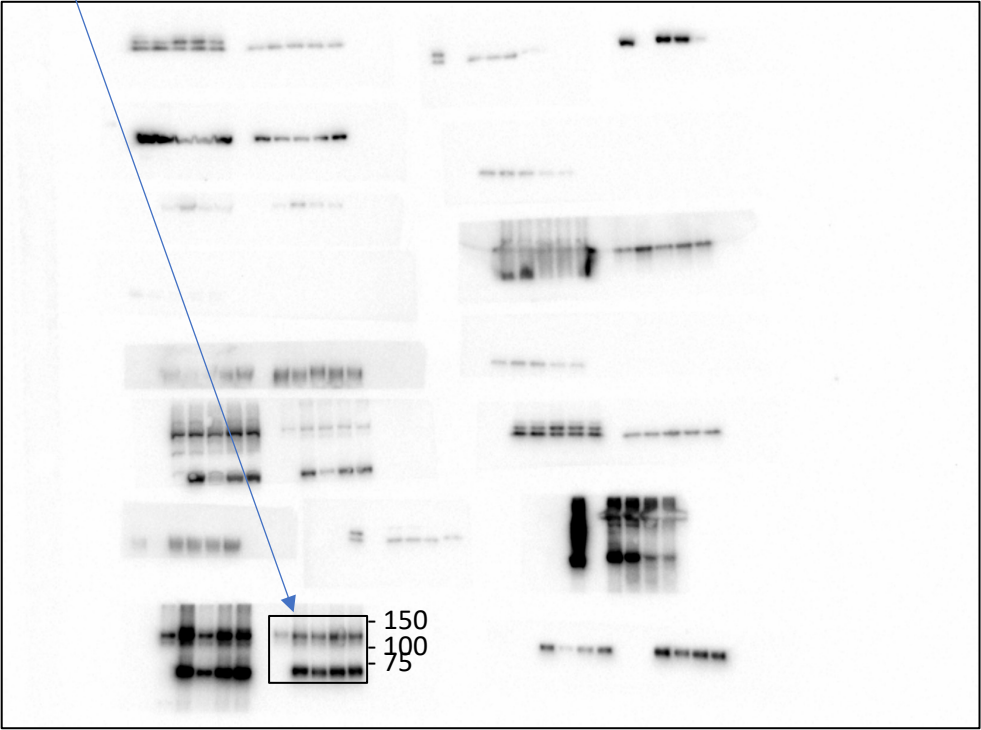

Figure S3C  
Part 2

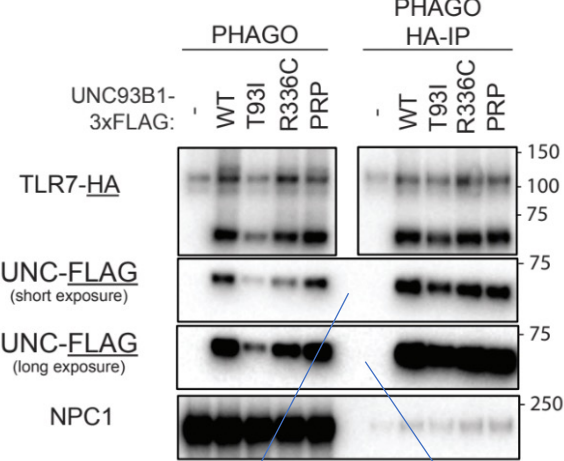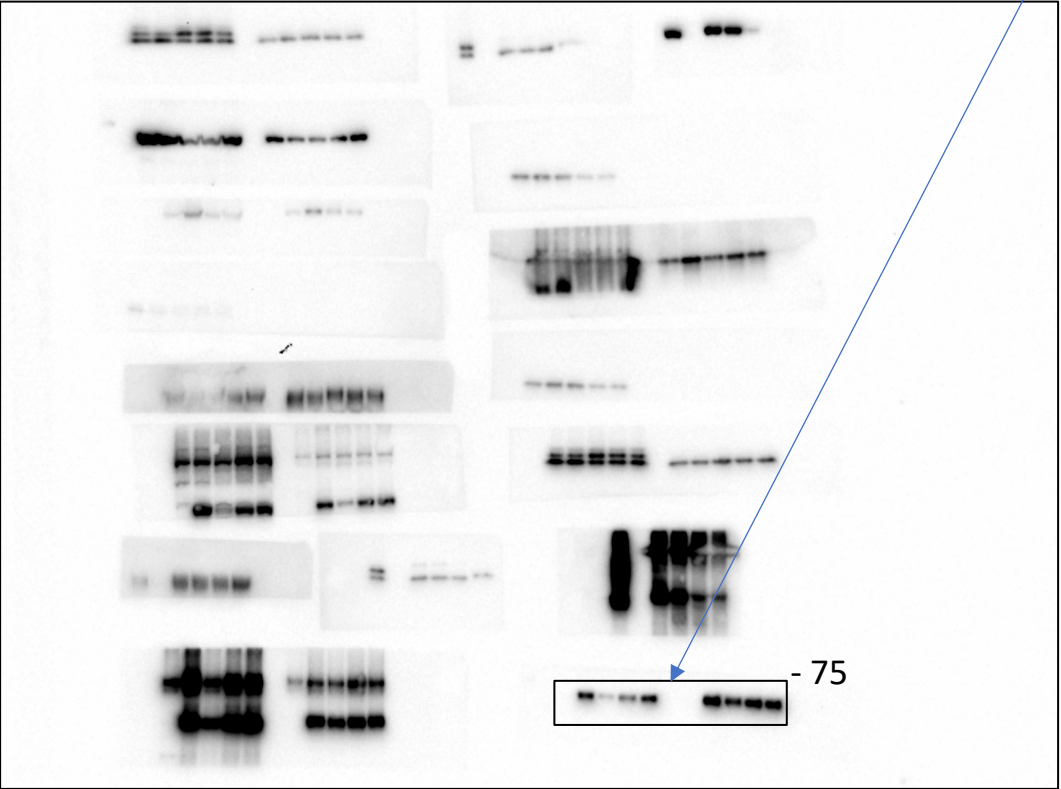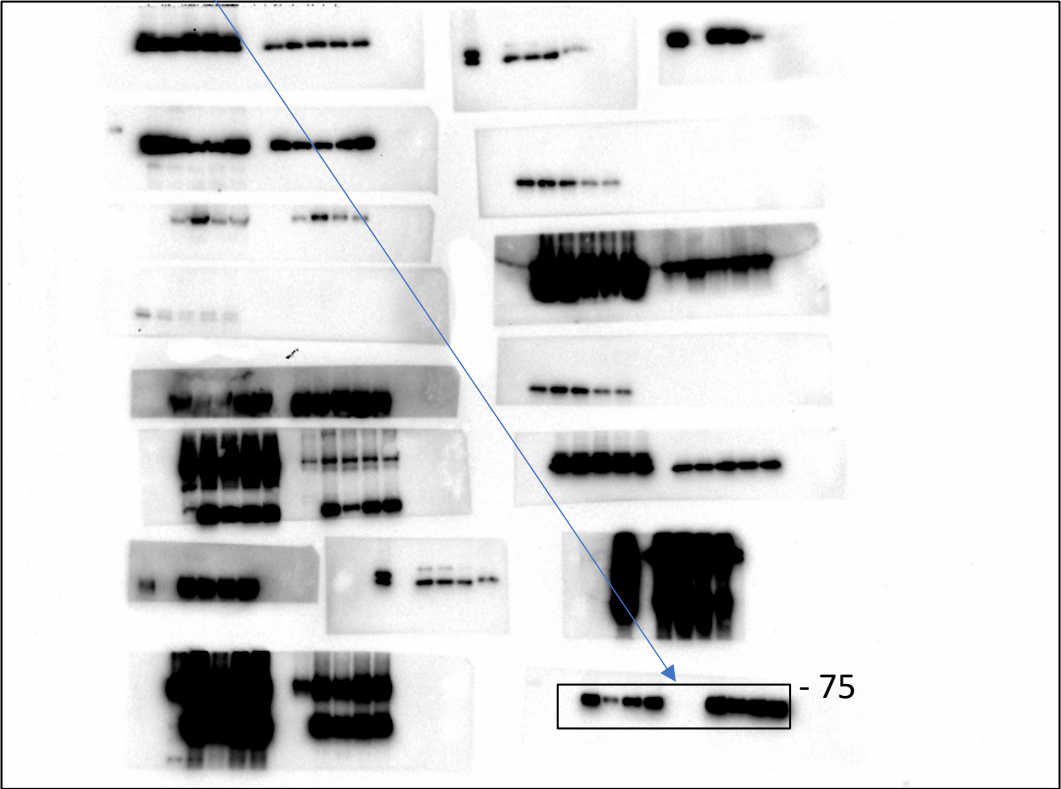

Figure S3C  
Part 3

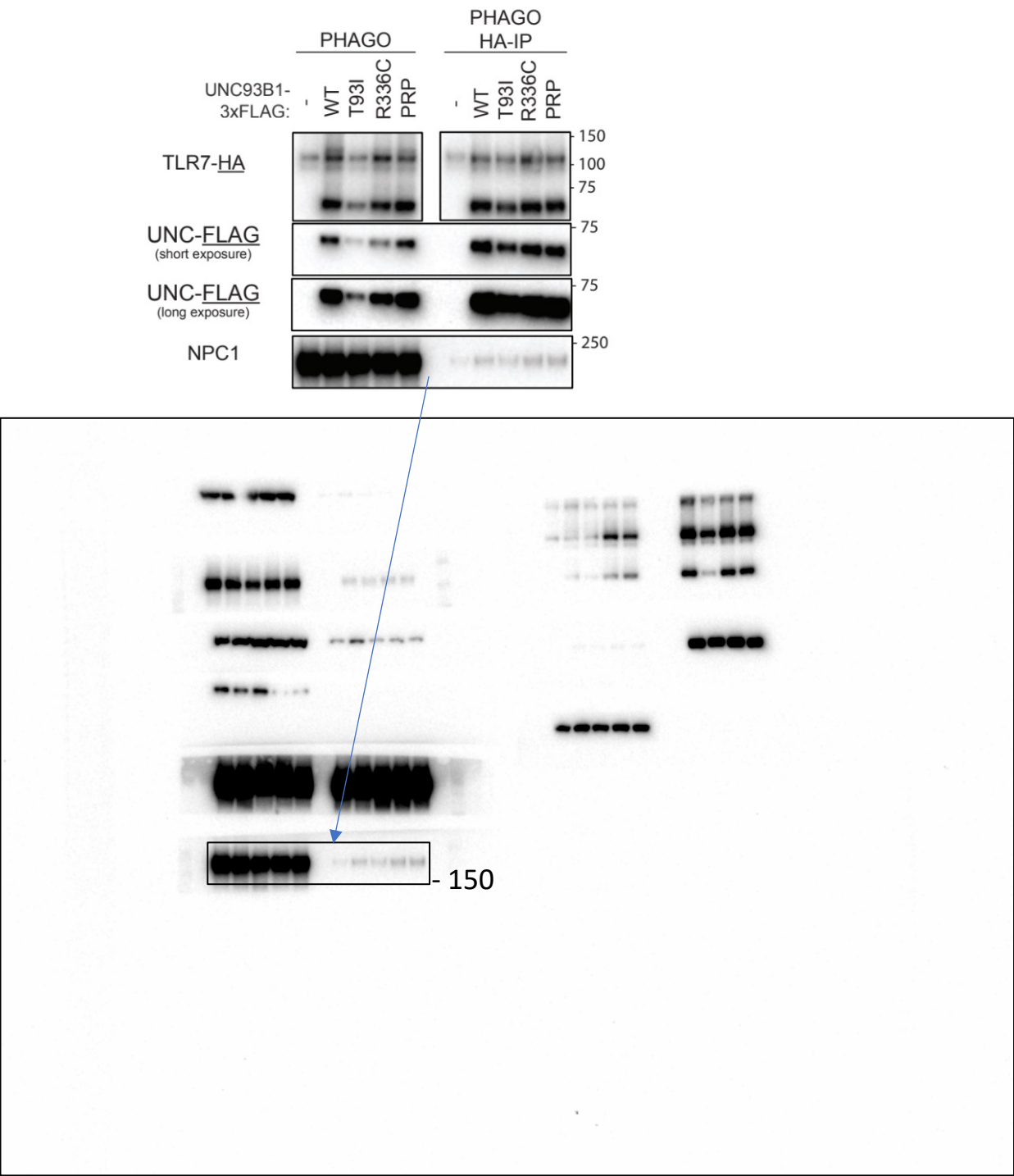

Supplement: SourceData FS3 — contains original blots for Fig. S3. [file JEM_20232005_SourceDataFS3.pdf]
