# Supplementary material for: Large-scale mutational analysis identifies UNC93B1 variants that drive TLR-mediated autoimmunity in mice and humans
Source: J Exp Med. 2024 May 23;221(8):e20232005. doi: 10.1084/jem.20232005 (PMC11116816; doi:10.1084/jem.20232005)

Figure 3A
Part 1

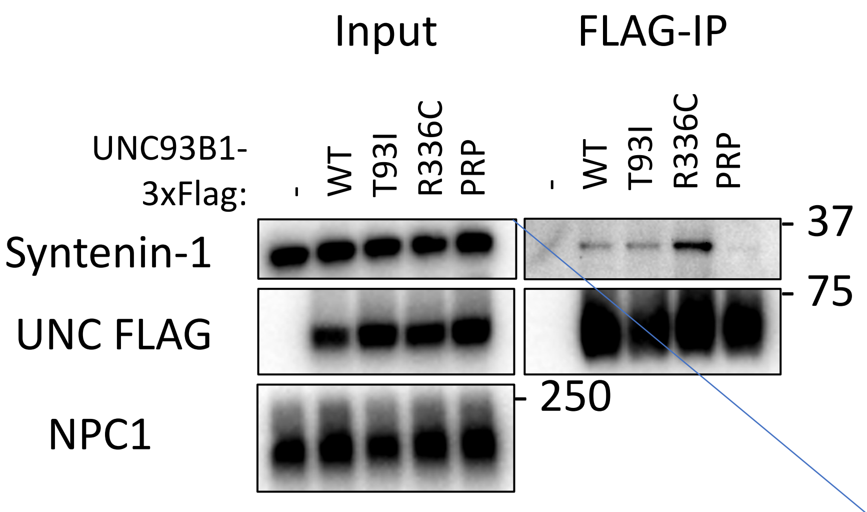

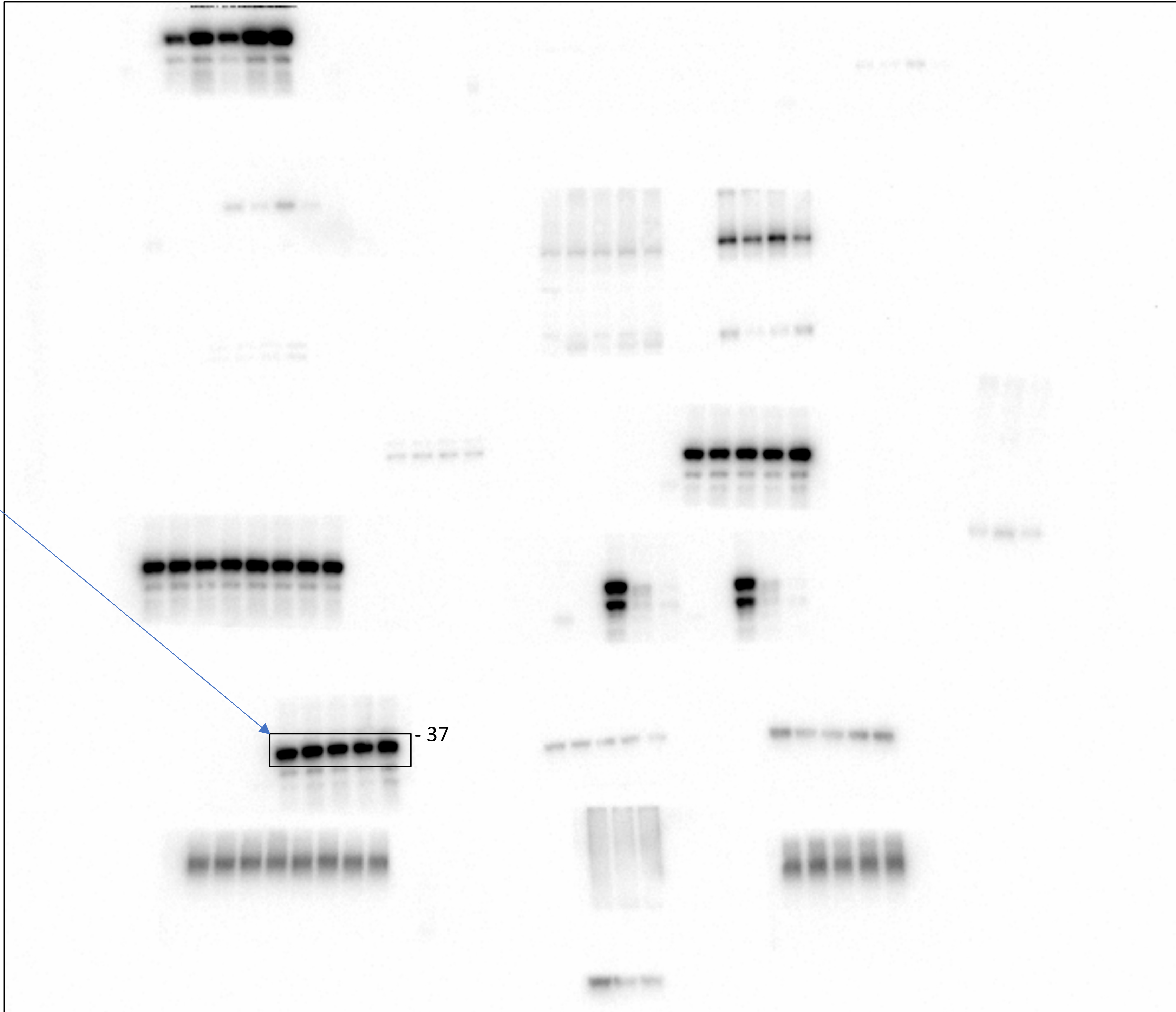

Figure 3A
Part 2

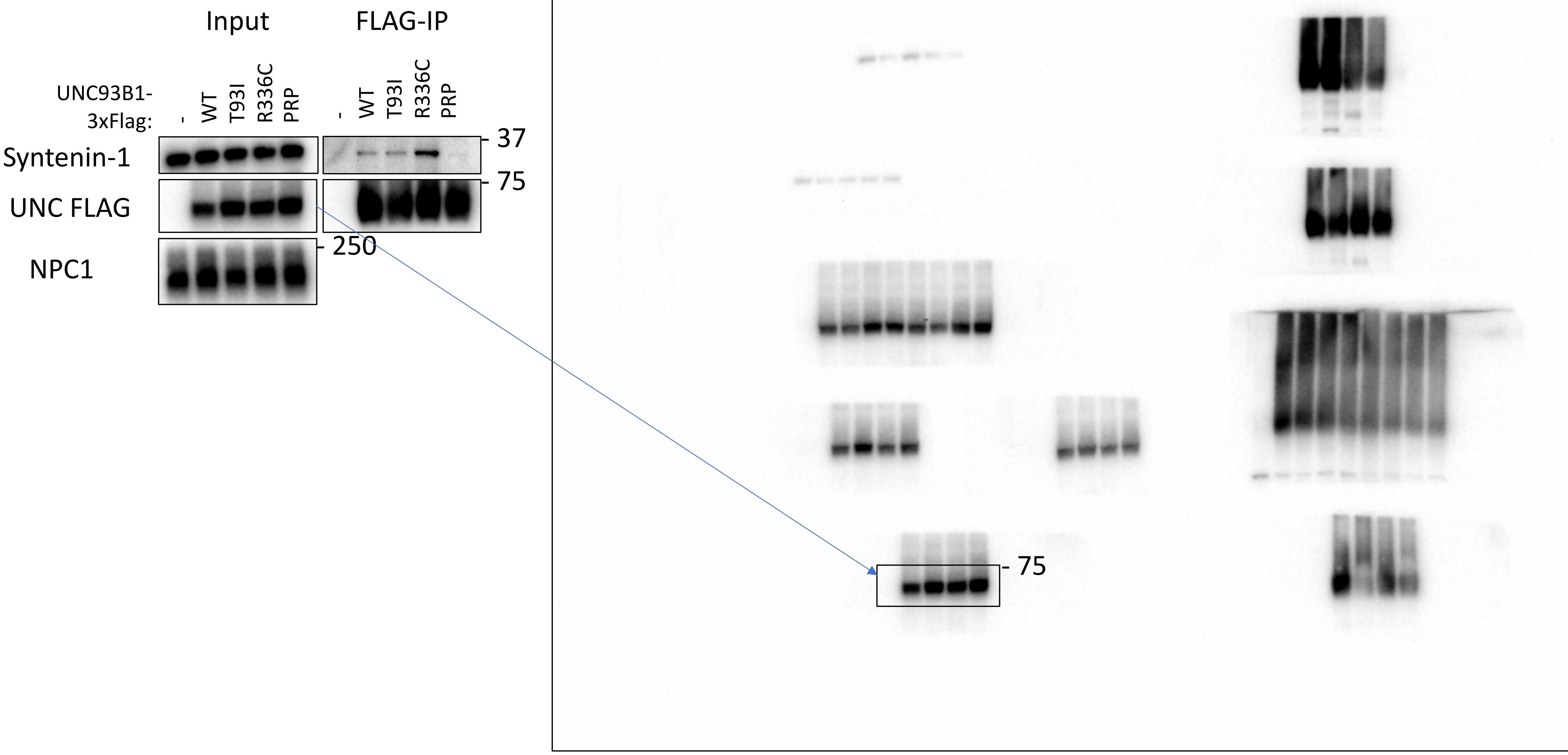

Figure 3A
Part 3

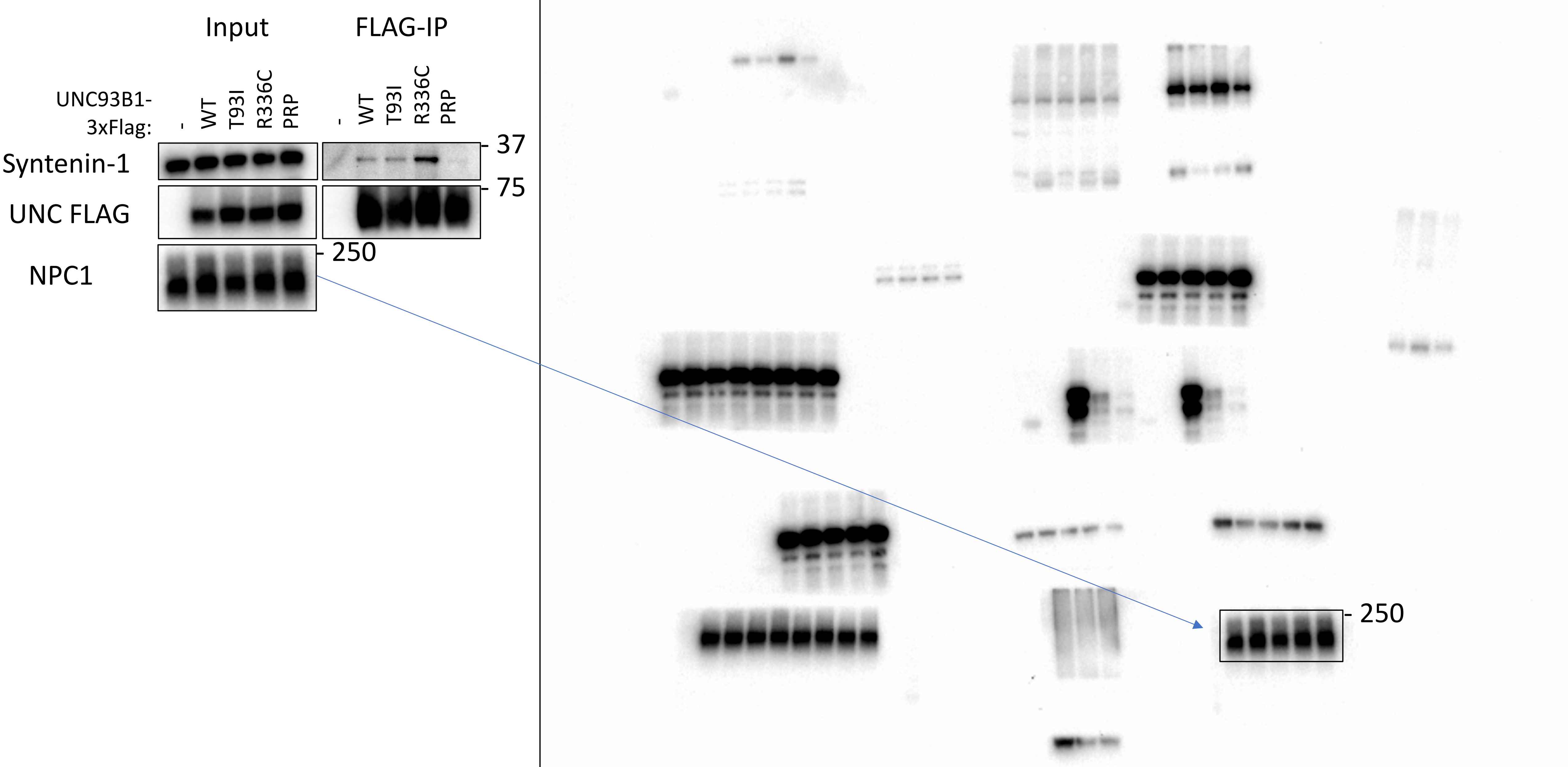

Figure 3A
Part 4

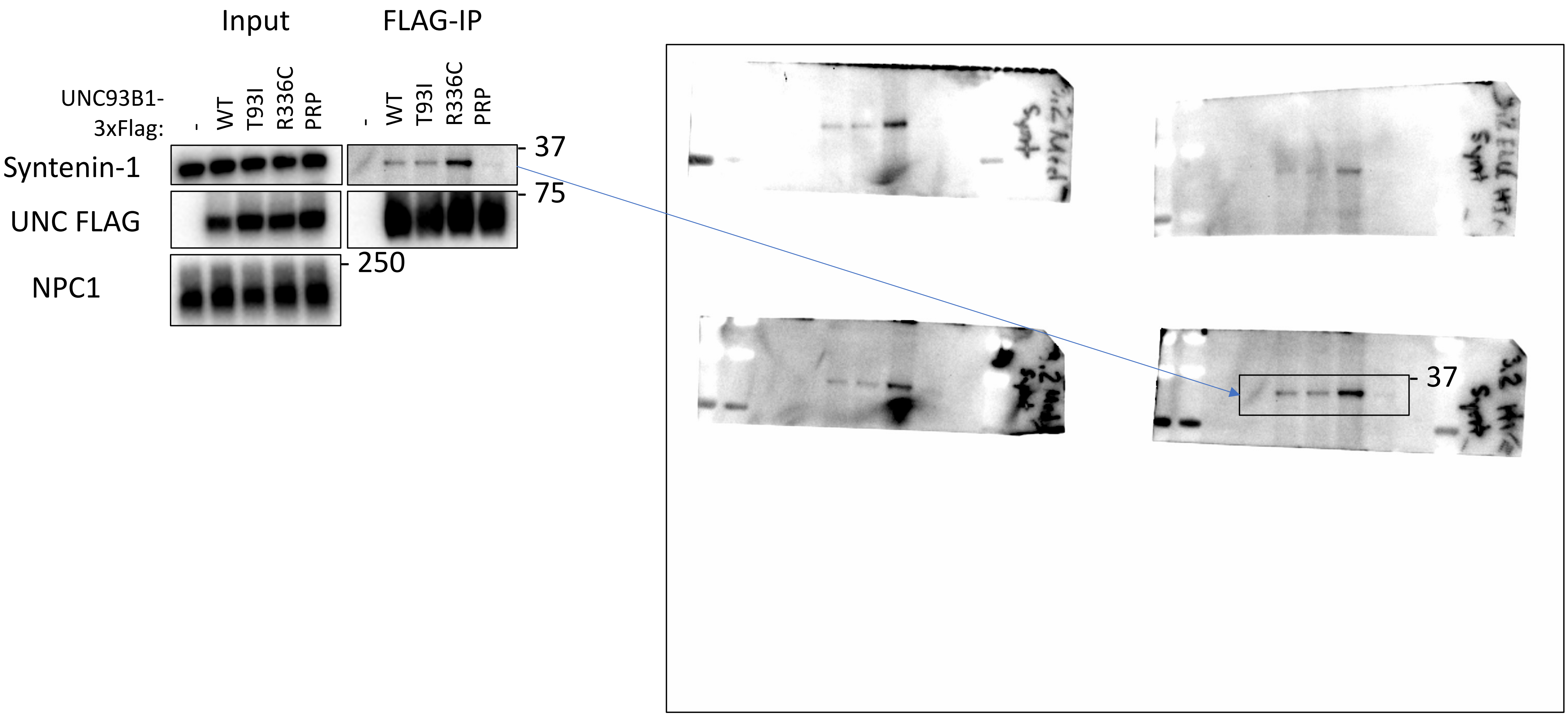

Figure 3A
Part 5

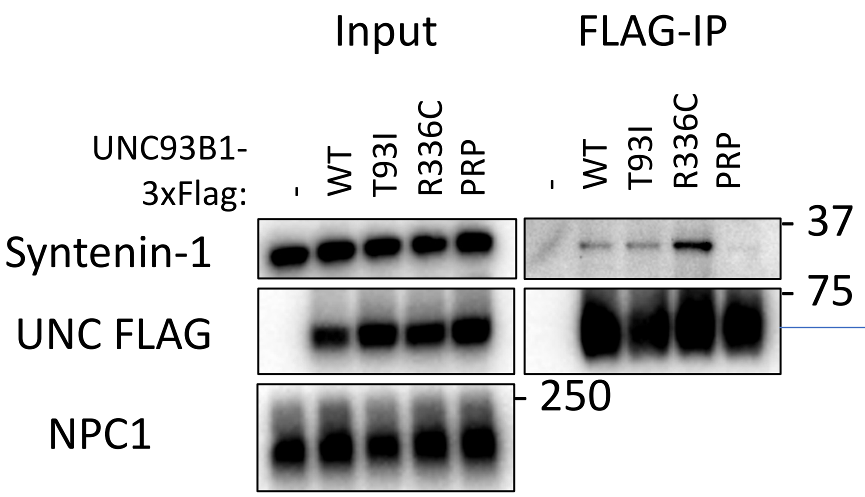

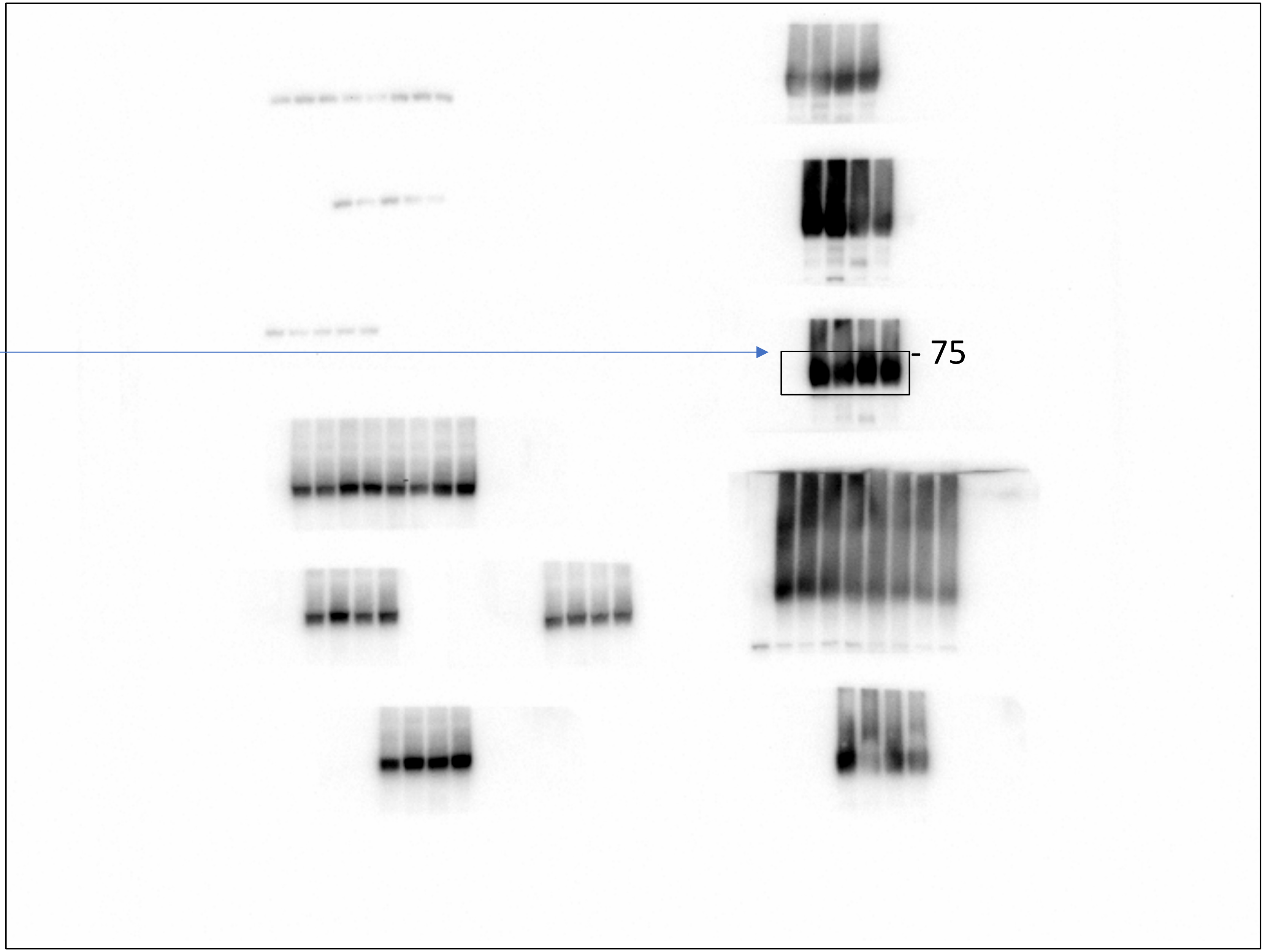

Figure 3C
Part 1

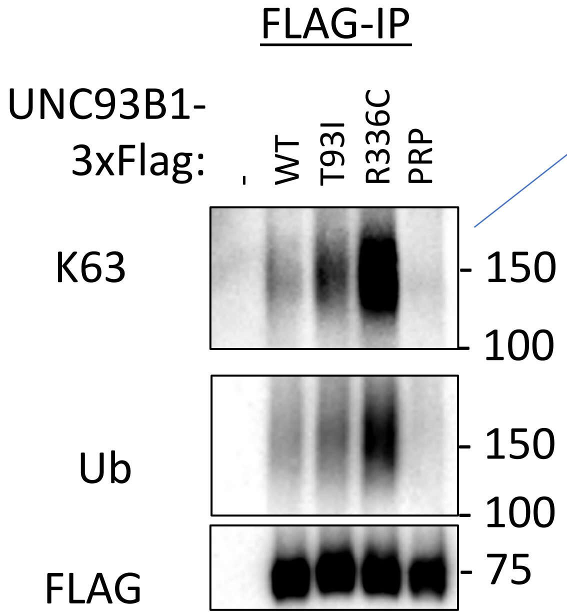

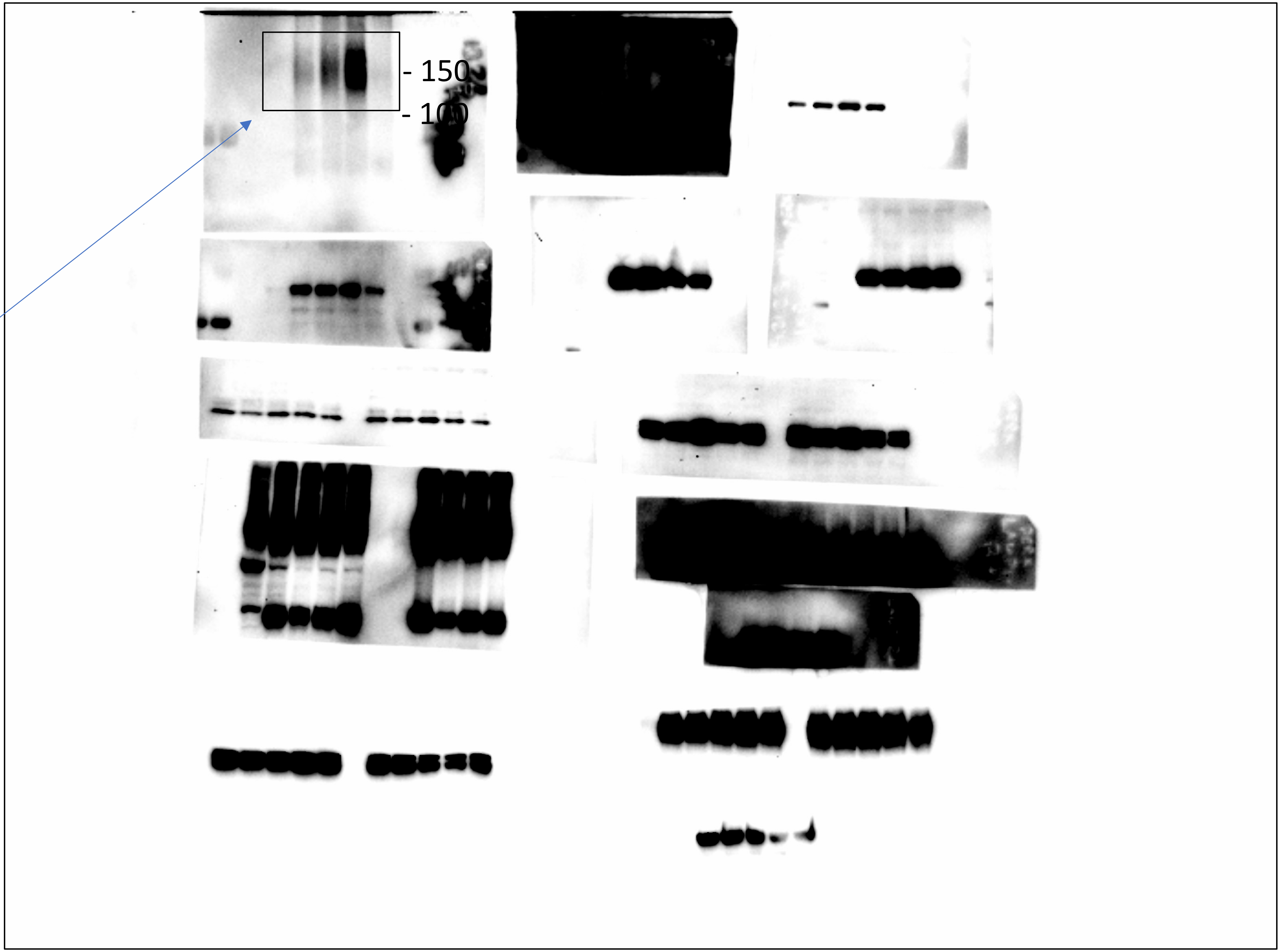

Figure 3C
Part 2

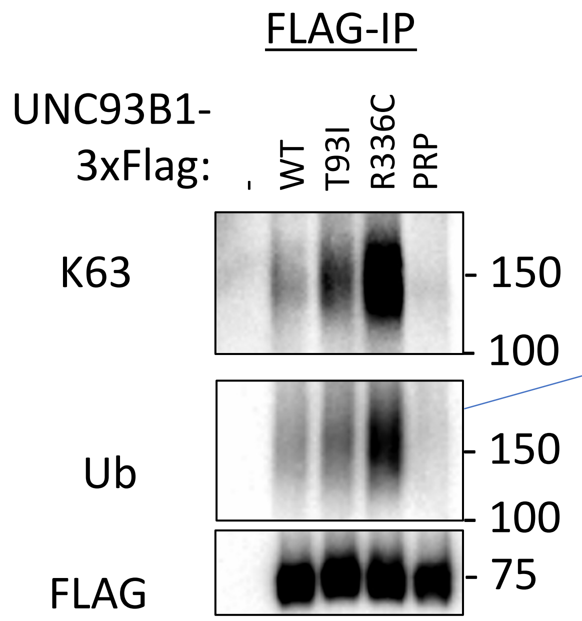

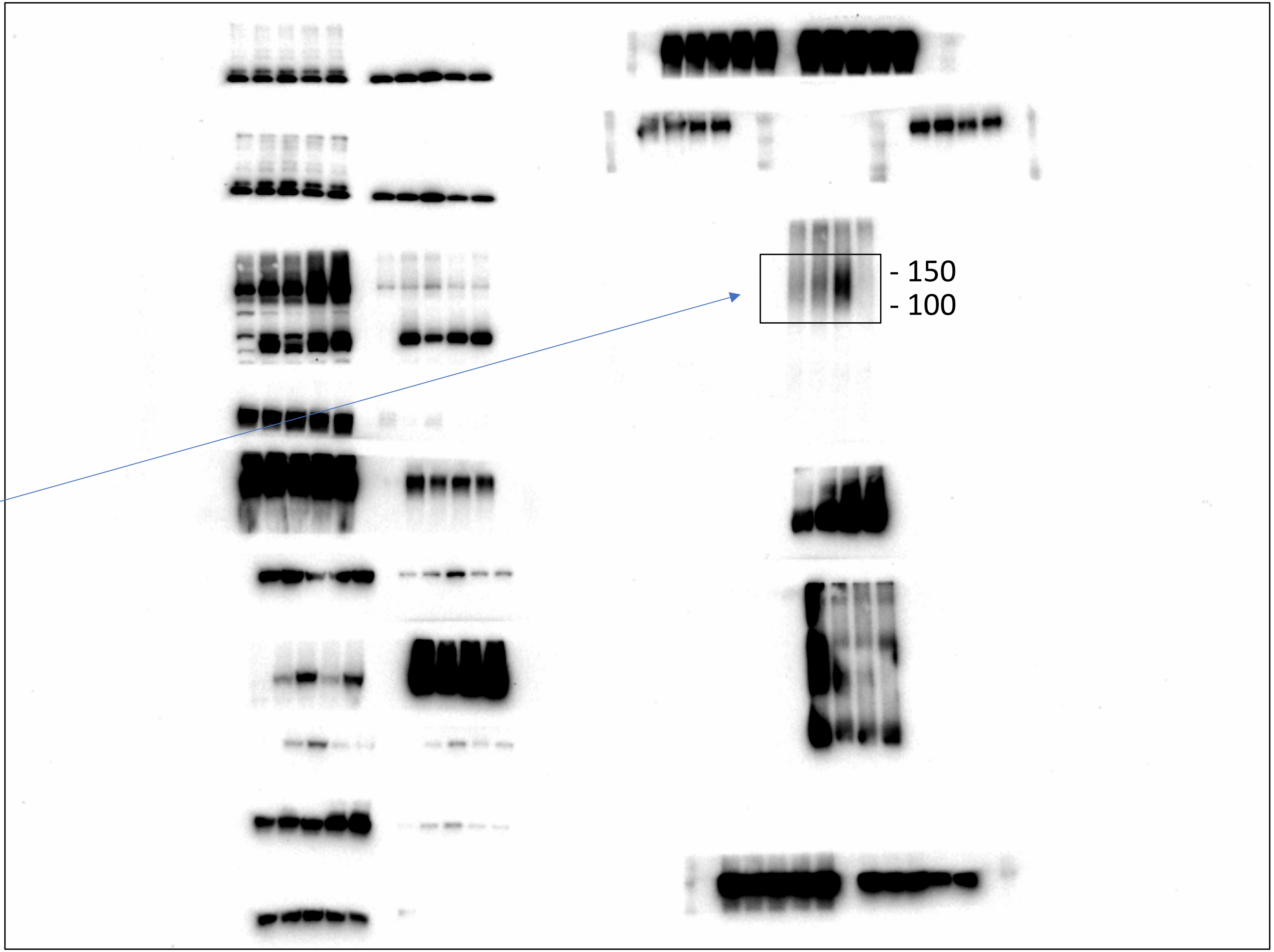

Figure 3C
Part 3

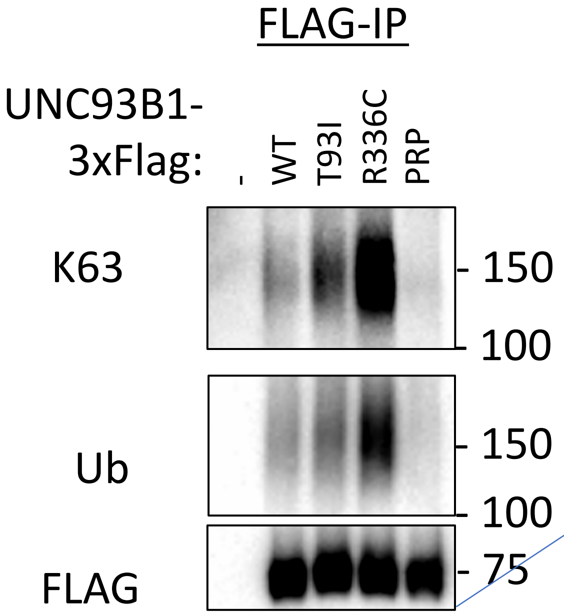

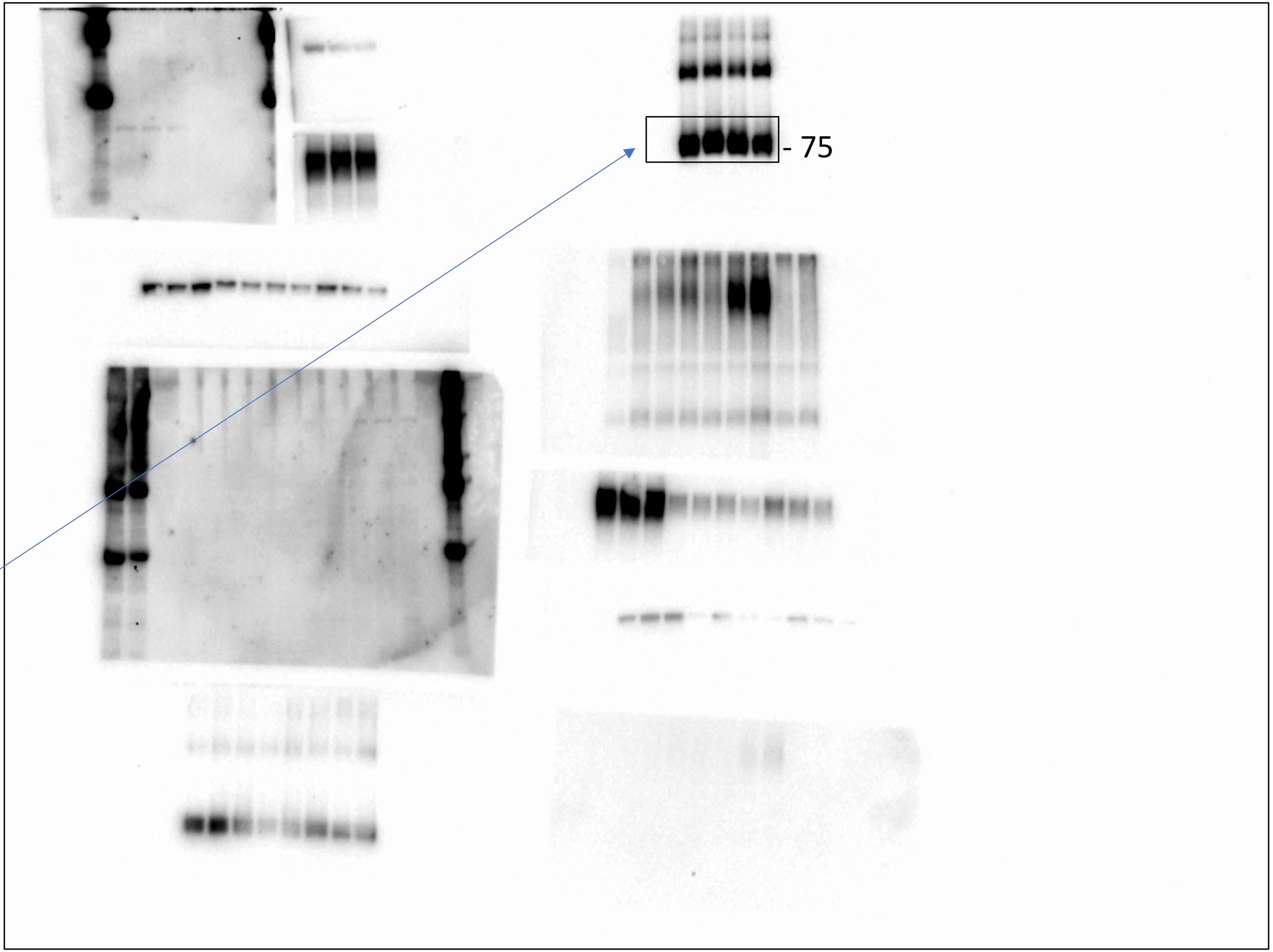

Figure 3D
Part 1

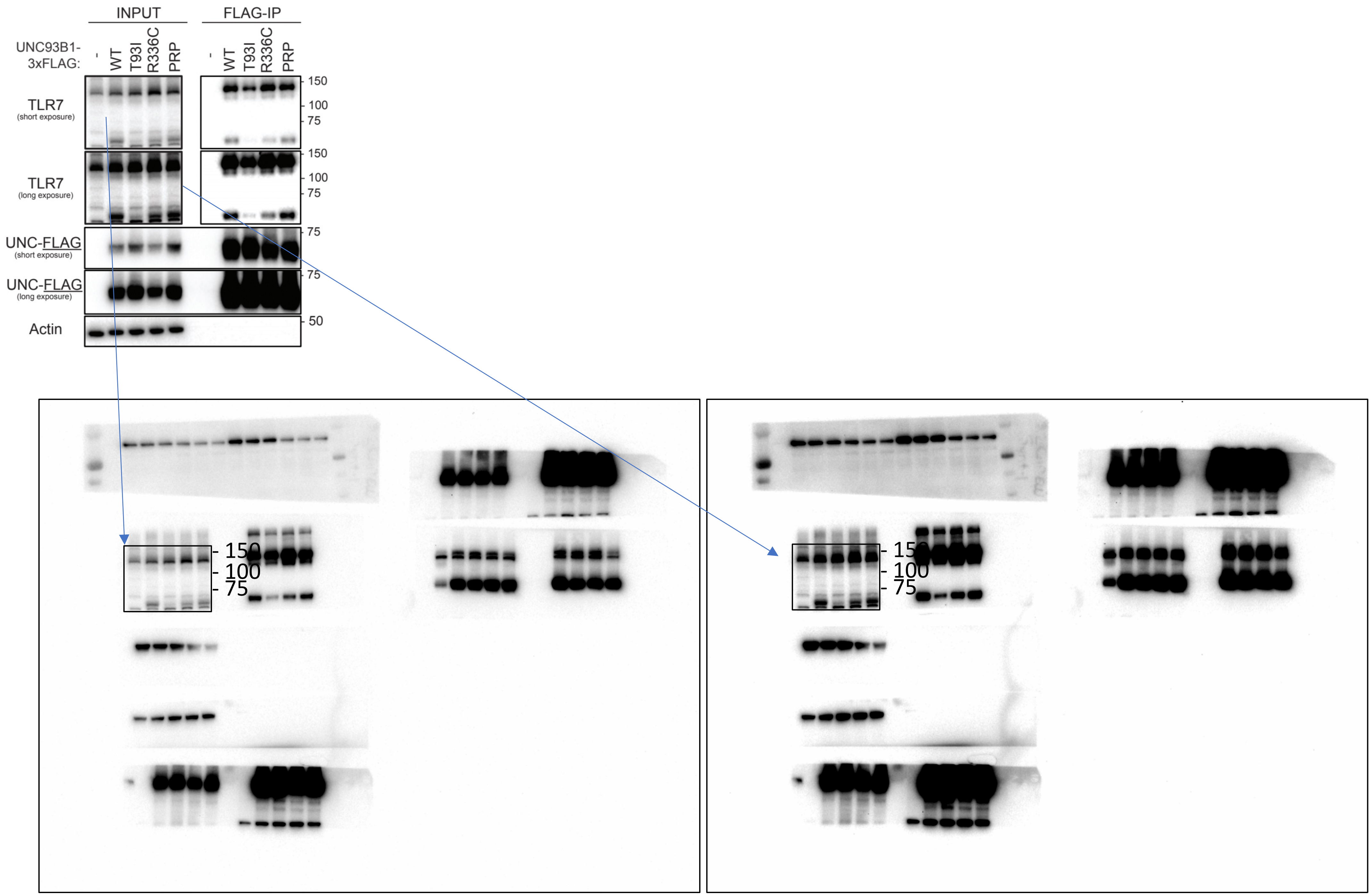

Figure 3D
Part 2

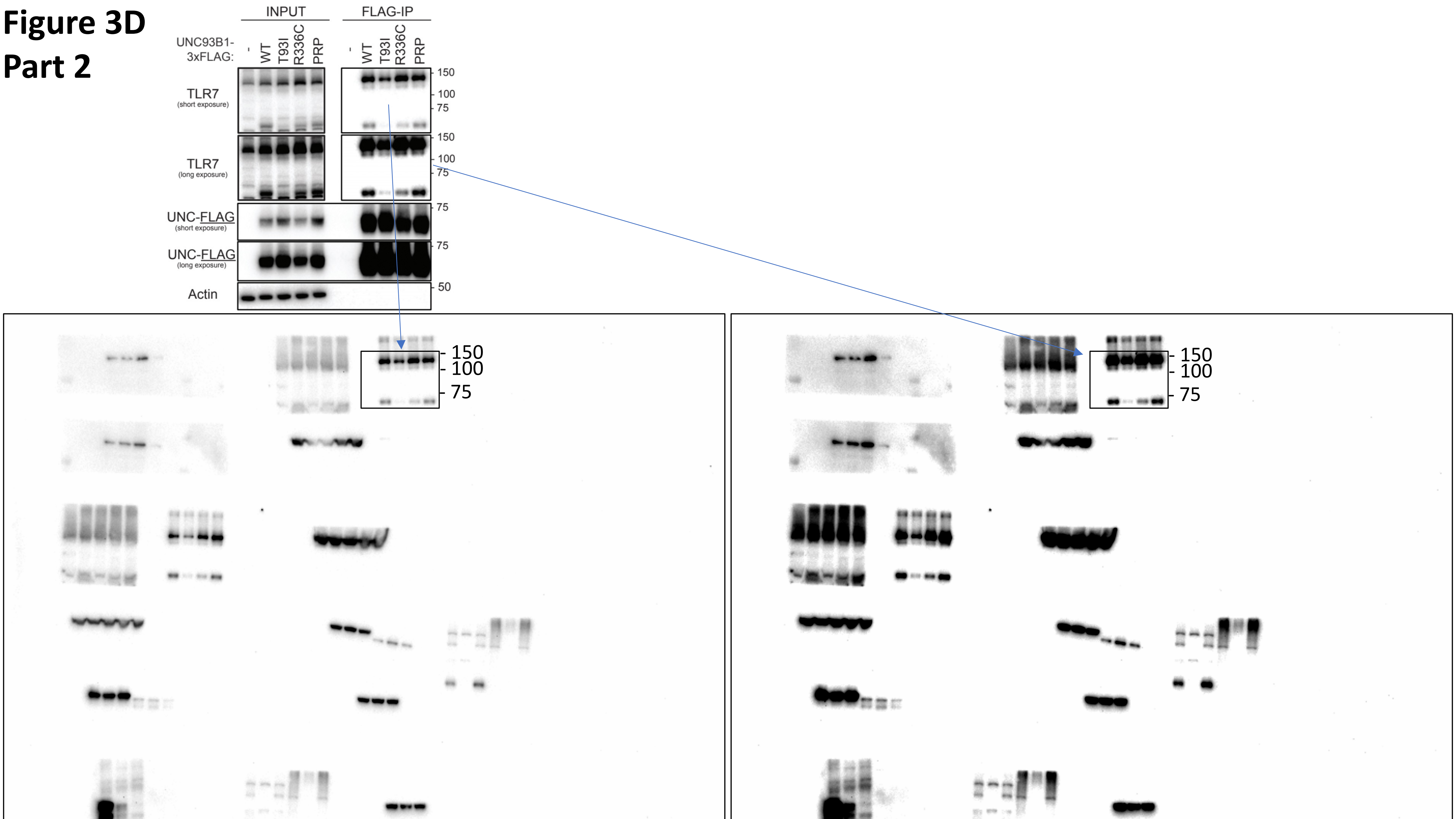

Figure 3D
Part 3

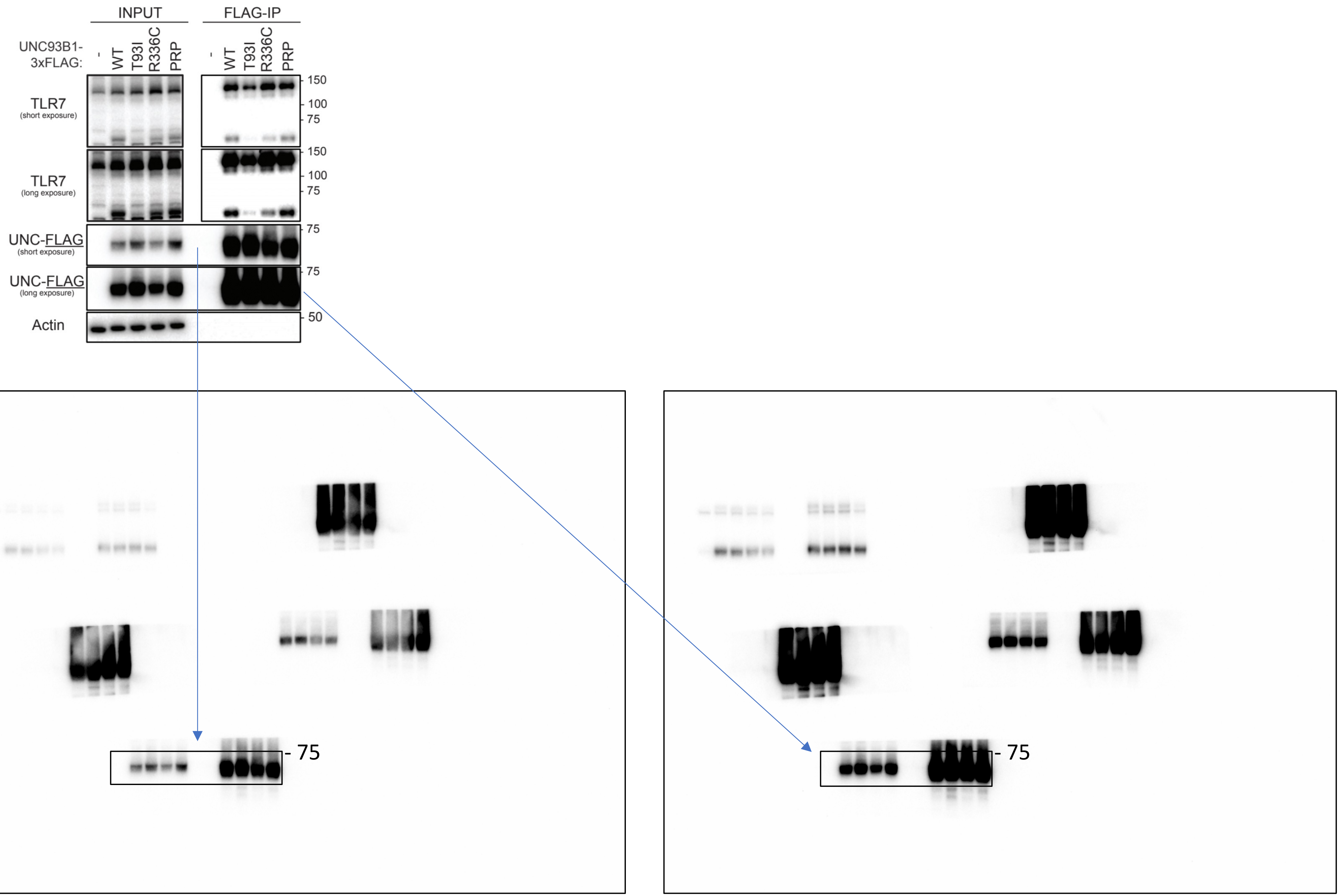

Figure 3D
Part 4

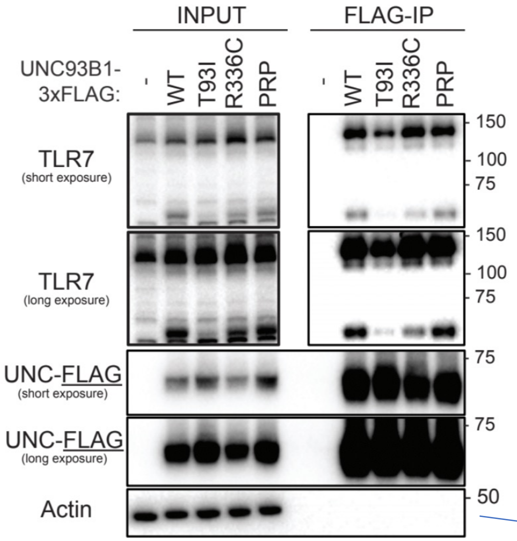

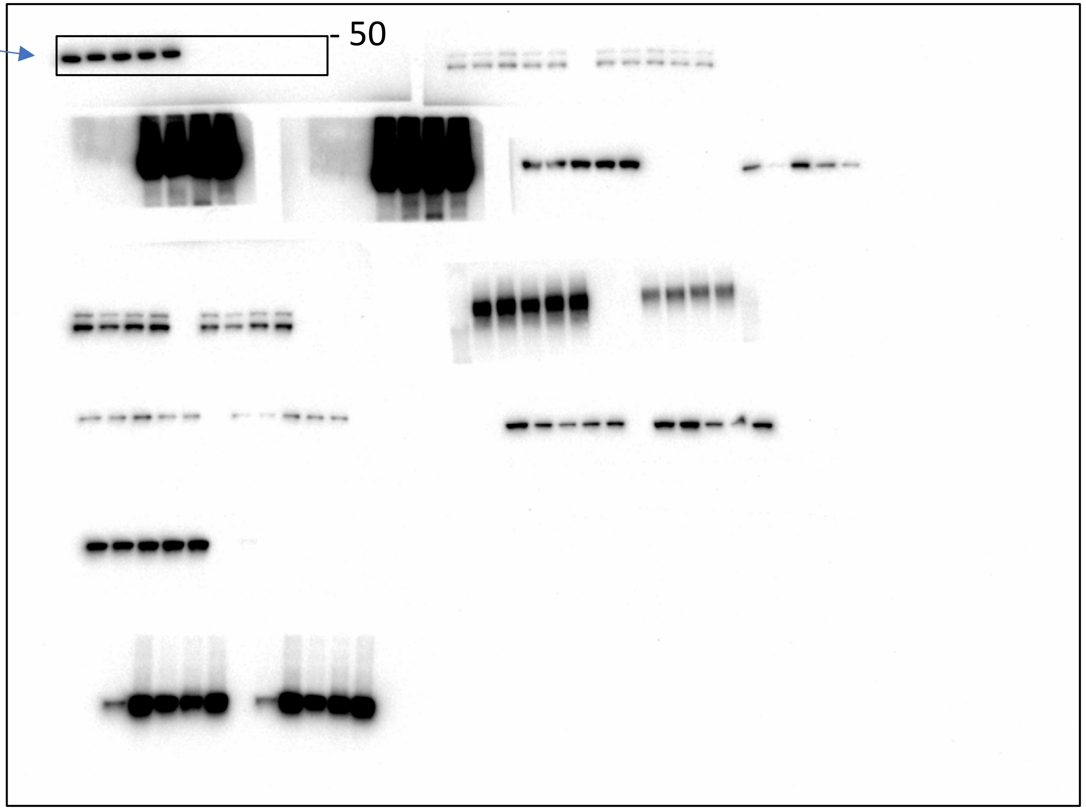

Figure 3E
Part 1

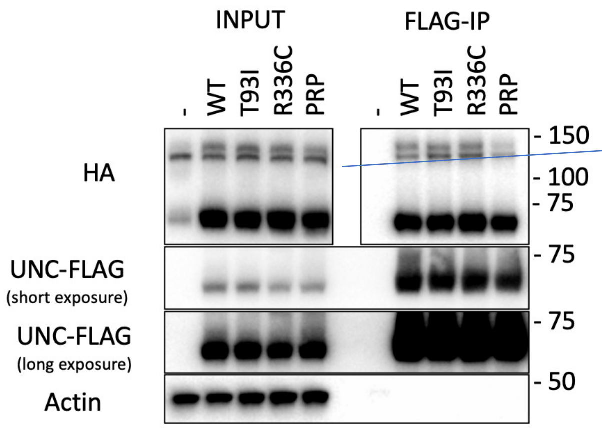

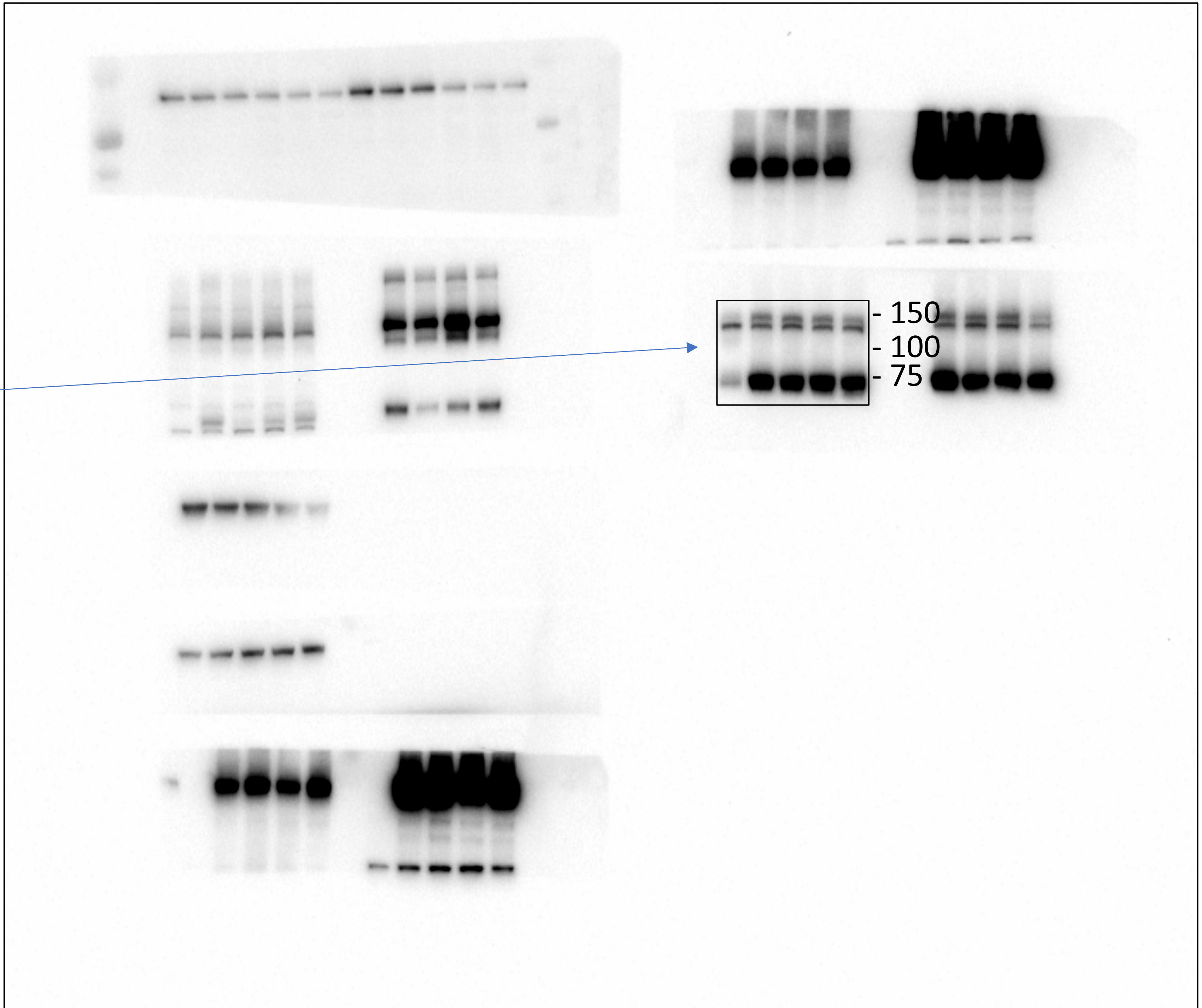

Figure 3E
Part 2

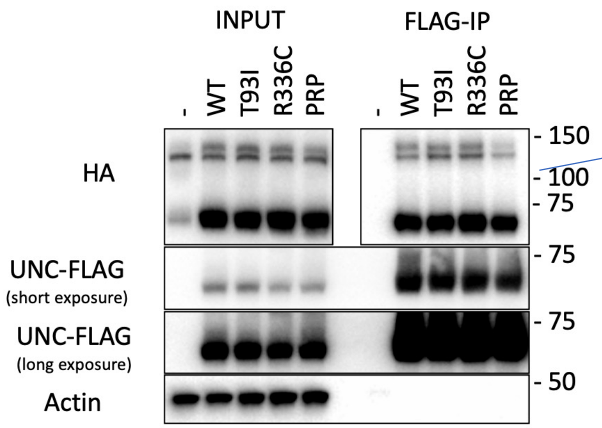

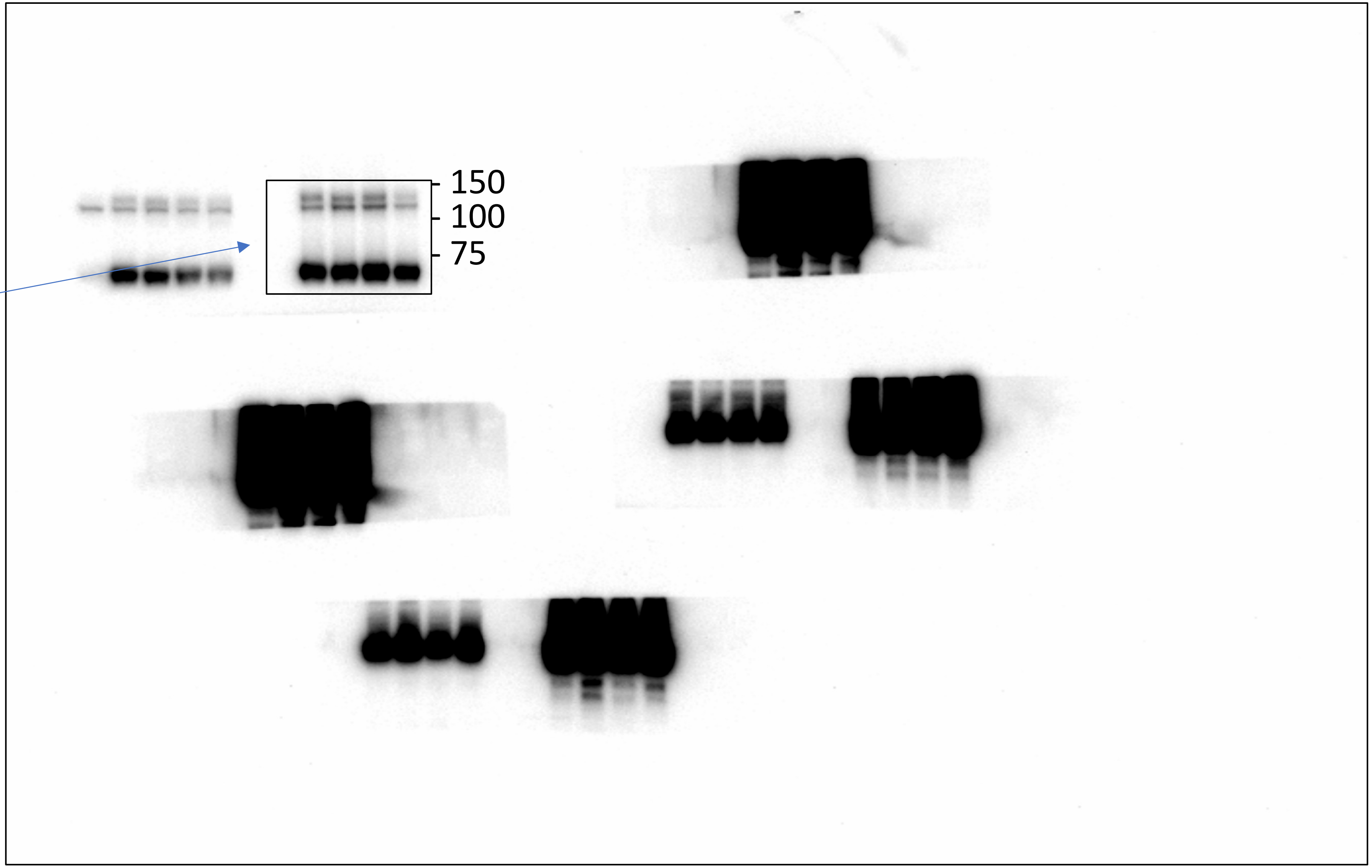

Figure 3E
Part 3

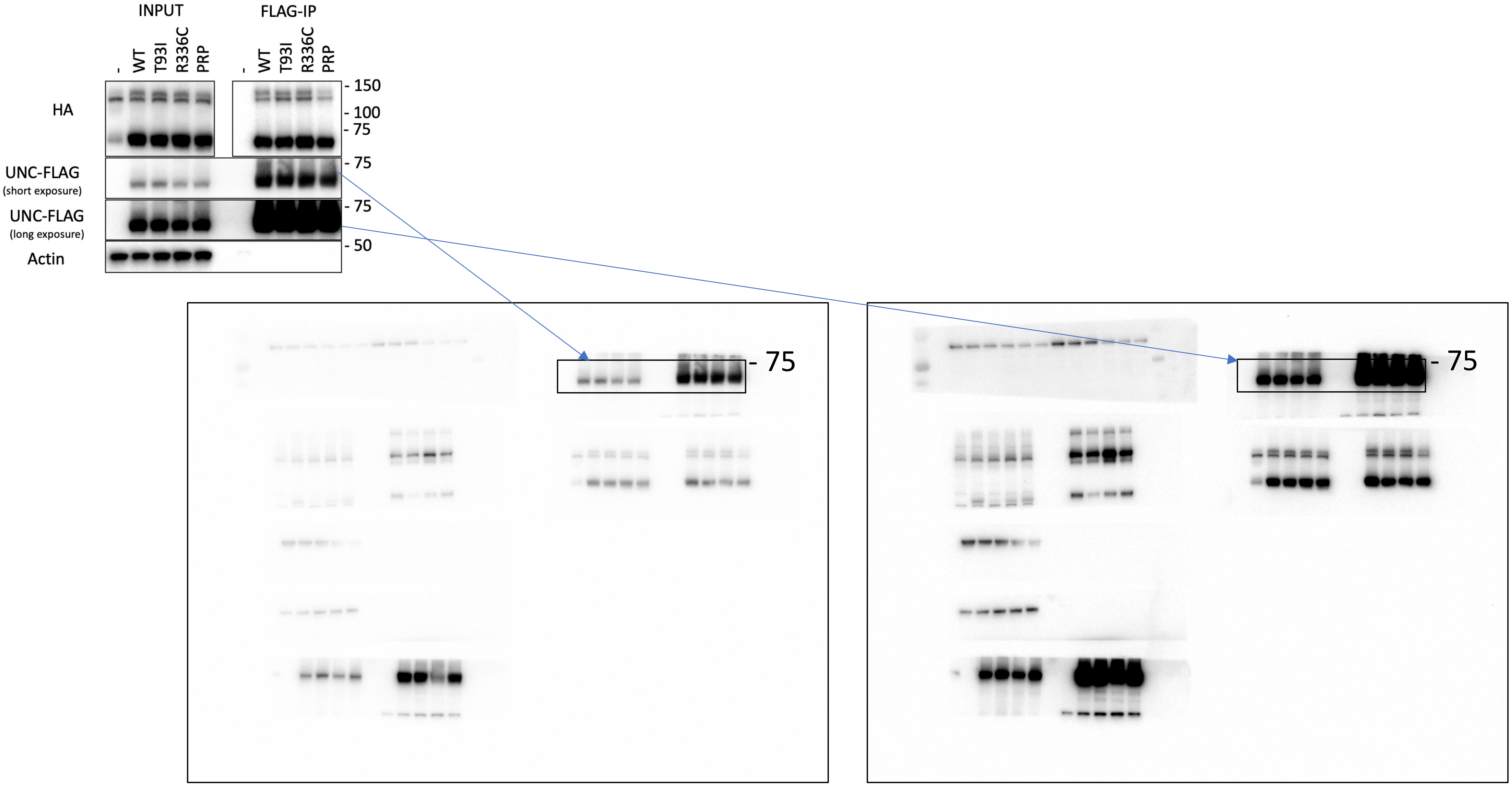

Figure 3E
Part 4

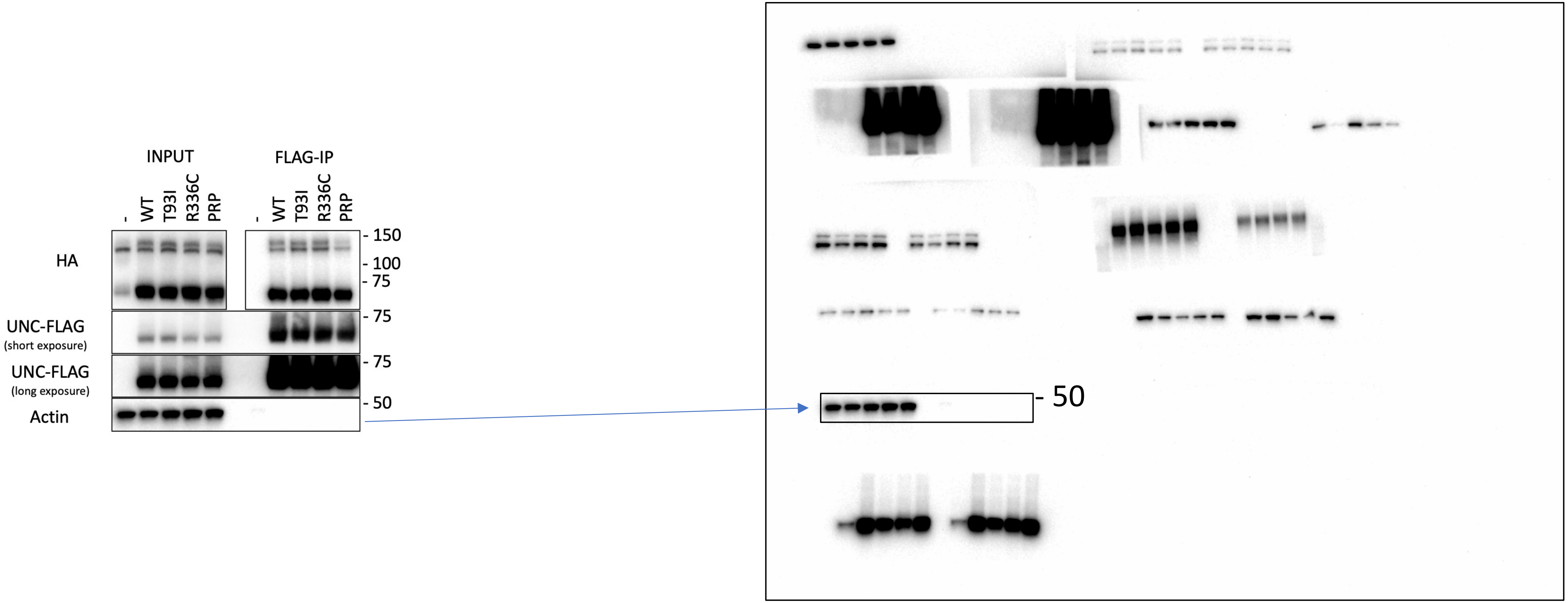

Figure 3F
Part 1

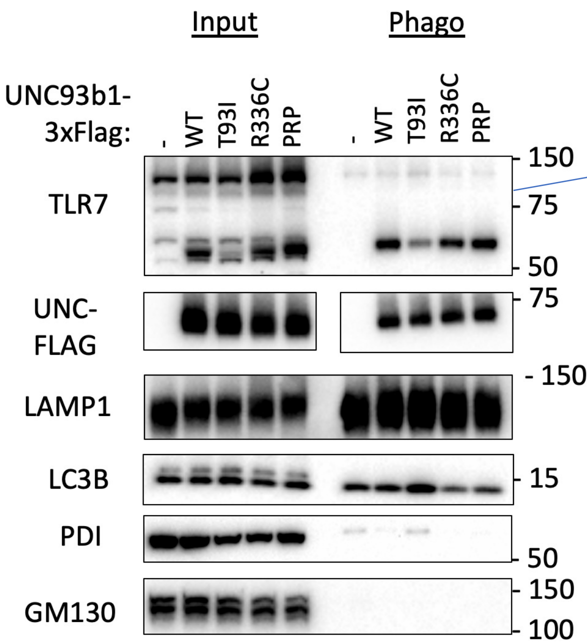

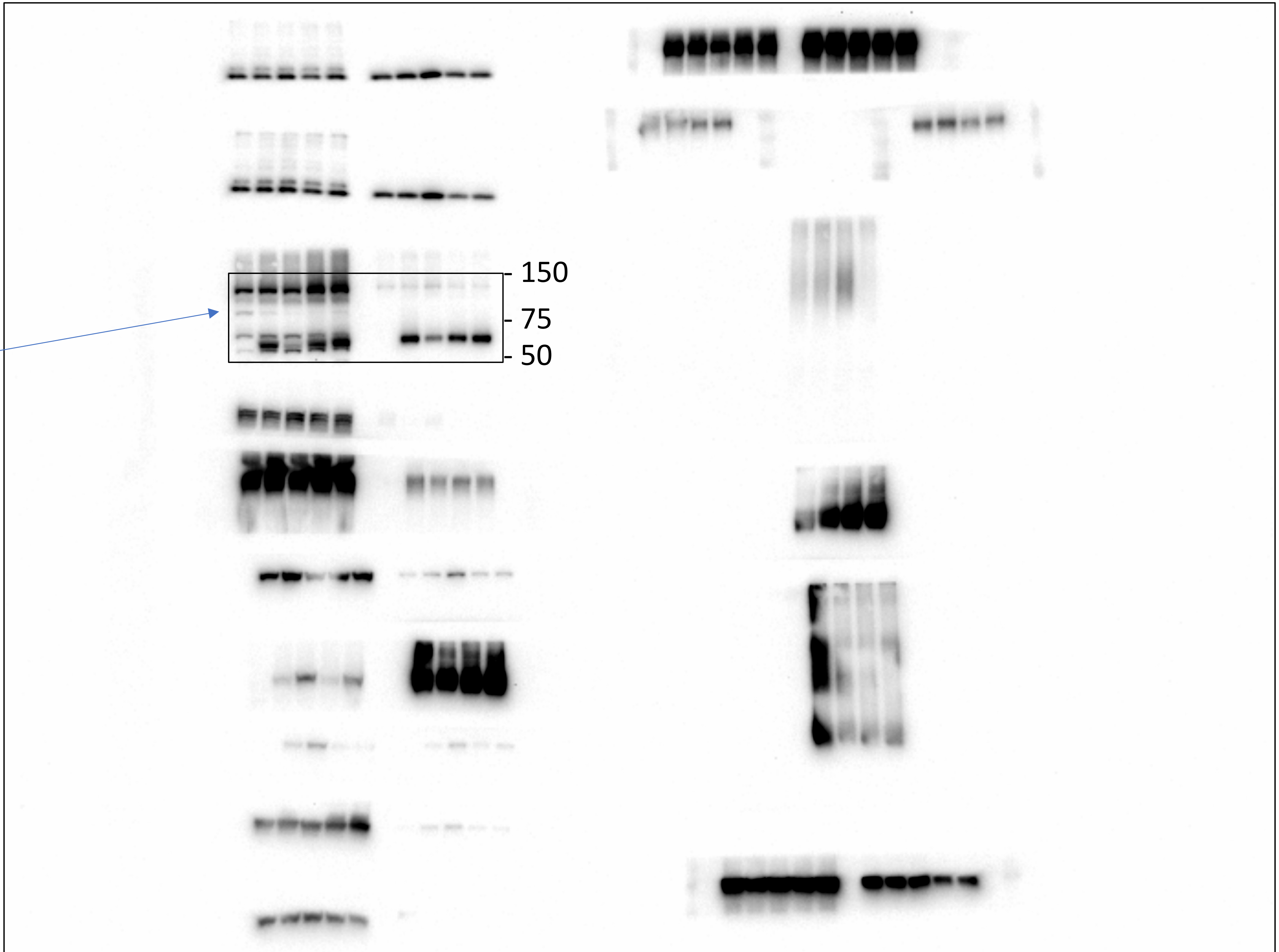

Figure 3F
Part 2

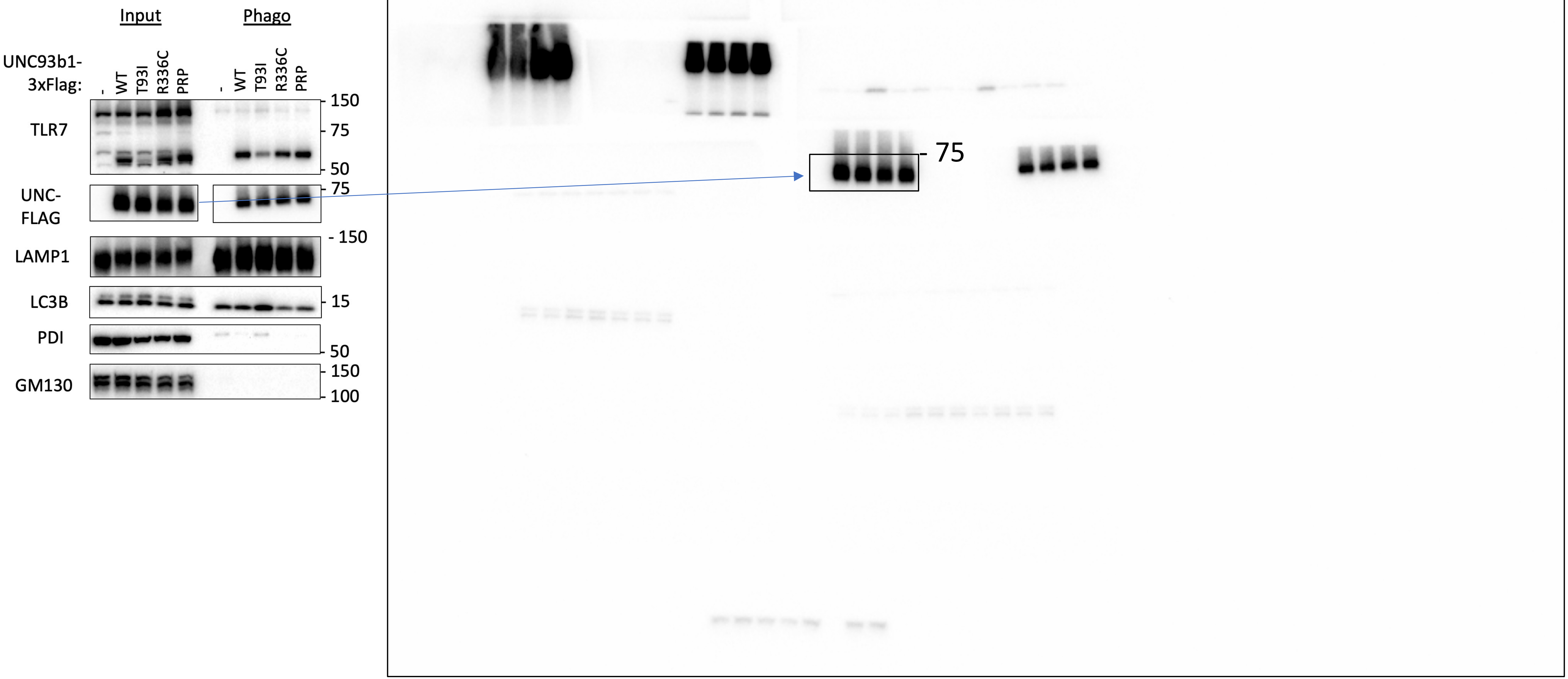

Figure 3F
Part 3

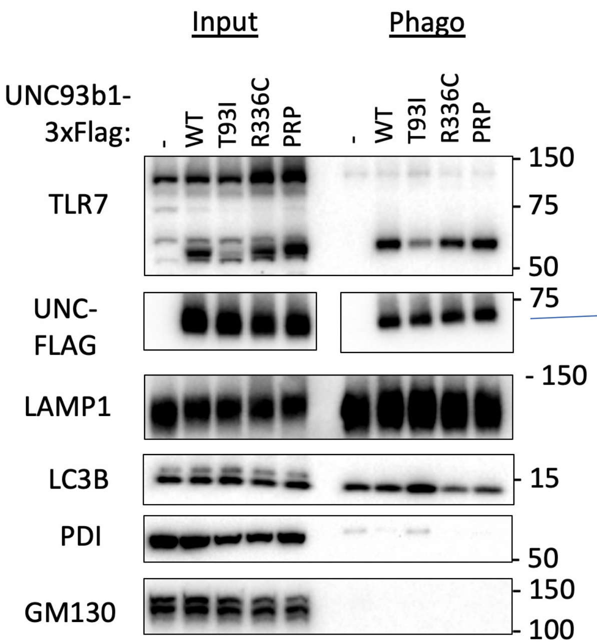

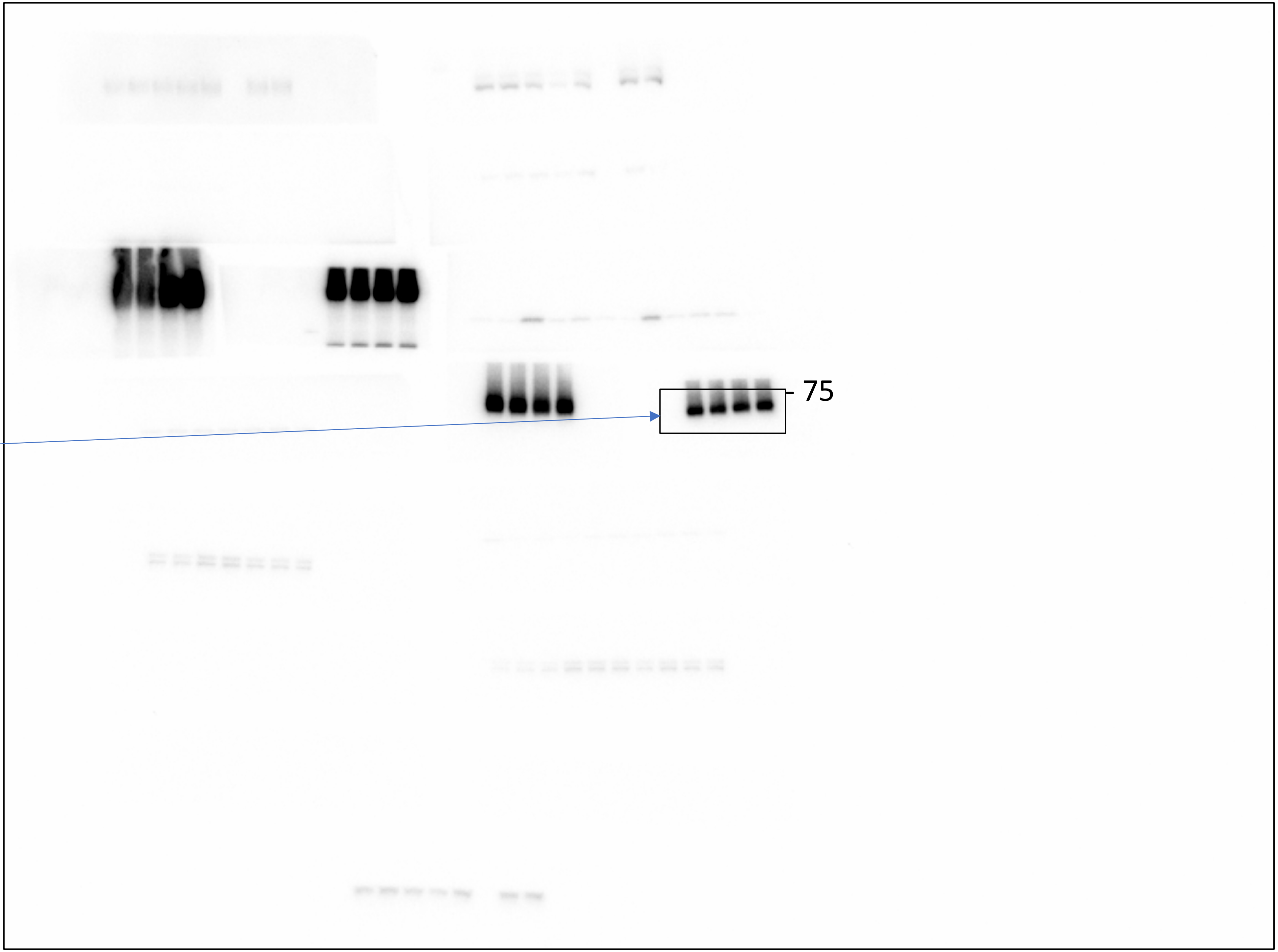

Figure 3F
Part 4

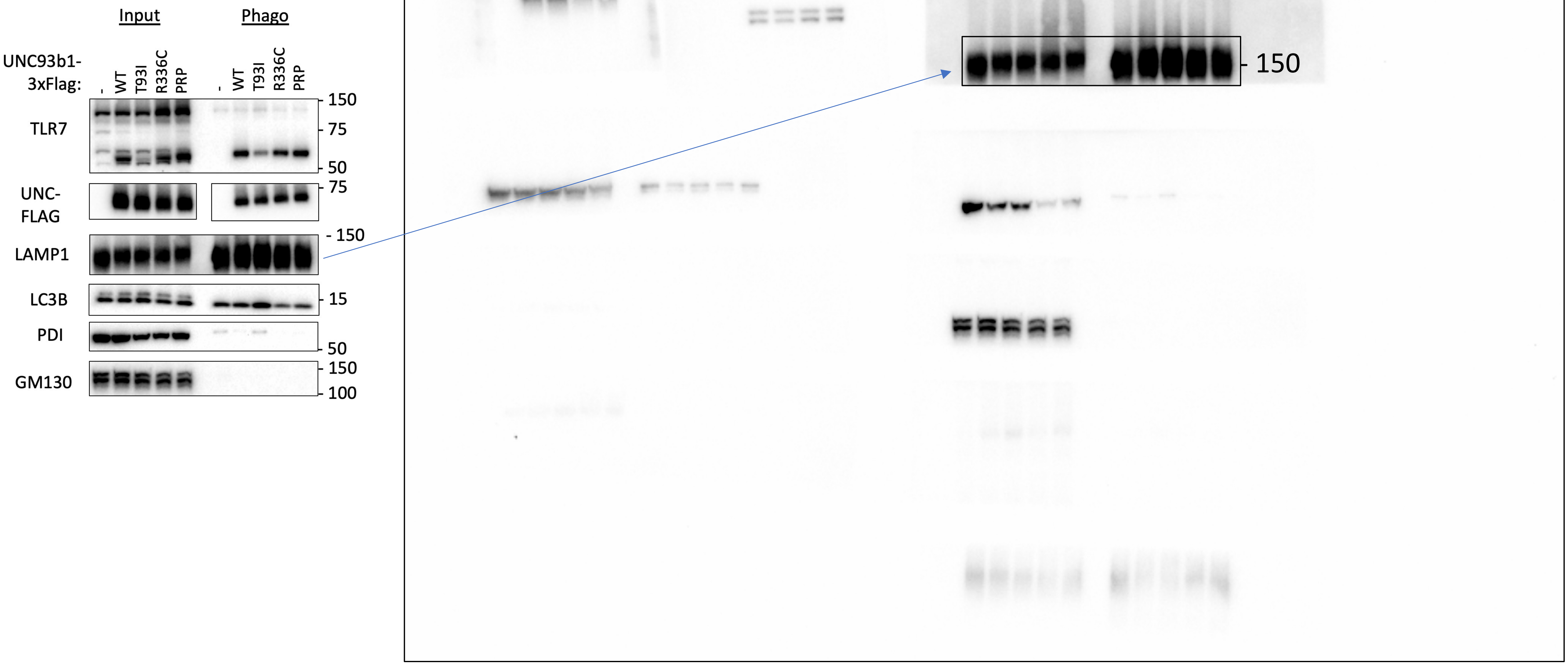

Figure 3F
Part 5

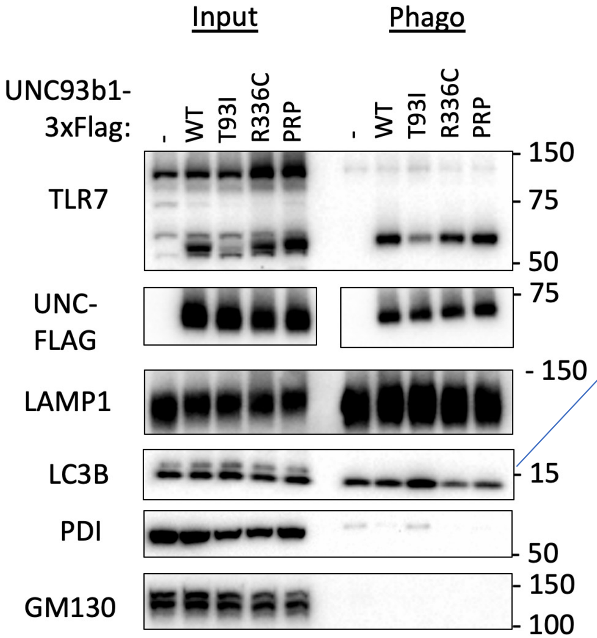

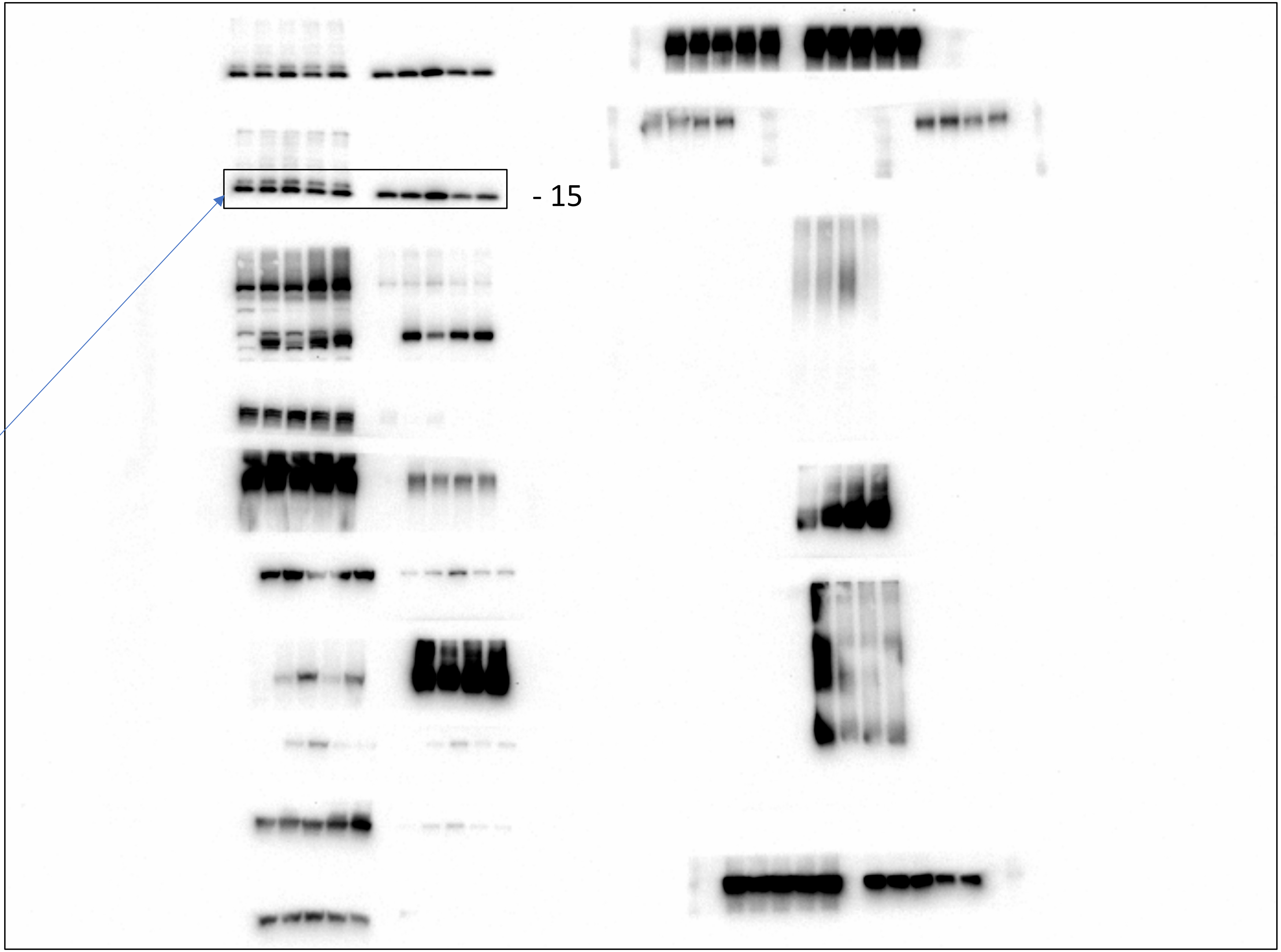

Figure 3F
Part 6

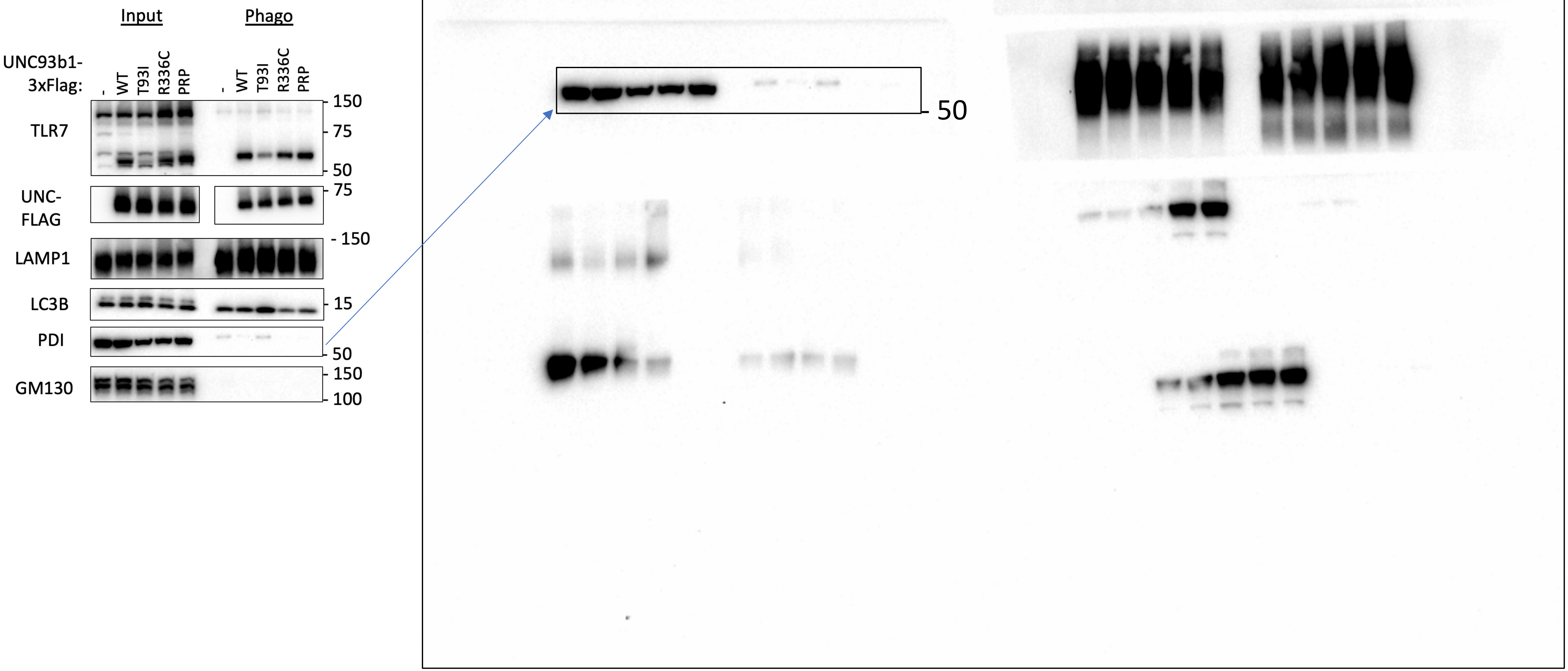

Figure 3F
Part 7

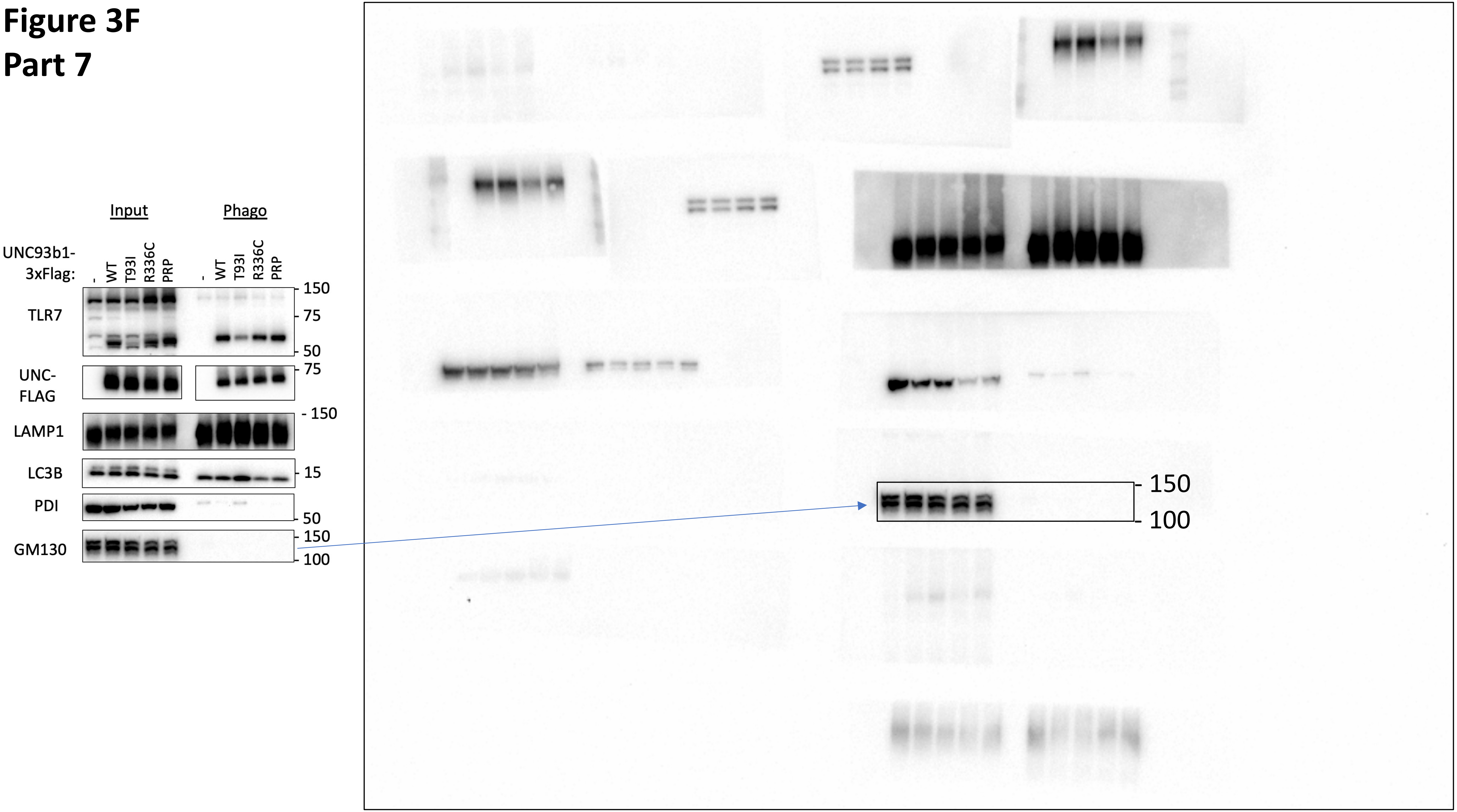

Supplement: SourceData F3 — contains original blots for Fig. 3. [file JEM_20232005_SourceDataF3.pdf]
